# Hybrid photoelectrocatalytic advanced oxidation systems for pharmaceutical wastewater treatment: mechanisms, reactive species, operational parameters, toxicity assessment and real wastewater treatment

**DOI:** 10.1039/d6ra06625a

**Published:** 2026-09-01

**Authors:** Salman Khan, Shohreh Azizi

**Affiliations:** a Department of Chemistry, Abdul Wali Khan University Mardan Mardan 23200 Pakistan; b UNESCO-UNISA Africa Chair in Nanosciences and Nanotechnology, College of Graduate Studies, University of South Africa Muckleneuk Ridge, PO Box 392 Pretoria 0002 South Africa azizis@unisa.ac.za

## Abstract

Pharmaceutical micropollutants are persistent contaminants of aquatic systems, with a global survey of 258 rivers across 74 countries detecting pharmaceutical residues on every continent and 25% of sampling sites exceeding safe ecological thresholds. Conventional wastewater treatment provides incomplete removal, necessitating intensified technologies. This review critically evaluates hybrid photoelectrocatalytic (PEC) advanced oxidation processes (AOPs), focusing on PEC coupled with peroxymonosulfate (PMS), peroxydisulfate (PDS), H_2_O_2_, electro-Fenton, ozonation, and biological processes. Reported hybrid systems achieved pharmaceutical removal efficiencies of approximately 75–100%. Representative PEC/PMS systems attained 98% sulfamethoxazole removal within 90 min, 100% norfloxacin removal within 25 min, and 91.3% tetracycline removal within 30 min. PEC/PDS enhanced bisphenol A degradation from 65.0% to 85.9% within 60 min, while optimized PDS-assisted treatment produced substantially faster degradation kinetics. In PEC/H_2_O_2_ systems, 95% tetracycline removal was achieved within 90 min, whereas electrogenerated H_2_O_2_ increased the phenol degradation rate constant from 0.136 to 2.568 h^−1^. Ozone-assisted PEC achieved 96% cefadroxil removal and 57.6% TOC reduction, while a pilot-scale solar photoelectro-Fenton/ozone process achieved 60% pharmaceutical degradation and 41% COD removal in a four-drug mixture. Bio-PEC hybrids demonstrated 95–99.04% pharmaceutical removal and up to 93% lower energy consumption. These improvements arise from enhanced charge separation and coordinated generation of ˙OH, SO_4_˙^−^, O_2_˙^−^, and ^1^O_2_. Nevertheless, catalyst deactivation, oxidant consumption, mass-transfer limitations, incomplete mineralization, transformation-product toxicity, and energy demand remain critical barriers. Overall, hybrid PEC-AOPs offer highly tunable platforms for pharmaceutical wastewater treatment, but standardized energy, toxicity, mineralization, and long-term stability assessments under real-water conditions are required for practical scale-up.

## Abstract 2

Pharmaceutical micropollutants are persistent contaminants of aquatic systems, with a global survey of 258 rivers across 74 countries detecting pharmaceutical residues on every continent and 25% of sampling sites exceeding safe ecological thresholds.

## Graphical abstract

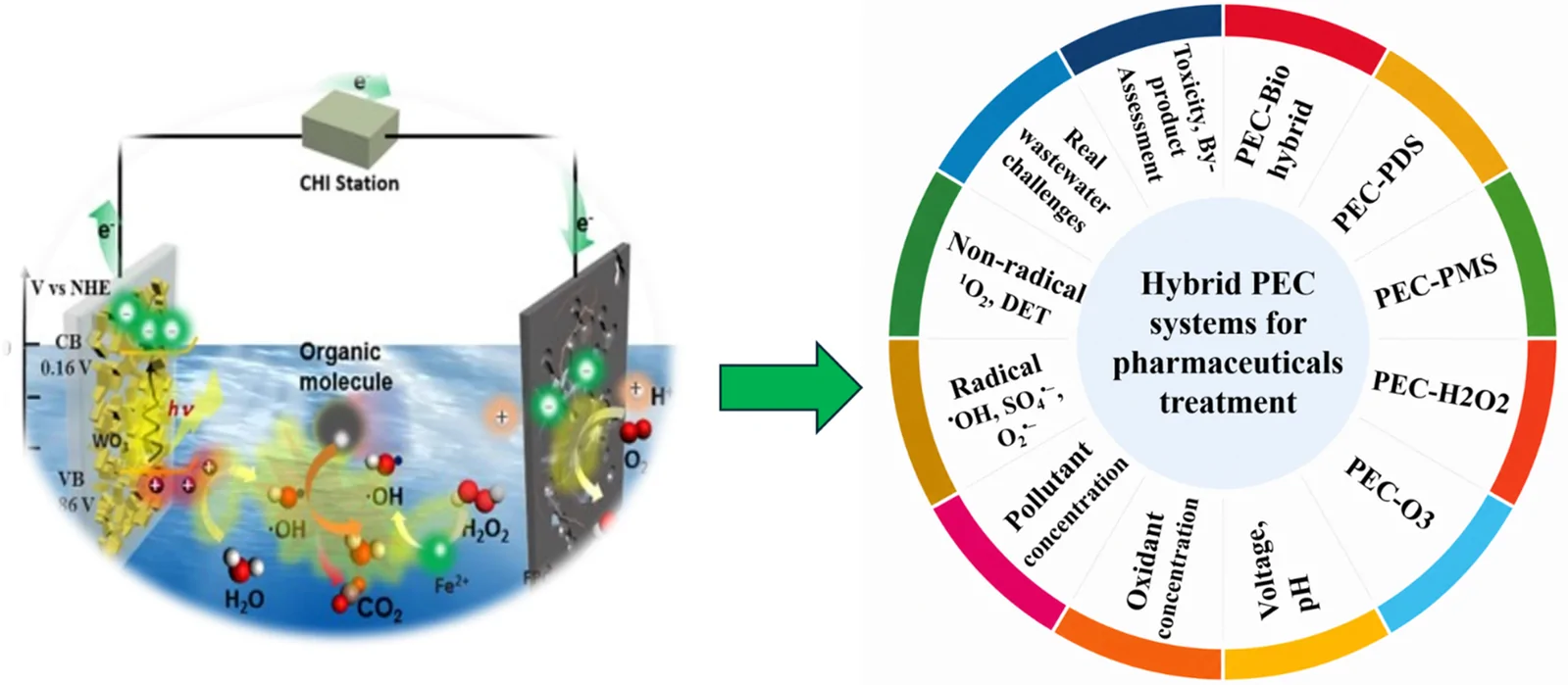

## 1. Introduction

Water pollution has emerged as one of the most critical global environmental challenges of the 21st century. Although approximately 71% of the Earth's surface is covered by water, only about 2.5% is freshwater.^1,2^ Moreover, less than 1% of the available freshwater is readily accessible for human consumption, while a substantial proportion is stored in glaciers, ice caps, and groundwater reserves. Consequently, only a very small fraction of the planet total water resources is readily available to support human populations and ecosystems. The World Health Organization (WHO) has identified contaminated water as a significant contributor to global mortality, particularly in regions where access to safe drinking water remains limited.^3^ More than 2.3 billion people currently experience water stress, and the global demand for water is projected to approach a 40% supply deficit by 2030.^4^ The rapid expansion of industrialization, intensive agricultural activities, and urbanization has further exacerbated the deterioration of natural water resources.^5,6^ Among the diverse classes of water contaminants, emerging organic pollutants (EOPs) including pharmaceuticals, personal care products, endocrine-disrupting chemicals, and pesticides have attracted increasing scientific and environmental concern because of their persistence, potential for bioaccumulation, and adverse biological effects, even at trace concentrations. Pharmaceutical compounds are now ubiquitous in the global aquatic environment, driven by ever-increasing human and veterinary use.^7,8^ Worldwide, trillions of doses of drugs are consumed each year, and a large fraction of active ingredients is excreted or discarded unmetabolized into sewage.^9^ For example, in the United States one-third of 4 billion annual prescriptions (1.3 billion items) go unused and enter waste streams.^10,11^ Over 2.3 billion people face water stress, and global water demand may exceed supply by nearly 40% by 2030.^12^ Aging populations and rising healthcare needs have driven global pharmaceutical market growth, further compounding the release of drugs into water.^13,14^ Veterinary and agricultural use of antimicrobials is even larger, over 70% of all antibiotics sold in some countries are used in livestock or aquaculture, and 70–80% of these can leach directly into surface waters.^15^ As a result, trace pharmaceuticals including hormones, analgesics, antibiotics, *etc.* have been routinely detected in surface and ground water across the globe.^16^ In fact, a recent worldwide survey of 258 rivers in 74 countries reported pharmaceutical contamination on every continent, and 25% of sampling sites contained drug concentrations above safe ecological thresholds.^17^ Reported concentrations in raw wastewater often reach the 10^4^–10^5^ ng L^−1^ range (*e.g.* diclofenac, ibuprofen, sulfamethoxazole has been measured at >100 000 ng L^−1^ in STP influent), and even treated effluents still carry detectable amounts (from ng L^−1^ to µg L^−1^) of dozens of compounds.^18–20^ Such pervasive contamination is linked to ecological harm *e.g.* feminization of fish by contraceptive hormones and public health concerns like antimicrobial resistance.

Conventional Wastewater Treatment Plants (WWTPs) are not designed to effectively remove these micropollutants under high loads.^21,22^ Most municipal WWTP are mechanical–biological processes (*e.g.*, activated sludge) that were not originally intended to treat trace organics.^23–25^ Consequently, pharmaceuticals largely pass through unchanged. One study found removal efficiencies spanning 0% (*e.g.* contrast agents) to 97% (for some stimulants), but many drug classes saw only low removal *i.e.* antibiotics 50%, common analgesics and anti-inflammatories only 30–40%.^26^ In practice this means the majority of WWTPs discharge notable fractions of their influent drugs. For instance, in a UK national monitoring program, 13% of large WWTPs were predicted to release effluents exceeding predicted no-effect concentrations for one or more APIs.^27^ Numerous field studies worldwide consistently find pharmaceuticals present in both influents and effluents. In fact, surveillance studies in Europe, Asia and North America report 50–90% detection frequencies for common drugs in treated wastewater.^18^ Alarmingly, negative removal is often observed, as some compounds desorb or are formed (metabolites, byproducts) during treatment.^28,29^ A recently identified example of emerging contaminants that often exhibit negative removal efficiencies in STPs is diazepam, carbamazepine, azithromycin, and clindamycin.^19^ In a recent study on six WWTPs (200 000 population each), the concentration detected in the influent was in the range of 7–1019 ng L^−1^, and it was 9–2266 ng L^−1^ in the effluent, while only a few, such as naproxen and ketoprofen, were effectively removed.^18^ Such observations highlight the fact that a substantial portion of pharmaceutical loading is still not removed through conventional secondary treatment, and consequently, the rivers and reuse sources are contaminated.

Pharmaceutical removal by advanced oxidation processes (AOPs) has been developed due to the limitations of conventional WWTPs (Table 1). AOPs can produce reactive radicals such as hydroxyl radicals (˙OH), which can degrade recalcitrant organics non-selectively.^30–32^ Examples of AOPs are Fenton/photo-Fenton reactions, ozone, UV/H_2_O_2_, persulfate activation, and photocatalysis. Such techniques have been extensively investigated with respect to the degradation of micropollutants in the past 20 years. The treatment of pharmaceutical residues by AOPs is promising and efficient.^33^ Kinetics can be further enhanced, and the reactive spectrum expanded, by combining two or more chemical types of AOP, such as combining UV with H_2_O_2_ or Fenton.^34^ There has been a significant increase in research into AOP (numerous publications and pilot projects in antibiotic-contaminated streams).

**Table 1:** Comparative overview of major AOPs for pharmaceutical degradation, highlighting their target contaminants, key advantages, limitations, and practical wastewater-treatment applications

AOP	Representative target pharmaceuticals	Key advantages	Major limitations	Practical applications
Heterogeneous photocatalysis	Antibiotics, analgesics, anti-inflammatory drugs	Strong oxidative capability; uses photogenerated electron–hole pairs; potentially solar-driven	Rapid electron–hole recombination; limited visible-light utilization; catalyst recovery	Tertiary wastewater treatment and pharmaceutical micropollutant polishing
UV/H_2_O_2_	Antibiotics, analgesics, persistent pharmaceuticals	Efficient ˙OH generation; relatively simple process configuration	High energy and oxidant requirements; H_2_O_2_ scavenging at excessive concentrations; matrix interference	Advanced tertiary treatment and micropollutant polishing
Fenton/photo-Fenton	Antibiotics, analgesics, and recalcitrant pharmaceuticals	Strong ˙OH production; rapid oxidation; suitable for refractory contaminants	Narrow pH operating window; iron sludge generation; iron management and reagent consumption	Industrial and municipal wastewater treatment
Ozonation	Antibiotics, cefadroxil, ciprofloxacin, and other pharmaceuticals	High oxidation capacity; effective toward electron-rich pharmaceutical structures; readily integrated with other AOPs	Ozone-generation energy demand; mass-transfer limitations; incomplete mineralization and transformation products	Tertiary municipal wastewater treatment and micropollutant removal
PMS/PDS activation	Sulfamethoxazole, tetracycline, and other antibiotics	Generates SO_4_˙^−^ and ˙OH; enables radical and non-radical oxidation pathways; broad operating flexibility	Oxidant consumption; scavenging by water-matrix components; potential residual oxidant and sulfate concerns	Treatment of recalcitrant pharmaceutical contaminants
Standalone PEC	Pharmaceutical micropollutants	Combines light and electrical bias; improved charge separation; controllable interfacial oxidation	Electrical energy demand; electrode/catalyst stability; limited ROS generation and mass-transfer constraints	Emerging tertiary treatment and modular treatment systems
Hybrid PEC-AOPs	Antibiotics, analgesics, and mixed pharmaceutical contaminants	Synergistic charge separation and ROS generation; combines complementary oxidation mechanisms; improved treatment flexibility	Greater system complexity; energy consumption; catalyst/electrode fouling; oxidant demand; limited long-term validation	High-performance tertiary treatment, decentralized systems, and future continuous-flow applications

Heterogeneous photocatalysis is of specific interest within the scope of AOP, due to its relatively simple operation.^35^ In classic photocatalysis, UV photons are absorbed by the semiconductor, exciting the electrons to a high energy level that crosses the semiconductor bandgap, creating powerful oxidants at the surface.^36,37^ The chemical stability, non-toxicity and low cost of solar or UV-based photocatalysis are the advantages of the technology.^38^ Indeed, heterogeneous photocatalysis has been coined as an environmentally friendly and cost-effective process. Its basic shortcomings, however, are well known. Most photocatalysts catalysts are only effective for the absorption of UV radiation (∼5% of the solar spectrum), and the photogenerated electron–hole pairs are very short-lived with a tendency to recombine rapidly.^39^ Besides, most photocatalysts are stationary materials, which must be separated and regenerated after post-treatment. The most serious drawbacks of the photocatalysis are low photocatalytic efficiency and poor stability, which are mainly caused by poor light absorption and high electron–hole recombination.^40,41^ Even under optimal lab conditions, in practice, the degradation of drugs using photocatalytic technology may take several hours and result in partial conversion, which presents a challenge for scaling.

To address these challenges, the field has been seeking an improved form of photocatalysis, known as photoelectrocatalysis (PEC). PEC is a photocatalytic electrode with an external electric bias, which gives electrochemical assistance for the separation of charges. The photoanode subjected to a positive bias in a PEC cell will create electron–hole pairs in the semiconductor and reduce the chance for the electron–hole pairs to recombine, thereby increasing the lifetime of the oxidizing hole.^42,43^ In effect, PEC combines semiconductor photochemistry with electrochemistry. This synergy produces much higher overall conversions than photocatalysis alone, because of the enhanced charge separation.^38^ As described PEC is an AOP combining light-driven semiconductor catalysis with an applied electrical bias to enhance charge separation and generate reactive species.^44^ In practice, PEC systems generate abundant radicals (˙OH, SO_4_˙^−^, *etc.*) at the anode surface under bias, directly attacking pharmaceuticals and convert to CO_2_, H_2_O and inorganic ions.^45^ Recent studies show that modest anodic bias can dramatically speed pharmaceutical breakdown compared to photocatalysis alone. For example, applying bias to a TiO_2_ photoanode in real wastewater significantly increased ˙OH production and gave near-complete oxidation of drug micropollutants.^46,47^ PEC can also offer beneficial side effects such as electrolytic disinfection, recovery of toxic metals at the cathode, *etc.*, which not only increase treatment efficiency but also improve kinetics. PEC is a potentially viable approach to address the ubiquitous portion of pharmaceuticals that remains unconverted.

Accordingly, current research increasingly focuses on hybrid PEC systems that integrate PEC with complementary treatment processes to exploit synergistic effects and enhance pollutant degradation efficiency. The *in situ* generation of H_2_O_2_ has been combined with the biased photoanode in the photoelectron-Fenton process, which further enhances the production of ˙OH.^48^ Similarly, PEC has been used in combination with the activation of persulfate (PMS/PDS) in complex systems.^49^ Furthermore, PEC is a relatively new technology; photocatalytic membranes or flow-by PEC–membrane reactors may be used to filter and oxidize wastewater at the same time. These hybrids make use of the PEC oxidative power and membrane selectivity. Self-cleaning of the membrane and degradation of the fouling substances were significantly enhanced in the textile wastewater treatment by the study of PEC/RO and PEC/UF hybrids using TiO_2_-based photocatalysts embedded in polymer membranes (*e.g.*, TiO_2_/PANI/PVDF).^38^ Under optimized conditions, there are significant improvements in the removal of dyes and pharmaceuticals for key PEC/membrane designs (post-treatment of RO permeate, treatment of RO concentrate, *etc.*). These designs take advantage of the surface acidity and generation of ˙OH of photocatalysts to lower the membrane fouling and even provide the antibacterial effect.^50,51^ Beyond membranes, PEC has been hybridized with bioelectrochemical systems and catalytic oxidation in novel photo-bioelectrochemical reactors for persistent drugs.^52,53^ Each hybrid approach aims to push conversion closer to 100%, often by *in situ* regenerating oxidants or combining steps *e.g.* oxidative pretreatment coupled with biodegradation.

The introduction of PEC and its hybrid represent a logical evolution from classic photocatalysis toward more robust AOPs. With the global pharmaceutical footprint continuing to grow and evidence that conventional treatment leaves tens of percent of drug loads untouched, advanced strategies are essential. PEC technology especially when coupled with complementary processes *i.e.* electro-Fenton, membranes, persulfate, *etc.* addresses the main weaknesses of photocatalysis such as recombination and narrow spectrum by adding external bias and hybridization.^54^ The following review will examine how hybrid PEC systems are being designed and assessed to close the gap in pharmaceutical removal, building on the trends outlined here.

Many of the quantitative trends noted above include global consumption forecasts, prescription waste percentages, and WTTP removal rates come from diverse reports and may vary regionally. While the cited studies reflect broad consensus, actual values can differ by country, season and methodology. The field also lacks standardized metrics for pharmaceutical load in wastewater. In addition, long-term pilot data for hybrid PEC systems are still scarce, so their real-world cost and scalability remain to be fully validated.

This review aims to provide a critical and integrated assessment of hybrid PEC advanced oxidation systems for the removal of pharmaceutical micropollutants from wastewater. The primary objective is to elucidate how coupling PEC with complementary oxidation and biological processes can overcome the limitations of conventional photocatalysis and standalone PEC. Particularly rapid electron–hole recombination, limited ROS generation, incomplete mineralization, and high energy requirements. The review focuses on hybrid PEC configurations involving PMS, PDS, H_2_O_2_, electro-Fenton, ozonation, and bio-electrochemical processes, with emphasis on their mechanisms, pollutant-removal performance, and practical applicability. The scope extends from semiconductor–electrolyte interfacial charge-transfer processes to ROS generation and transformation, including ˙OH, SO_4_˙^−^, O_2_˙^−^, ^1^O_2_, and high-valent metal-oxo species. Attention is given to the influence of pH, applied bias, electrolyte composition, oxidant dosage, light irradiation, and pollutant concentration on degradation kinetics. The review further evaluates degradation pathways, mineralization, transformation-product toxicity, catalyst/electrode stability, real wastewater performance, energy consumption, and scale-up challenges. The novelty of this review lies in integrating these aspects within a single framework rather than evaluating hybrid PEC technologies solely according to pollutant-removal efficiency. Specifically, it connects interfacial charge-transfer mechanisms and ROS engineering with operational conditions, toxicity outcomes, energy requirements, and real-water applicability. This approach enables identification of the key gaps preventing translation from laboratory-scale demonstrations to practical wastewater treatment.

## 2. Major classes of pharmaceuticals detected in wastewater

**Fig. 1:**
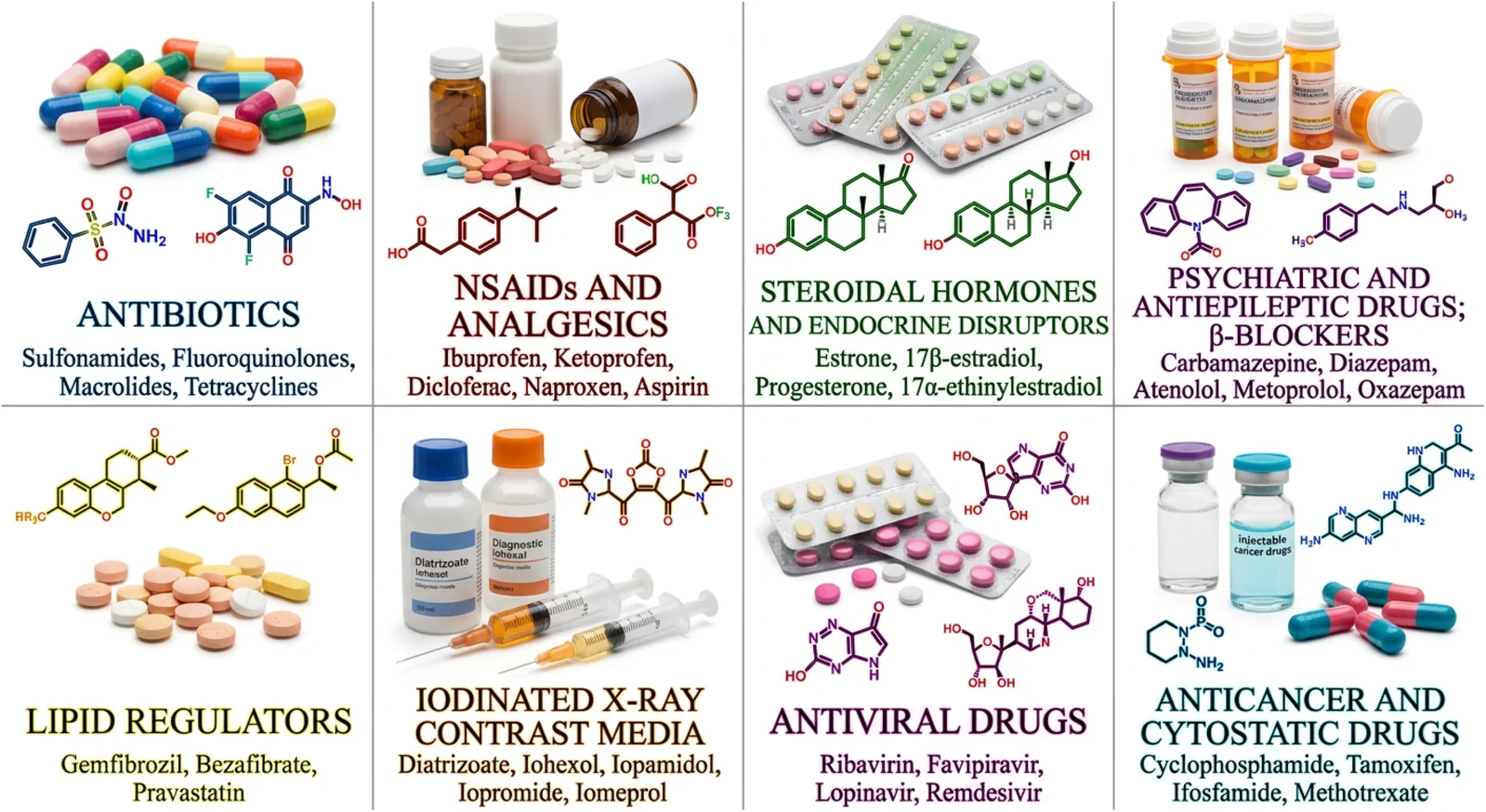
Representative compounds from the pharmaceutical classes discussed above: sulfamethoxazole (antibiotic), diclofenac and ibuprofen (NSAIDs), 17α-ethinylestradiol (hormone/EDC), carbamazepine (antiepileptic/psychiatric drug), atenolol (β-blocker), gemfibrozil (lipid regulator), iopamidol (iodinated contrast medium), and cyclophosphamide (anticancer/cytostatic agent).

Pharmaceutical residues in wastewater span several major therapeutic classes (Fig. 1). Each class enters wastewater through a different route, and each has reported concentration ranges that vary by orders of magnitude depending on the source *i.e.*, municipal, hospital, or pharmaceutical-manufacturing wastewater.

### 2.1 Antibiotics

Antibiotics are pharmaceuticals used to treat or prevent bacterial infections in humans and animals. They are grouped by chemical structure and mechanism of action into families such as sulfonamides, fluoroquinolones, macrolides, and tetracyclines. Sulfonamide antibiotics such as sulfamethoxazole contain a sulfonamide group (–SO_2_NH–) linking an aniline ring to a heterocyclic amine, a scaffold that structurally mimics *para*-aminobenzoic acid and thereby blocks bacterial folate synthesis.^55^ Fluoroquinolones such as ciprofloxacin are built around a fluorinated quinolone core bearing carboxylic acid, ketone, and a piperazine substituent.^56^ The conjugated, fluorinated ring system of fluoroquinolones is chemically stable, which is consistent with their persistence and high relative abundance. Both scaffolds carry ionizable groups that keep the molecules moderately polar and water-soluble under environmental pH conditions.

Because a large fraction of an administered dose is excreted unmetabolized, antibiotics are among the pharmaceutical classes most frequently detected in domestic, hospital, and industrial wastewater. In a survey of 12 municipal wastewater treatment plants in Dalian, China, total antibiotic concentrations ranged from 63.6 to 5404.6 ng L^−1^. Fluoroquinolones and sulfonamides were the most abundant classes, accounting for 42.2% and 23.9% of the total antibiotic concentration, respectively, followed by tetracyclines at 16.0% and macrolides at 14.8%. Sulfamethoxazole, erythromycin, clarithromycin, azithromycin, ofloxacin, and norfloxacin were the most frequently detected antibiotics; of these, the concentration of ofloxacin was the highest in most of influent (average concentration = 609.8 ng L^−1^) and effluent (average concentration = 253.4 ng L^−1^) samples.^57^ Pharmaceutical-manufacturing wastewater can contain far higher concentrations than municipal sources. For instance, Larsson *et al.*^58^ reported that effluent from a treatment plant serving about 90 bulk drug manufacturers in Patancheru, India, contained the antibiotic ciprofloxacin at concentrations up to 31 mg L^−1^. Twenty-one of the 59 pharmaceuticals analyzed in that effluent exceeded 1 µg L^−1^, and eleven of these exceeded 100 µg L^−1^. The estimated discharge load of ciprofloxacin from that plant, about 45 kg per day, was equivalent to the total amount of the drug consumed in all of Sweden, a country of nine million people, over an average five-day period.

### 2.2 NSAIDs and analgesics

Non-steroidal anti-inflammatory drugs (NSAIDs) are used to relieve pain, fever, and inflammation, mainly by inhibiting cyclooxygenase (COX) enzymes. Because many NSAIDs are available without a prescription, they are among the highest-volume pharmaceutical classes consumed worldwide. Diclofenac and ibuprofen both carry a carboxylic acid group attached to an aromatic ring, a feature shared by most NSAIDs.^59^ Diclofenac additionally has a dichlorophenyl ring joined to a second aromatic ring through a diarylamine bridge, while ibuprofen carries a simple isobutyl-substituted phenylpropionic acid chain.^60^ The carboxylic acid group ionizes at environmental pH, which increases water solubility and mobility, while the aromatic rings remain reactive sites during oxidative water treatment. Their high consumption, combined with incomplete removal during conventional wastewater treatment, makes them common wastewater contaminants. Based on statistical data of Grand View Research, the global market for NSAIDs medications was estimated at nearly 20 billion dollars in 2021 and is projected to increase at a compound annual growth rate of 5.36% from 2022 to 2030.^61^ Representative compounds include ibuprofen, ketoprofen, diclofenac, naproxen, and aspirin. These compounds have been detected in wastewater at concentrations up to 2 747 000 ng L^−1^, equivalent to about 2747 µg L^−1^.^61^ Their high consumption and incomplete removal during conventional treatment make them among the most frequently detected pharmaceutical classes worldwide.

### 2.3 Steroidal hormones and endocrine disruptors

Steroidal hormones include natural hormones, such as estrone, 17β-estradiol, and progesterone, and synthetic hormones, such as 17α-ethinylestradiol, the active ingredient of oral contraceptives. All steroidal hormones share a tetracyclic cyclopentanoperhydrophenanthrene (steroid) ring system, visible in the 17α-ethinylestradiol structure. In estrogens, the A-ring is aromatic and carries a phenolic hydroxyl group, a feature required for binding to the estrogen receptor.^62^ The synthetic ethynyl substituent at the C17 position of ethinylestradiol is not present in natural estradiol and increases the molecule's resistance to metabolic and microbial degradation, consistent with its greater environmental persistence relative to natural estrogens.^63^

These compounds are excreted by humans and livestock largely as inactive conjugates that can be reactivated to their biologically active free form during wastewater treatment. Even at very low concentrations, they can interfere with the endocrine and reproductive systems of aquatic organisms. Estrogens have been reported in wastewater effluent at concentrations of 0.1 to 196 ng L^−1^. Progesterone has been reported at less than 0.1 to 439 ng L^−1^, while androgens have been reported at 0.06 to 86 ng L^−1^ and glucocorticoids have been reported at less than 0.1 to 433 ng L^−1^.^64^ In a study of seven municipal wastewater treatment plants in Tehran, Iran, influent concentrations of estrone, 17β-estradiol, and 17α-ethinylestradiol ranged from 6.54 to 18.76 ng L^−1^, 1.02 to 8 ng L^−1^, and 4.18 to 11.76 ng L^−1^, respectively. In the corresponding effluents, concentrations ranged from 1.04 to 4.99 ng L^−1^, 0.5 to 2.20 ng L^−1^, and 0.5 to 2.58 ng L^−1^, respectively.^65^

### 2.4 Psychiatric and antiepileptic drugs; β-blockers

Carbamazepine is an antiepileptic and mood-stabilizing drug, and diazepam is a benzodiazepine used to treat anxiety. Cardiovascular β-blockers such as atenolol and metoprolol are used to treat hypertension and related cardiovascular conditions.^66^ Carbamazepine consists of a tricyclic dibenzazepine ring system bearing a carboxamide substituent.^67^ The fully conjugated, fused aromatic rings confer high chemical and photolytic stability, consistent with the near-complete resistance to biological transformation. Atenolol shares the aryloxypropanolamine backbone typical of β-blockers: an aromatic ring connected through an ether oxygen to a secondary-amine-bearing propanol chain.^68^ The secondary amine is protonated at environmental pH, which increases water solubility and helps explain the frequent detection of β-blockers in treated effluent.

All of these drugs are prescribed at high volumes and excreted largely as the unchanged parent compound, making them common and persistent wastewater contaminants. Carbamazepine is one of the most persistent pharmaceuticals found in wastewater. It passed through both membrane bioreactor and conventional activated-sludge treatment essentially untransformed in one comparative study. In hospital wastewater treatment plant influent, carbamazepine and its transformation products have been reported at 0.03 to 7.33 µg L^−1^. In municipal sewage treatment plant effluent, concentrations of 0.003 to 1.26 µg L^−1^ have been reported.^69^ In a US survey of 50 large wastewater treatment plants, carbamazepine was detected in over 90% of effluent samples, together with the β-blockers metoprolol and atenolol.^70^ Across eight wastewater treatment plants in Skåne, Sweden, metoprolol was the largest single contributor to a combined annual discharge of about 71 kg of 21 monitored pharmaceuticals, with carbamazepine, naproxen, and the anxiolytic oxazepam also present at significant levels.^71^

### 2.5 Lipid regulators and iodinated X-ray contrast media

Lipid-regulating drugs, including gemfibrozil, bezafibrate, and pravastatin, are prescribed to lower blood cholesterol and triglyceride levels.^72,73^ Their carboxylic-acid- or statin-based structures make them moderately polar and only partially removed by biological wastewater treatment. Gemfibrozil consists of a dimethylphenoxy group linked to an isobutyric acid bearing two methyl substituents on the carbon adjacent to the carboxylic acid.^74^ This quaternary-like, sterically hindered carbon limits enzymatic and microbial attack on the acid group, consistent with the relatively steady effluent concentrations of fibrate drugs reported above. Gemfibrozil, bezafibrate, and pravastatin are frequently detected in wastewater.

Iodinated X-ray contrast media are radiopaque compounds administered in large single doses for diagnostic imaging procedures.^75^ They are excreted almost entirely unmetabolized within one to two days of administration, and their high polarity and chemical stability make them resistant to both biological and conventional oxidative treatment. Iopamidol is built on a triiodinated benzene ring substituted with multiple amide side chains bearing hydroxyl groups. The three iodine atoms are strongly electron-withdrawing, which makes the aromatic ring resistant to electrophilic radical attack, while the hydroxylated amide side chains impart very high-water solubility.^76^ This combination of an oxidation-resistant aromatic core and a highly polar periphery explain both the poor removal of contrast media by conventional treatment and their high mobility in the aquatic environment. Representative compounds include diatrizoate, iohexol, iopamidol, iopromide, and iomeprol. Across 51 studies reviewed from North America, Asia, and Europe, hospital wastewater concentrations of these compounds ranged from 0.01 to 3800 µg L^−1^, with the highest concentrations reported for iohexol, iopamidol, and iomeprol.^76^

### 2.6 Antiviral, anticancer and cytostatic drugs

Antiviral drugs are used to treat viral infections such as influenza, HIV, and COVID-19. Demand for specific antivirals rises sharply during disease outbreaks, and because these drugs are often excreted largely unmetabolized, wastewater concentrations can spike accordingly during epidemic periods. Acyclovir is a guanine nucleoside analogue, a purine base linked to an acyclic hydroxyethoxymethyl side chain in place of the natural ribose sugar ring.^77^ This modified side chain allows the molecule to be recognized by viral enzymes while remaining chemically distinct from natural nucleosides, and its terminal hydroxyl group contributes to its high-water solubility. One review estimated the occurrence and ecotoxicological risk of antiviral drugs used against COVID-19 in wastewater and environmental waters. Predicted secondary-effluent concentrations included 7402 ng L^−1^ for ribavirin, 4231 ng L^−1^ for favipiravir, 730 ng L^−1^ for lopinavir, and 319 ng L^−1^ for remdesivir. Conventional wastewater treatment was estimated to remove less than 20% of about half of the antiviral substances assessed.^78^ Anticancer and cytostatic drugs are used in chemotherapy to kill or slow the growth of cancer cells, often through DNA-alkylating or antimetabolite mechanisms.^79,80^ Because these mechanisms are not selective to cancer cells, even trace environmental concentrations raise concern for genotoxic and cytotoxic effects on non-target organisms. Cyclophosphamide contains a six-membered oxazaphosphorine ring bearing two chloroethyl substituents on the exocyclic nitrogen.^81^ These chloroethyl groups are the reactive alkylating centers that cross-link DNA in target cells, and this same electrophilic reactivity underlies the genotoxicity concerns raised for non-target organisms exposed to cyclophosphamide in the environment. A systematic review of 75 studies identified cyclophosphamide, tamoxifen, ifosfamide, and methotrexate as the most commonly studied compounds. Reported concentrations of anticancer drugs in the aquatic environment ranged from 0.01 to 86 200 ng L^−1^.^82^

## 3. Fundamentals of photoelectrocatalysis

**Fig. 2:**
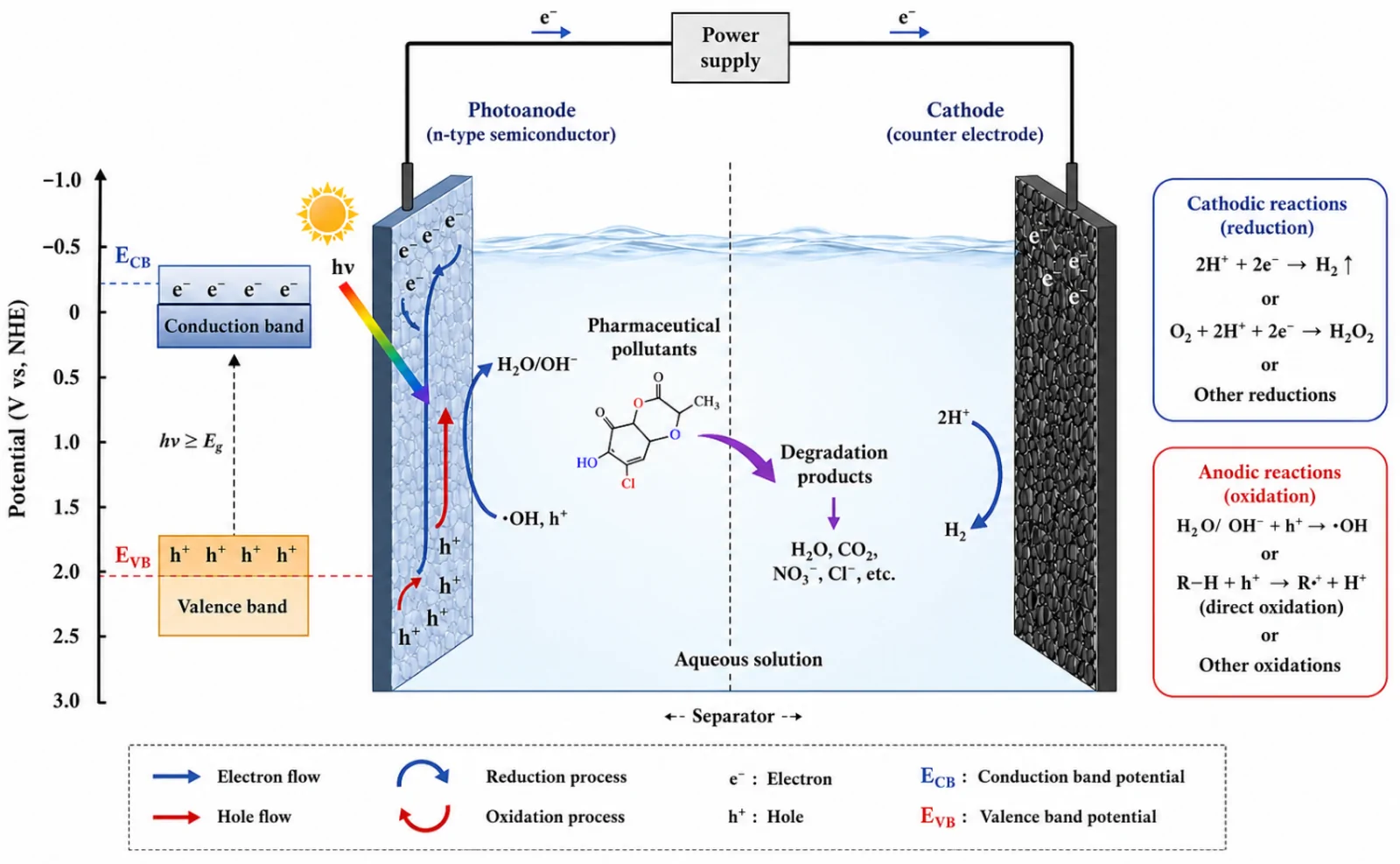
Fundamental mechanism of PEC degradation of pharmaceutical pollutants in an n-type semiconductor PEC cell.

PEC merges semiconductor photochemistry with electrochemical driving forces. When a semiconductor photoelectrode is illuminated by light of energy ≥ its bandgap, electrons (e^−^) are excited from the valence band into the conduction band, leaving behind holes (h^+^) (reaction (1)).^83–85^ In a typical PEC cell using an n-type photoanode, these photo-generated holes migrate to the electrode surface and oxidize water or other adsorbed molecules, while the electrons are drawn through an external circuit to a counter electrode (cathode) where they reduce species (*e.g.* protons to hydrogen) (Fig. 2).^86^ This process can be understood as two complementary half-reactions at the anode, water oxidation yields oxygen (reaction (2)), and at the cathode protons are reduced to hydrogen (reaction (3)). The key effect of coupling the photocatalytic film to an external circuit is that it suppresses recombination of the e^−^/h^+^ pair, greatly increasing the efficiency of charge utilization.^87,88^ The photocurrent is the mechanism by which PEC draws away electrons, and increases the availability of holes, increasing the effectiveness of the PEC for driving redox reactions compared to photocatalysis alone.^89,90^

The conduction band edge needs to be at a sufficiently negative potential (*vs.* the chosen reference, such as the NHE or RHE) to reduce the target species, while the valence band edge needs to be sufficiently positive to oxidize water or organic pollutants.^91–94^ Careful band alignment is important to PEC design; the semiconductor Fermi level gives the flat band potential in PEC design, and there are space charge layers and Helmholtz layers at the solid/liquid interface.^95,96^ Band bending under illumination allows for charge separation, holes will move to the surface, and electrons will move towards the bulk or external circuit.^88,97^ This band bending is further modified by the applied bias, which can shift the relative energies and make the interfacial energy barriers lower to be overcome by the photo-generated carriers.^98^

The use of an external potential to the semiconductor film is the hallmark of PEC and has a significant impact on charge dynamics. The application of a positive bias on the n-type photoanode increases the positive charge of the semiconductor and thus increases the band bending at the interface.^99^ This enhanced band bending causes the depletion layer to widen and creates a significant electric field inside, which causes the electrons generated by the light to move away from the interface to the back contact and the holes to move toward the surface.^100–102^ Bias is a force that forces electrons to flow in the direction of the semiconductor rather than recombine with holes. The external bias is responsible for the separation of electrons and holes in PEC and reduces the recombination of electrons and holes, thereby enhancing the overall efficiency of the conversion.^103^ Increasing the bias voltage further accelerates this separation. In terms of the photocurrent, higher biases yield higher photocurrents and more complete oxidation of organics up to the point where the transport or kinetics become limiting.^104,105^ To sum up, when the applied bias is applied, the semiconductor Fermi level moves closer to the electrochemical potential of the cathode, thus bringing the semiconductor bands in closer alignment with one another and making the photocarriers more readily available for reactions at the interfaces.

The anodic and cathodic reaction pathways in PEC systems are determined by the nature of the semiconductor and the solution chemistry. In a photoanodic PEC cell (common for water splitting and oxidation of organics), the main anodic half-reaction is usually water oxidation or pollutant oxidation at photogenerated holes.^106^ For example, in an acidified aqueous electrolyte under illumination, holes oxidize water (reaction (2)) yield molecular oxygen and releases protons and electrons. Concurrently, at the cathode the electrons from the external circuit reduce protons to hydrogen gas (reaction (3)). These two reactions together constitute solar water splitting. When photocatalytic degradation of an organic contaminant is targeted, the anodic reaction involves hole-driven oxidation of the pollutant. For example, a generic organic (RH) can be oxidized by holes or ˙OH at the photoanode (reaction (4)), while the cathodic half-reaction typically remains proton reduction to H_2_ or reduction of dissolved O_2_ (reaction (5)).

1 Semiconductor + *hν* → h_VB_^+^ + e_CB_^−^

2 2H_2_O + 4h^+^ → O_2_ + 4H^+^

3 4H^+^ + 4e^−^ → 2H_2_

4 Oxidation by hole: RH + h^+^ + H_2_O → R˙ + H_3_O^+^

5 Oxygen reduction to water: O_2_ + 4H^+^ + 4e^−^ → 2H_2_O

In many PEC oxidation studies, the focus is on the anodic half (mineralizing the pollutant) where the electrons at the cathode may produce H_2_ if protons are present or simply reduce residual O_2_ to water.

Interfacial charge transfer processes are central to PEC efficiency. After excitation, charges must be transferred across the semiconductor/electrolyte interface without recombining.^107^ The kinetics of these transfers depend on the surface chemistry, catalyst loading, and presence of co-catalysts or surface states. Photogenerated holes can react directly with adsorbed species or generate ˙OH *via* oxidation of water/OH^−^, the latter are extremely reactive and drive much of the mineralization of organics.^108,109^ Meanwhile, electrons either travel through the circuit to a counter electrode or, in a photocathode configuration, directly reduce species at the same electrode.^110^ Slow charge transfer leads to accumulation of charge carriers and recombination losses.^111,112^ Hence, a rapid charge separation at the interface should be ensured in a good PEC system. Some of the strategies employed involve modification of photoelectrodes into nano-structured or textured surfaces and the introduction of electron or hole scavengers, as well as the optimization of the electrolyte pH in order to enhance the surface kinetics.

These charge transfer processes are reflected by key performance parameters of PEC systems. The photocurrent density (*J*_ph_) under illumination at a certain bias is a direct indicator of the number of photocarriers that can be extracted.^113^ Other figures of merit include the incident photon-to-current efficiency (IPCE or EQE), the percentage of absorbed photons that are converted to current at each wavelength, and the applied-bias photon-to-current efficiency (ABPE), which takes into account both electrical and optical energy inputs.^88,114,115^ The onset potential can be used to indicate the ease with which the photoelectrode will proceed with the desired reaction the more negative the onset potential, the lower the overpotential.^116,117^ In applications such as hydrogen generation or pollutant mineralization, the faradaic efficiency (FE), which is the ratio of photocurrent to target chemical product, is important.^87,118^ Finally, stability under illumination and bias is also a performance parameter, which decreased by photocorrosion or catalyst degradation. The optimization of a PEC system demands the matching of semiconductor band structure and appropriate bias to promote the highest separation of the photogenerated carriers, resulting in a high rate of both anodic and cathodic reactions with minimal losses.

## 4. Classification of hybrid PEC systems

**Fig. 3:**
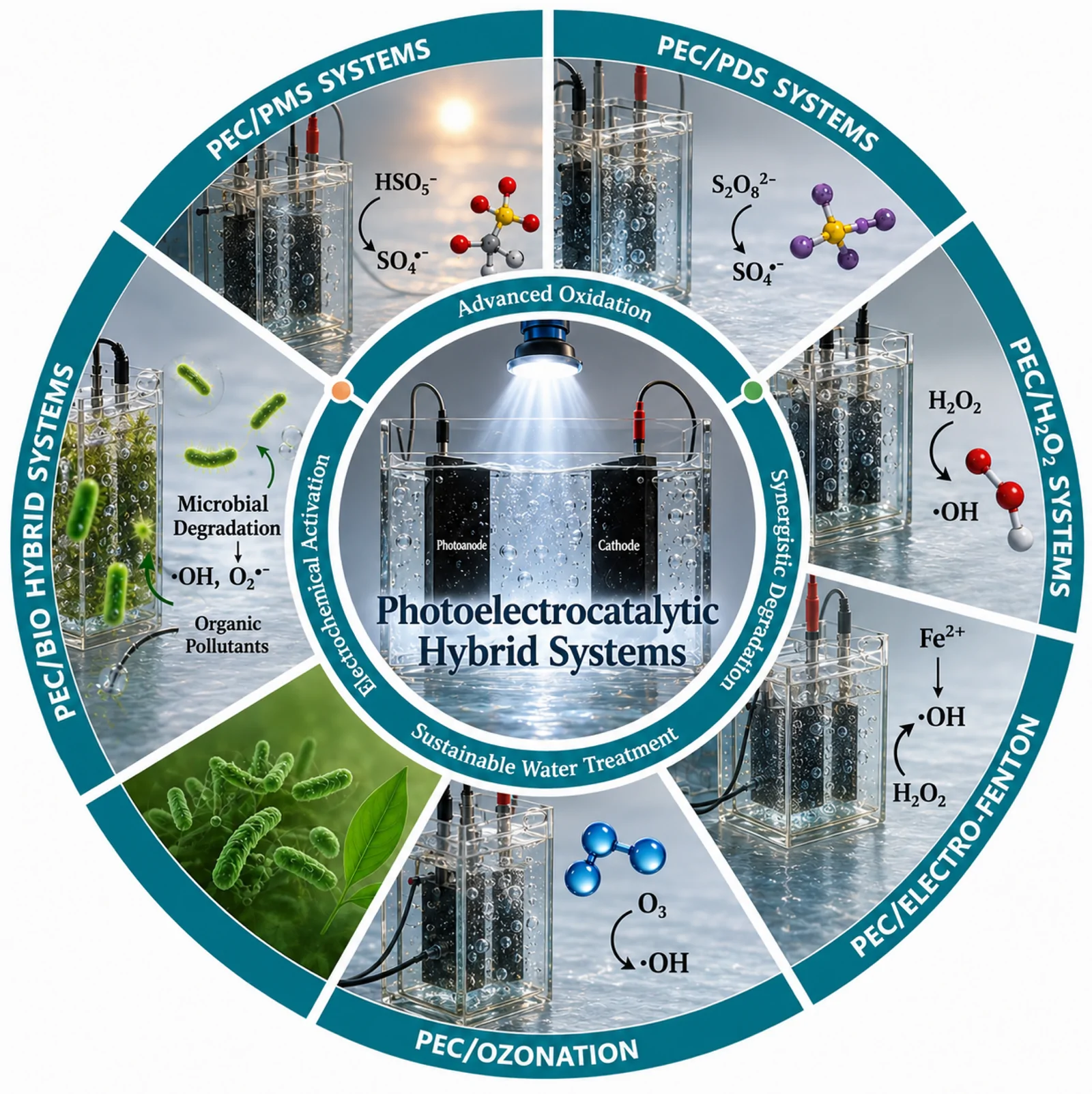
Classification of PEC hybrid systems, illustrating the integration of PEC with PMS, PDS, H_2_O_2_, electro-Fenton, ozonation, and biological processes for synergistic advanced oxidation and efficient pollutant removal.

PEC has many drawbacks under practical conditions, such as the rapid electron–hole recombination and the low generation of reactive oxygen species (ROS). To alleviate these drawbacks, hybrid PEC processes were developed by incorporating extra electron acceptors or extra radical sources, which are called auxiliary oxidants. The degradation kinetics and mineralization of these hybrids are increased by faster charge separation and higher ROS generation. For instance, the electrons generated by the photoexcitation of persulfate (PMS or PDS) can be used to produce SO_4_˙^−^ and ˙OH.^119^ The additional oxidant removes the electrons from the system, minimizing the amount of recombination, and the holes oxidize water to ˙OH. Such hybrids can produce greater ROS flux and advance the degree of oxidation of pharmaceuticals compared to standalone PEC. This section critically reviews and analyzes various hybrid PEC systems (Fig. 3). Table 2 comprehensively summarizes different PEC hybrid systems for pharmaceutical removal along with their performance metrices and reaction conditions.

**Table 2:** Summary of the previous literature reported on hybrid PEC system for pharmaceutical pollutant removal from wastewater

Hybrid system	Electrode material	Pollutant	Reaction conditions					Degradation efficiency (%)	*k* (min^−1^)	Cyclic stability (efficiency/cycle)	Predominant reactive species		Ref.
			Pollutant (mg L^−1^)	Oxidant (mM)	Applied voltage (V)	pH	Reaction time (min)				Radical	Non-radical	
PEC/PMS	Pt@CeO_2_@MoS_2_	Carbamazepine	2.0	0.76	1.0	4.0	30	75	0.13202	92/5	O_2_˙^−^, SO_4_˙^−^ ˙OH	^1^O_2_	120
PEC/PMS	FTO-Bi_2_WO_6_	Sulfamethoxazole	5.0	3.0	1.0	7.0	90	98	0.0237	93/8	—	h^+^	121
PEC/PMS	FTO-Bi_2_WO_6_	Diclofenac chloride	5.0	3.0	1.0	7.0	90	83	—	—	—	h^+^	121
PEC/PMS	FTO-Bi_2_WO_6_	Tetracycline	5.0	3.0	1.0	7.0	90	79	—	—	—	h^+^	121
PEC/PMS	BiFeO_3_/BiVO_4_	Levofloxacin	15.0	0.15	0.6	2.0	30	78	0.0447	72.5/4	SO_4_˙^−^, O_2_˙^−^, OH	h^+^	122
PEC/PMS	FeOCl/BiVO_4_	Levofloxacin	15.0	0.075	0.6	6.5	30	79	0.043	67.7/4	˙OH, O_2_˙^−^, SO_4_˙^−^	h^+^	123
PEC/PMS	1T/2H-MoS_2_@C/CC	Norfloxacin	2.0	40 ppm	1.5	6.8	25	100	—	87.6/5	˙OH, O_2_˙^−^, SO_4_˙^−^	^1^O_2_	124
PEC/PMS	TiO_2_/WO_3_	17α-ethinyl estradiol	1.0	10 ppm	1.0	—	90	88	0.044	—	SO˙^−^, ˙OH	h^+^	125
PEC/PMS	BiVO_4_/Cu_2_O	Tetracycline	20	0.12	0.6	7.0	30	91.3	0.061	—/5	O_2_˙^−^, SO_4_˙^−^, ˙OH	h^+^	126
PEC/PMS	[Mo_72_Fe_30_]/BiVO_4_	Levofloxacin	10	0.1	0.6	7.0	35	78.72	0.05909	70/5	SO_4_˙^−^, ˙OH	h^+^	127
PEC/PMS	Fe_2_O_3_–BiVO_4_	Tetracycline	20	0.1	0.6	5.7	60	82.4	0.0262	77/10	O_2_˙^−^, SO_4_˙^−^, ˙OH	^1^O_2_, h^+^	128
PEC/PMS	BiVO_4_/Co-Pi	Tetracycline	—	0.1	0.8	—	60	95.3	0.0584	87/5	O_2_˙^−^, SO_4_˙^−^, ˙OH	h^+^	129
PEC/PDS	TiO_2_NTAs/Bi_2_WO_6_/BiOI	Ofloxacin	10	1.5 g l	0.5	7.0	90	82.0	0.164	78/5	O_2_˙^−^, ˙OH, SO_4_˙^−^	h^+^	130
PEC/PDS	Self-doped TiO_2_ nanotube	Bisphenol-A	10.0 µM	1.0	1.0	7.0	60	85.9	0.0310	—	SO_4_˙^−^, ˙OH	—	131
PEC/O_3_	CN-TiNRS	Ciprofloxacin	20	215.7	1.2	6.0	90	98	—	—	O_2_˙^−^, ˙OH	h^+^	132
PEC/H_2_O_2_	Fe_3_O_4_@SiO_2_@mesoporous TiO_2_	Amoxicillin	10	2.25%	0.6	—	8.0	83.9	—	—	O_2_˙^−^, ˙OH	—	133
PEC/PDS	TiO_2_NTAs/CeO_2_/Bi_2_S_3_	Ofloxacin	10	1g l	0.6	7.0	60	77.2	0.02413	—/4	O_2_˙^−^	h^+^	134
PEC/H_2_O_2_	ZnFe_2_O_4_/TiO_2_	Tetracycline	1 mmol L^−1^	0.15%	0.5	—	90	95	0.0347	—	O_2_˙^−^, ˙OH	—	135
PEC/EF	FTO-BiVO_4_/BiOI	Paracetamol	0.1 mM	0.2	—	3.0	120	98	—	—	O_2_˙^−^, ˙OH	h^+^	136
PEC/PEF	TiO_2_/nickel foam	Carbamazepine	10.0	0.25	1.7	3.0	30	100	0.1939	—	˙OH	h^+^	137
PEC/PEF	TiO_2_/GF filter	Florfenicol	14 µM	—	0.542	—	180	99.8	—	—	O_2_˙^−^, ˙OH	^1^O_2_	138
PEC/O_3_	CN–TiO_2_	Cefadroxil	20	24 mg	1.2	6.0	90	96	—	—	˙OH	—	139
PBEC/PMS	Carbon brush	Carbamazepine	10.0	1.0	0.6	8.0	240	100	0.0221	—	SO_4_˙^−^, ˙OH	—	140
PBEC/PMS	Carbon brush	Clofibric acid	10.0	1.0	0.6	8.0	240	100	0.03665	—	SO_4_˙^−^, ˙OH	—	140
PBES	PDA/BiOBr	Sulfonamide	5.0	—	—	7.2	240	96.5	—	87.6/5	O_2_˙^−^, ˙OH	—	141

### 4.1 PEC/PMS systems

PMS, or peroxymonosulfate (HSO_5_^−^), is integrated into PEC to help overcome key PEC bottlenecks. PEC does not yield a large amount of ˙OH or superoxide (O_2_˙^−^), which can also be produced through photocatalysis or electrochemically in the presence of PMS to provide an additional source of oxidants.^142^ In the presence of excess light and bias, PMS will readily accept electrons to produce SO_4_˙^−^. In effect, PMS functions as an electron sink that extends the lifetime of charge carriers and enhances the total amount of ROS produced.^126,143^ The transition-metal centers (Co, Fe, Mn, *etc.*) or engineered vacancies further activate PMS to form both SO_4_˙^−^ and ˙OH. Importantly, hybrids of PEC/PMS can take advantage of non-radical routes as well. For instance, by carefully designed pathways of charge transfer, the surface-bound PMS can be converted to singlet oxygen (^1^O_2_).^144,145^ PEC/PMS was developed to boost charge separation and generate multiple ROS (SO_4_˙^−^, ˙OH, O_2_˙^−^, ^1^O_2_) concurrently, enhancing mineralization and reaction selectivity beyond that of simple PEC.

Under illumination, the semiconductor photoanode absorbs photons, exciting electrons from its VB to CB (reaction (1)). In a PEC/PMS hybrid, the photogenerated electrons travel to the cathode (or remain on the photoanode surface under bias) where they reduce PMS (reaction (6)). The produced SO_4_˙^−^ is a potent oxidant (*E*° ≈ 2.6–3.1 V *vs.* NHE) that can attack organic pollutants or convert to ˙OH (reaction (7)). Simultaneously, photogenerated holes oxidize water or hydroxide to ˙OH (reaction (8)). Electrons can also reduce dissolved O_2_ to superoxide (reaction (9)). These radicals may undergo further reactions *i.e.*, O_2_˙^−^ and ˙OH can combine to yield ^1^O_2_ (reaction (10)). Such non-radical pathways become significant on carbon-rich or vacancy-rich catalysts. Catalysts with carbonyl or oxygen-vacancy sites can directly transfer charge to PMS, generating ^1^O_2_ without free radicals.

6 HSO_5_^−^ + e^−^ → SO_4_˙^−^ + OH^−^

7 SO_4_˙^−^ +H_2_O → SO_4_^2−^ + ˙OH + H^+^

8 h^+^ + H_2_O → ˙OH + H^+^

9 O_2_ + e^−^ → O_2_˙^−^

10
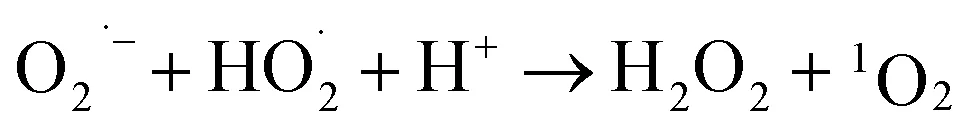

11 ^1^O_2_ + H_2_O → 3O_2_ + H_2_O

In transition-metal-assisted systems, a PMS molecule may coordinate to a metal center, and two-electron oxidation of the metal yields a high-valent metal-oxo species that oxidizes organics directly.^146^ For example, Zhang *et al.* showed that Co sites on a MoS_2_ photoanode form Co(ii)–O–O–SO_3_ complex, driving a 95.2% sulfamethoxazole conversion in just 5 min.^147^ These high-valent metal intermediates (*E*° > 1.95 V) significantly boost selectivity and degradation power beyond that of radical routes.^148^ Overall, the PEC/PMS system leverages both radical (SO_4_˙^−^, ˙OH) and nonradical (^1^O_2_, M(iv)=O) pathways photogenerated electrons activate PMS to sulfate radicals, while holes and metal centers generate ˙OH and high-valent species. The multiplicity of ROS and charge-transfer routes leads to synergistic pollutant degradation with altered kinetics and pathway selectivity relative to pure PEC.

**Fig. 4:**
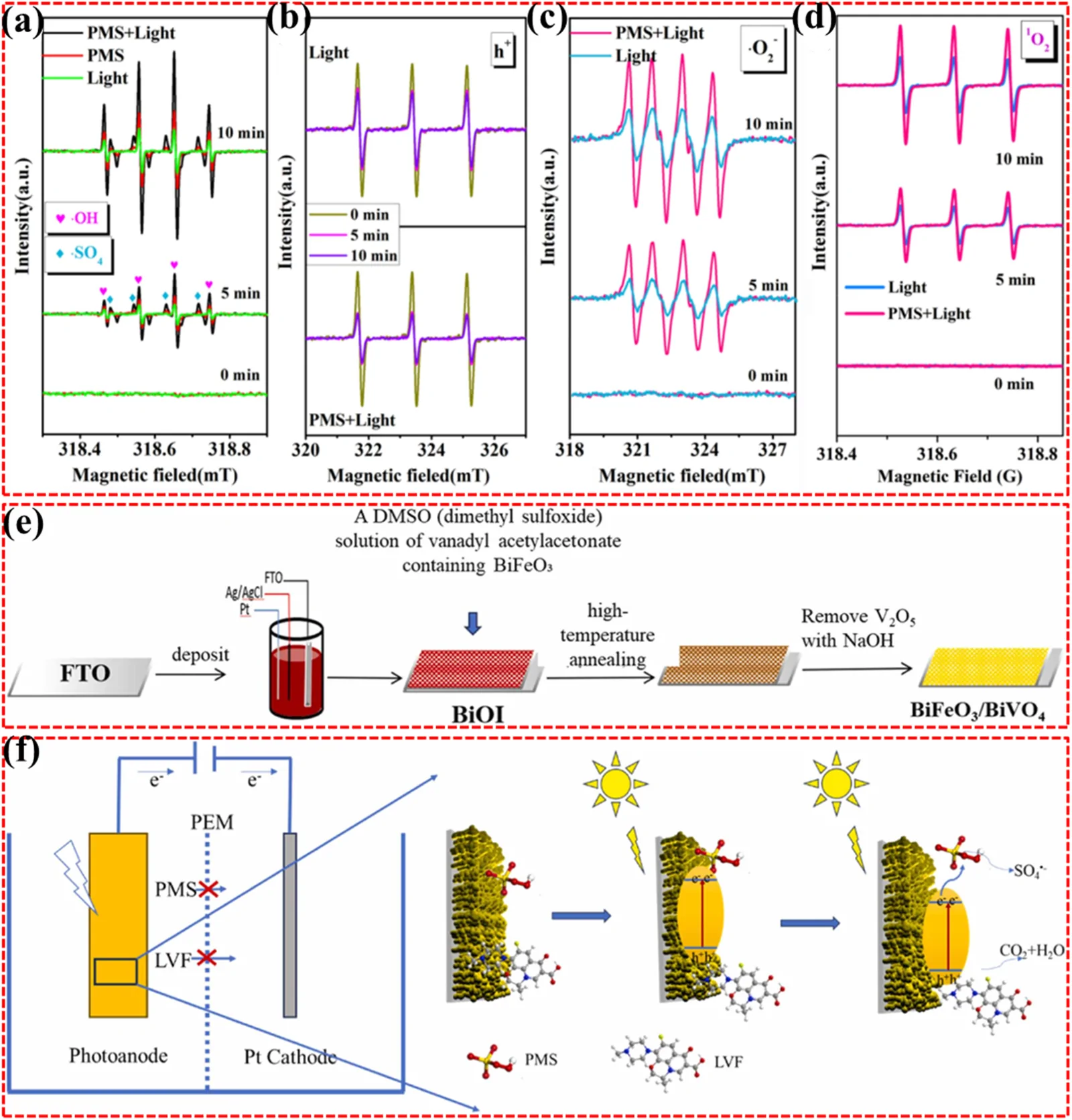
EPR spectra of BiVO_4_/Cu_2_O-30 (a) hydroxyl and sulfate group, (b) photogenerated hole, (c) superoxide radical, (d) ^1^O_2_ species. Reproduced with permission from ref. 126. Copyrights Elsevier, 2025. (e) The flow chart for preparing BiFeO_3_/BiVO_4_ composite photoanode, (f) degradation curves of LVF solution by 2-BFO/BVO photoanode in different systems. Reproduced with permission from ref. 122. Copyrights Elsevier, 2025.

For instance, Song *et al.*,^126^ constructed an S-scheme BiVO_4_/Cu_2_O heterojunction photoanode for TC degradation in a PEC/PMS system. This anode was found to be efficient in activating PMS under visible light and bias, and the switching between Cu^2+^/Cu^1+^ and electric field enhanced the PMS reduction. They suggested that the high TC degradation rate was due to the rapid separation of e^−^/h^+^ at the junction and the high concentration of SO_4_˙^−^, ˙OH, and O_2_˙^−^ radicals. The study found a significant synergic effect wherein BiVO_4_/Cu_2_O/PEC/PMS was found to be more effective than BiVO_4_/PEC, Cu_2_O/PEC, and PMS/PEC, with a TC removal of 91.48%. Both radical (SO_4_˙^−^) and nonradical (^1^O_2_) pathways were found to be active, as proven by mechanistic probes: radical quenchers and ESR (Fig. 4a–d). The S-scheme design and PMS activation resulted in a low apparent reaction time, but there was no discussion on the energy consumption or long-term stability.

In another study, Xing *et al.*^122^ prepared a BiFeO_4_/BiVO_4_ composite photoanode for the degradation of levofloxacin (LVF) under PEC/PMS (Fig. 4e). This heterojunction narrows the bandgap of FeVO_4_, Fe-site gives catalytic sites for building a heterojunction. The system successfully removed 78% of LVF in 30 min under 0.8 V bias and 0.15 mM PMS, which is much higher than the bare BiVO_4_/PEC (44%). They claimed enhanced production of SO_4_˙^−^ and ˙OH as a result of the co-catalytic Fe and BiVO_4_ sites. The authors reported a high consumption of PMS at higher voltage, which may lead to an over-consumption of oxidants. Catalytic stability was slight dropped from 77.64% to 72.55% after 4 cycles. Mechanistically, this work highlighted that the Fe^3+^/Fe^2+^ redox within BiFeO_3_ aided electron transfer to PMS, producing abundant radicals (Fig. 4f). However, like many homogeneous-metal systems, the need for acid and PMS make scale-up challenging.

**Fig. 5:**
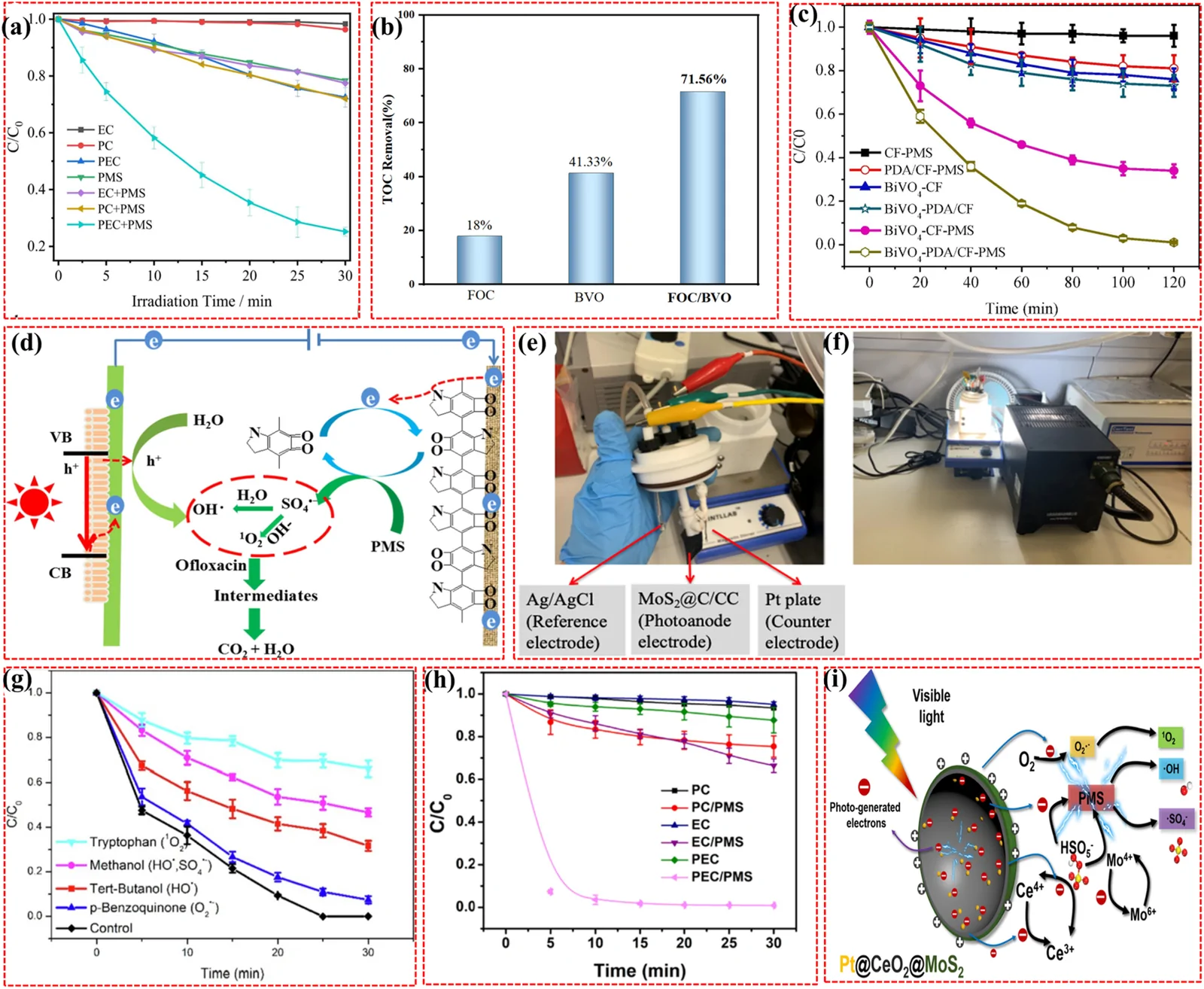
(a) Degradation curves of 15 ppm LVF solution using the 3-FOC/BVO photoanode under different systems, (b) TOC removal efficiencies of FOC, BVO, and FOC/BVO photoanodes. Reproduces from ref. 123. Which is open access and permits unrestricted use of materials under the terms of Creative Commons CC-BY. (c) Ofloxacin removal using various electrodes and in the absence/presence of PMS. Experimental conditions: [ofloxacin]_0_ = 8 mg L^−1^; applied potential = 1.5 V; pH = 5.0; [PMS]_0_ = 2 mM, (d) possible catalytic mechanism of ofloxacin degradation in the BiVO_4_-PDA/CF-PMS system. Reproduced with permission from ref. 145. Copyrights Elsevier, 2020. (e) Three-electrode configuration with MoS_2_@C/CC photoelectrode, Pt plate, and Ag/AgCl electrode serving as the photoanode, counter electrode, and reference electrode, respectively, (f) light source: 300 W Xe lamp with 420 nm cut filter, (g) scavenger test for the degradation of NOR. (Experiment condition: 1.5 V, [PMS]_0_ = 40 ppm, 1T/2H-MoS_2_@C/CC photoanode, visible light (*λ* ≧ 420 nm), [NOR]_0_ = 2 ppm, error bars stand for the standard deviations from replicates *n* = 3). Reproduced with permission from ref. 124. Copyrights Elsevier, 2023. (h) Comparison of CBZ degradation efficiency under different photoelectric conditions using Pt@CeO_2_@MoS_2_ as photoanode (experimental conditions: 20 mg catalyst, 0.76 mmol L^−1^ PMS, pH 4, light source 300 W Xe lamp with 420 nm cut filter), (i) possible mechanism illustration of Pt@CeO_2_@MoS_2_ acting as photoanode in the PEC/PMS system. Reproduced from ref. 120. Which is open access and permits unrestricted use of materials under the terms of Creative Commons CC-BY.

In the similar manner, Xing *et al.*^123^ further developed a FeOCl/BiVO_4_ photoanode for LVF in PEC/PMS. FeOCl, a layered iron oxychloride, provided both Fe^2+^ sites and unique surface structures. Under similar conditions, FeOCl/BiVO_4_-PEC/PMS removed 79% of LVF in 30 min (Fig. 5a), with 71.56% total organic carbon (TOC) reduction (Fig. 5b). Radical scavenging tests showed SO_4_˙^−^ and ˙OH as dominant oxidants. The strong performance was ascribed to FeOCl layered conductivity and PMS affinity. The system did suffer from Fe leaching and required pH 6.5 for optimal efficiency. Thus, while iron oxychloride enhanced PMS activation, acidification and iron management remain drawbacks.

In another study, Wang *et al.*^145^ reported another study using BiVO_4_ anode and polydopamine/carbon felt cathode for the PEC/PMS of ofloxacin. The system removed 100% of the ofloxacin in 120 min using 2 mM PMS (Fig. 5c). The PMS reduction by the PDA/carbon electrode yielded mainly ^1^O_2_ and not free radicals. It was demonstrated by the quenching experiments that ^1^O_2_ was the major oxidant (non-radical pathway) in the reaction, and hence ofloxacin was selectively attacked. This enhancement has been attributed to the synergy between the photogenerated electrons (that drive the PMS) and the cathode, which is rich in carbonyl groups (that promote ^1^O_2_ formation) (Fig. 5d). This work emphasizes a nonradical PEC/PMS approach where the system generated ^1^O_2_ (*E*° = 1.52 V) and was not plagued with some of the scavenging problems and delivered high efficiency in neutral or moderately acidic media. However, the use of valuable PDA and relatively low charge separation limits is not scalable.

Similarly, Zheng *et al.*^124^ synthesized a 1T/2H phase MoS_2_/C carbon-cloth hybrid material for use as the anode in PEC/PMS for norfloxacin degradation (Fig. 5e and f). The fast electron transfer to PMS in the material with high conductivity resulted in a mechanism dominated by ^1^O_2_, HO˙, SO_4_˙^−^. They found that complete mineralization of norfloxacin was achieved under 1.5 V bias in 30 min for the addition of PMS 40 ppm. The dual-phase MoS_2_ offered enough active sites, while also simultaneously enabling O_2_ activation. The analysis of the study indicated that the pathway by which PMS was activated on MoS_2_ surfaces to produce ^1^O_2_ was unusual, as it was not *via* free radicals. This nonradical pathway resulted in more rapid kinetics and almost complete mineralization. The downside is that MoS_2_ is expensive, and the highly reductive bias (5 V) creates energy issues. On the downside, MoS_2_ is costly and the highly reductive bias (5 V) raises energy concerns. Mechanistic investigations demonstrated that ^1^O_2_, ˙OH, SO_4_˙^−^ and O_2_˙^−^ contributed to norfloxacin degradation (Fig. 5g), and the PMS activation pathways for the generation of these reactive species were also revealed. The intermediates of NOR degradation by the PEC/PMS system were analyzed, suggesting that the C–N bond, piperazine ring, and fluoride group on NOR molecular framework were attacked. The resistance of the PEC/PMS system to different water matrices and good reusability (87.6% at the fifth cycle) and stability (0.05 mg per L Mo^4+^ leaching) of the 1T/2H-MoS_2_@C/CC photoanode ensured the excellent practical potential of the developed PEC/PMS system.

Dong *et al.*^120^ prepared a hollow Pt@CeO_2_@MoS_2_ ternary photoanode for carbamazepine (CBZ) removal *via* PEC/PMS. The design combined co-catalytic metal (Pt) and redox-active support (CeO_2_) on a MoS_2_ frame. They achieved 85% CBZ removal in 30 min (0.5 V bias, 0.76 mmol per L PMS) (Fig. 5h). Mechanistic studies indicated dual paths *i.e.*, Pt sites accelerated electron transfer and ˙OH production, while Ce^3+^/Ce^4+^ redox aided PMS activation (Fig. 5i). Light absorption and separation of charges was increased by a hollow structure. This system was very effective but had complexity and cost (Pt) problems. Stability was good (92% after 5 cycles), and the regeneration of the catalyst was not easy.

In all these studies the PEC/PMS systems achieve much greater degradation rates than PEC alone. Mechanistically, engineering charge-transfer, heterojunctions (*e.g.* BiVO_4_/Cu_2_O) and dopants (Co, Mo) are shown to be very effective for the extraction of electrons to PMS. Non-radical ^1^O_2_ pathways have proven to be promising and are able to selectively oxidize with fewer losses by scavenging. However, excessive PMS use may pose toxicity concerns, while high applied bias and strongly acidic conditions can increase energy consumption and operational costs.^149,150^ Stability is mixed, many catalysts have moderate deactivation after a few cycles, typically because of metal leaching or fouling of the surface by intermediates. Since bias and auxiliary chemicals in most energy efficiencies, are generally lower than PEC alone.

PEC/PMS has several benefits over other PEC hybrid systems. The SO_4_˙^−^ and the associated ˙OH are extremely strong and have rather long lifespans,^151^ and can therefore oxidize recalcitrant pharmaceuticals even in the presence of natural water matrices. The ˙OH generated from H_2_O_2_, however, are of shorter lifetime and are easily scavenged by bicarbonate or natural organic matter.^152^ Furthermore, carbonaceous or vacancy-rich catalysts can produce ^1^O_2_ in PEC/PMS systems, which is a selective, greener, and less radical pathway to degrade organics. There are also limitations to PEC/PMS. Limiting aspects include the cost and handling of PMS and the toxic residues that might remain in effluent if PMS is not completely activated.^150^ Normal pH levels are not suitable for real-water application for the process. Energy requirements for bias (typically 2–5 V) are similar to other PEC hybrids, with some extra energy required for PMS recycling. Finally, the design of catalysts which do not leach out metal (Fe, Co) and remain active is an important research challenge. Overall, PEC/PMS has high kinetic rates and ROS diversity, but the process needs to be practically deployed considering the control of oxidant usage and catalyst stability.

### 4.2 PEC/PDS systems

Peroxydisulfate (PDS, S_2_O_8_^2−^) is a close analog to PMS but offers distinct benefits and challenges. Like PMS, PDS produces SO_2_˙^−^ upon activation, but it lacks the extra oxygen atom, leading to no direct HO˙ from the parent molecule.^153^ Hence, PEC/PDS demands different activation energy for the breaking of the S–O bond in S_2_O_8_^2−^, either by changing the surface of the catalyst (*e.g.*, oxygen vacancies, dopants) or by raising the bias. However, the oxidation state of PDS (S–O bond) is higher, and it can produce 2SO_4_˙^−^/molecule.^154^ This usually results in an increase in the steady-state SO_4_ concentration and the presence of more persistent radicals. PEC/PDS hybrids were designed to take advantage of these properties to produce SO_4_˙^−^ selectively and to increase the lifetime of ROS and stability against scavenging. In practice, the initial activation kinetics of PDS-PEC are generally slower than those of PMS-PEC, but the resulting mineralization is comparable or better in the long-term due to the robustness of SO_4_˙^−^.

PEC/PDS is similar to PEC/PMS but has some distinct differences. In the presence of light and bias, the electrons will reduce PDS (reaction (12)) to produce SO_4_˙^−^. The ˙OH is produced by reaction (8) with photo-holes and water. Under PEC conditions, complete two-electron reduction of PDS instead yields two sulfate ions without radical character (reaction (13)), a competing electron sink rather than a second radical-generating route, which is why single-electron activation (reaction (12)) dominates SO_4_˙^−^ production in practice. Electrons can also reduce O_2_ to O_2_˙^−^, leading to downstream 
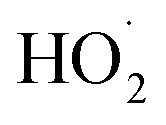
 and H_2_O_2_. Generally, the net ROS pool in PEC/PDS contains SO_4_˙^−^, ˙OH, O_2_˙^−^, and maybe ^1^O_2_ through the recombination of O_2_˙^−^. It is important to note that ^1^O_2_ is not produced directly from PDS (without intermediates) as in PEC/PMS. Again, non-radical routes are possible with transition-metal catalysts, such as the capability of Cu^3+^ species to be formed by PDS, which in turn can oxidize organics.

12 S_2_O_8_^2**−**^ + e^−^ → SO_4_˙^−^ + SO_4_^2−^

13 S_2_O_8_^2−^ + 2e^−^ + 2 SO_4_^2−^

Electron transfer is a more significant requirement for the activation of PDS than in the case of PMS. The adsorption on catalysts and the cleavage by e^−^ of PEC processes are often stressed. Persulfate systems enable a switch from radical to non-radical pathways using the choice of catalyst.^155^ For instance, carbon catalysts favor ^1^O_2_, whereas metal oxides favor SO_4_˙^−^ production.^156,157^ PEC/PDS generates a sulfate-radical-rich environment similar to PEC/PMS, but with slightly slower initiation and greater emphasis on electron-driven activation.

**Fig. 6:**
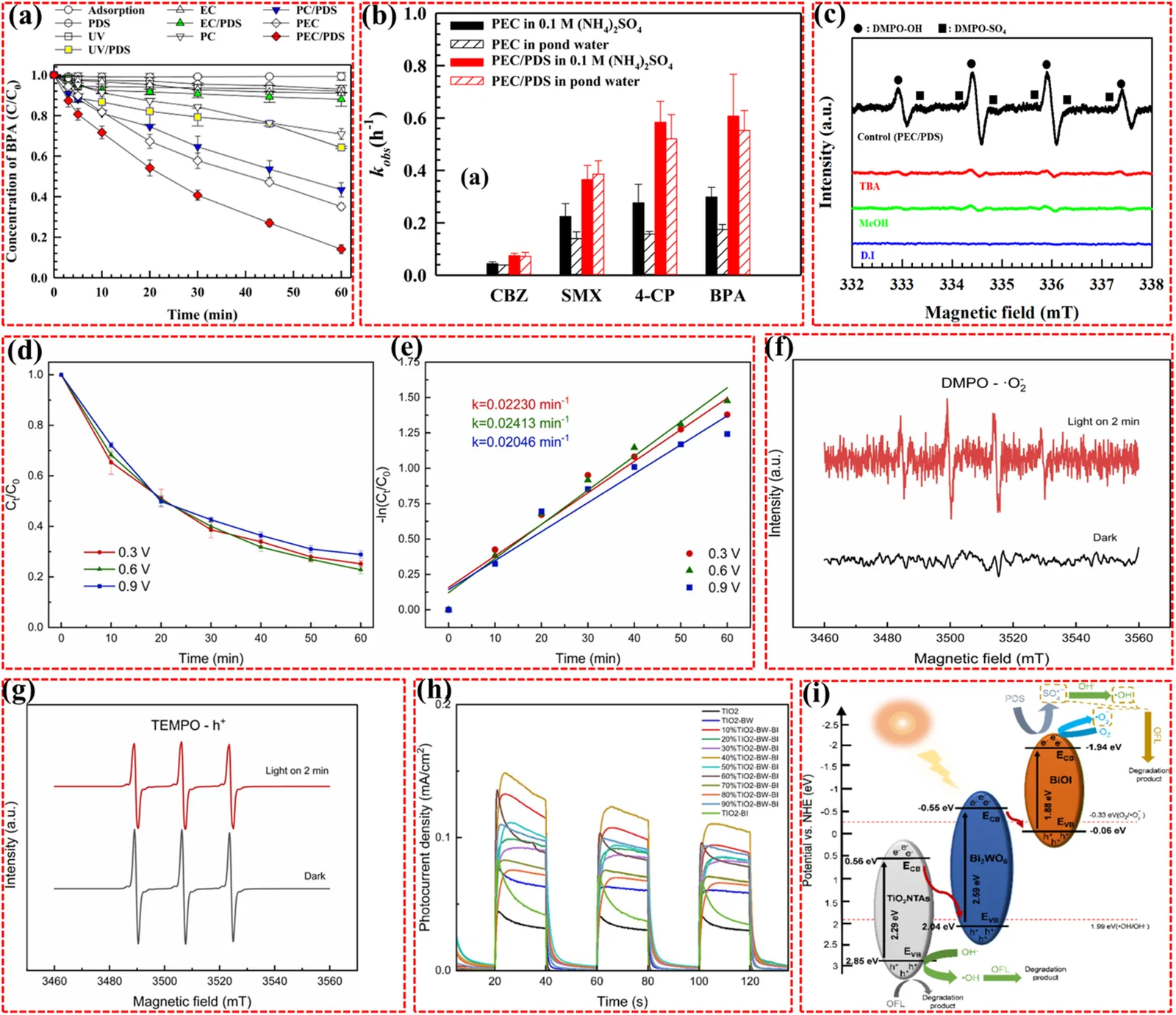
(a) Removal efficiency of BPA using bl-TNAs under various operating conditions (photolysis, electrocatalysis [EC], photocatalysis [PC], and photoelectrocatalysis [PEC] with and without peroxydisulfate [PDS]. Conditions: [BPA]_0_ = 10.0 µM; [PDS]_0_ = 1.0 mM; [(NH_4_)_2_SO_4_]_0_ = 0.1 M; potential bias = 1.0 V *vs.* Ag/AgCl; pH_i_ = 5.5), (b) ESR spectra of photoelectrocatalysis (PEC)/peroxydisulfate (PDS) with and without radical scavengers ([(NH_4_)_2_SO_4_]_0_ = 0.1 M; [PDS]_0_ = 1.0 mM; [TBA]_0_ = [MeOH]_0_ = 1.0 M; potential bias = 1.0 V *vs.* Ag/AgCl; pHi = 5.5), (c) ESR spectra of photoelectrocatalysis (PEC)/peroxydisulfate (PDS) with and without radical scavengers ([(NH_4_)_2_SO_4_]_0_ = 0.1 M; [PDS]_0_ = 1.0 mM; [TBA]_0_ = [MeOH]_0_ = 1.0 M; potential bias = 1.0 V *vs.* Ag/AgCl; pH_*i*_ = 5.5). Reproduced with permission from ref. 131. Copyrights Elsevier, 2021. Effect of different applied potentials on (d) degradation rate of OFL and (e) the pseudo-first-order kinetics. ESR spectra of (f) DMPO-O_2_˙^−^ and (g) TEMP-h^+^. Reproduced with permission from ref. 134. Copyrights Elsevier, 2025. (h) Comparison of transient photocurrents for different electrodes, (i) putative mechanism diagram for fast and efficient degradation of OFL in TiO_2_NTAs/Bi_2_WO_6_/BiOI/visible light/PDS system. Reproduced with permission from ref. 130. Copyrights Elsevier, 2026.

Son *et al.*^131^ reported that the incorporation of PDS into a black TiO_2_ nanotube array-based PEC system markedly enhanced BPA degradation efficiency from 65.0% in the conventional PEC process to 85.9% in the PEC/PDS system within 60 min (Fig. 6a). The pseudo-first-order kinetic constant in surface water was nearly three times higher for PEC/PDS than PEC alone, demonstrating the strong synergistic effect of persulfate activation. Further, the degradation performance of the organic pollutant mixture, including BPA, 4-chlorophenol (4-CP), sulfamethoxazole (SMX), and carbamazepine (CBZ), was evaluated in 0.1 M (NH_4_)_2_SO_4_ solution and real surface water. The degradation efficiency increased in the order of CBZ < SMX < 4-CP < BPA in the PEC and PEC/PDS systems with both water matrices. Compared with the PEC system, the PEC/PDS (1.0 mM) system showed a threefold higher pseudo first-order reaction rate constant for BPA among pollutant mixtures in surface water (Fig. 6b). This improvement is attributed mainly to the better photocatalytic charge separation, faster interfacial electron transfer, and generation of the highly reactive ˙OH and SO_4_˙^−^ radicals simultaneously. Specifically, SO_4_˙^−^ could provide selective and long-term oxidation capacity, and PDS was also an electron acceptor that inhibits electron–hole recombination (Fig. 6c), which greatly enhanced the oxidative degradation performance in complex water matrices.

Yuan *et al.*^134^ fabricated a double Z-scheme TiO_2_NTAs/CeO_2_/Bi_2_S_3_ heterojunction for the PEC degradation of ofloxacin (OFL) in the presence of PDS. Fig. 6d and e show the degradation of OFL using the TiO_2_NTAs/CeO_2_/Bi_2_S_3_/visible light/PDS system, which obtained 77.2% degradation after 60 min. The synergistic effect of the double Z-scheme heterojunction and PDS activation was considered as the reason for the improvement of performance. Incorporation of CeO_2_ and Bi_2_S_3_ increased the visible-light absorption and enhanced charge separation and transfer efficiency and decreased interfacial resistance. Moreover, the photoelectrocatalyst was used for the effective activation of PDS, resulting in the formation of ROS, mainly h^+^ and O_2_˙^−^, for the enhancement of OFL degradation (Fig. 6f and g). The applied bias further reduced the electron–hole recombination, which resulted in an increase in PEC activity. The results show that a system consisting of engineered Z-scheme photoelectrocatalysts and PDS is a promising approach to enhance the degradation of persistent antibiotic contaminants.

In a similar study Yuan *et al.*^130^ fabricated a double Z-scheme TiO_2_NTAs/Bi_2_WO_6_/BiOI heterojunction with a PDS-assisted PEC system for OFL degradation in a similar study. The ternary heterojunction showed significantly higher photocurrent density of 0.695 mA cm^−2^ as shown in Fig. 6h, which is almost 16 times higher than the pristine TiO_2_NTAs, due to enhanced visible light harvesting and efficient charge separation. The PEC/PDS system reached 82% OFL degradation with a reaction rate constant of 3.5 times higher than the conventional PEC process under optimal conditions in 90 min. The double Z-scheme architecture (Fig. 6i) was responsible for enhanced activity, which enabled charge carrier migration without losing redox capability, which was important for PDS activation and generation of reactive species. Mechanistic studies showed that h^+^ and O_2_˙^−^ were the major oxidative species, with a contribution from ˙OH and SO_4_˙^−^ to a lesser extent. The results of this study show that the combination of Z-scheme heterojunctions and PDS-assisted PEC processes could significantly enhance the degradation efficiency of recalcitrant antibiotic pollutants.

Overall, these studies indicate that the same trends are observed in PEC/PDS: pharmaceutical degradation rates increase significantly when PDS is added (often 2–3× increase in *k*).^16^ Again, the main ROS are SO_4_˙^−^ and ˙OH, and O_2_˙^−^/^1^O_2_ in minor roles in some instances. Generally, PDS systems require a higher dose of oxidant as compared to PEC/PMS systems, and PDS systems are also biased due to the higher S–O bond strength. Many authors report that PDS results in a higher amount of TOC removed (mineralization) but at the expense of a lower rate of initial mineralization, consistent with a longer lifetime of sulfate radicals. To overcome the slower activation kinetics, catalysts with innovations (heterojunctions, dopants, vacancies) are used. In most studies, stability is generally satisfactory as the activation of PDS generally involves surface catalysis (which is less corrosive than PMS).

PEC/PDS has its advantages and disadvantages compared to other hybrid schemes. Its benefits include no easy decomposition in dark conditions which gives it the ability to be activated by a controlled electrode. Molecules such as SO_4_˙^−^ that are produced have a longer lifetime than the ˙OH and can thus undergo multiple electron transfer reactions. Therefore, PEC/PDS can attain greater mineralization and can tolerate some background scavengers. In addition, PDS systems can function at a wide range of pH levels persulfate and sulfate radicals are less pH sensitive than H_2_O_2_ or Fenton catalysts.

The drawback is that higher applied voltage or catalyst modification (defects, metals) may be required to obtain PEC/PDS, and this can increase energy input. Also, since PDS is devoid of –OH, there will be lower yields of initial ˙OH until some of this SO_4_˙^−^ undergoes conversion to ˙OH. Also, if there are radical quenching reactions (excess PDS consuming SO_4_˙^−^), the efficiency will decline, so careful dose control is required. Overall, the PEC/PDS systems are more selective for mineralization of sulfates and faster as a result but have slightly higher energy requirements and slower speeds than the PEC/PMS systems. They offer good stability and selectivity, which is crucial for the complete degradation of pollutants; however, they require more advanced catalysts and process optimization to overcome the slower activation barrier.

### 4.3 PEC/H_2_O_2_ systems

In the PEC framework, a classic Fenton oxidant, hydrogen peroxide (H_2_O_2_), is incorporated. There are two ways to cathodically produce H_2_O_2_*in situ* either by a two-electron oxygen reduction or by external dosing.^158^ In the hybrid, H_2_O_2_ acts as an electron acceptor, preventing charge recombination and producing more ˙OH radicals.^159^ This integration evolved to make use of the high oxidation potential of ˙OH and the well-established electron-sinking effect of H_2_O_2_. The typical PEC/H_2_O_2_ system uses O_2_ as the cathode reactant, which is reduced to H_2_O_2_ (reaction (14)).^160^ In the meantime, the photogenerated holes on the photoanode oxidize water to ˙OH as is typical.^161^ The *in situ* H_2_O_2_ can then undergo reactions with electrons and/or metal sites to generate more ˙OH (reaction (15)) or with photo-Fenton if there is trace iron. The ultimate effect is an increase in the steady state [˙OH] and electron lifetime.

14 O_2_ + 2H^+^ → H_2_O_2_

15 H_2_O_2_ + e^−^ → ˙OH + OH^−^

However, H_2_O_2_-based hybrids also entail new dynamics. Compared to SO_4_˙^−^, ˙OH (*E*° ≈ 1.9 V) is less selective and can be easily scavenged by water matrix components such as bicarbonate and chloride.^162–164^ H_2_O_2_ is not stable and may break down into O_2_ and water, particularly when the pH is high or the temperature is elevated (reaction (16)). It can also react with ˙OH *via* self-scavenging (reaction (17)). Optimization of PEC/H_2_O_2_ balances the production of H_2_O_2_ and its loss. In general, such systems work best in a somewhat acidic range of pH (3–5) to stabilize H_2_O_2_ and to improve ORR. Furthermore, the reactive spectrum in H_2_O_2_ is narrower because it produces mainly ˙OH (and a small amount of O_2_˙^−^ from 
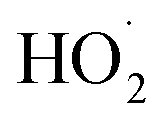
 disproportionation). However, the combination of the cathodic production of hydrogen peroxide (H_2_O_2_) and the photogenerated holes frequently enables quicker kinetics than PEC alone, and can be accompanied by the absence of additional oxidants other than O_2_.^165^

16 2H_2_O_2_ → 2H_2_O + O_2_

17
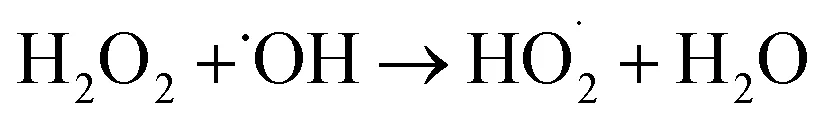


The crucial step in PEC/H_2_O_2_ systems is the photoanode coupling with the two-electron reduction of oxygen at the cathode. On some cathodic materials, such as carbon, noble metals, and even BiVO_4_ under some circumstances, electrons are reduced to H_2_O_2_ (reaction (14)). The H_2_O_2_ formed diffuses and can directly oxidize organics (less probable) or is activated by electrons to generate ˙OH or by photogenerated holes by a photo-Fenton-type pathway. At the same time, the holes existing on the photoanode still oxidize water. In other words, H_2_O_2_ provides a new electron transfer pathway, it inhibits the recombination of the electrons, and the electrons go to the pathway of radical generation.^165,166^ Combining the two half-reactions, the net pathway from O_2_ to ˙OH combines the cathodic two-electron reduction of O_2_ to H_2_O_2_ (reaction (14)) with the subsequent photolytic O–O bond homolysis of H_2_O_2_, summarized in one-step notation as reaction (18), corresponding to the sequential O_2_ → H_2_O_2_ → 2˙OH process. This translates into a much improved ˙OH flux. Under standard operating conditions, the electron-driven route is more significant, but if the cathode is photoactive, visible-light-driven photolysis of H_2_O_2_ may also play a role.

18 O_2_ + 2H^+^ + 2e^−^ + *hv* → 2˙OH

Note that it is different from PMS/PDS in that the H_2_O_2_ cannot generate SO_4_˙^−^ or ^1^O_2_ (except through secondary reactions). The dominant ROS in PEC/H_2_O_2_ systems are ˙OH and minor quantities of O_2_˙^−^ arising from 
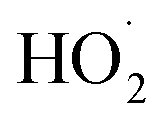
 intermediates.^167^ The pathway of ˙OH generation is still ongoing and hole oxidation of organics is still active. Some designs include Fe^2+^ (Fenton reagent) to accelerate the process of activation of H_2_O_2_, which becomes a PEC/photo-Fenton hybrid.

**Fig. 7:**
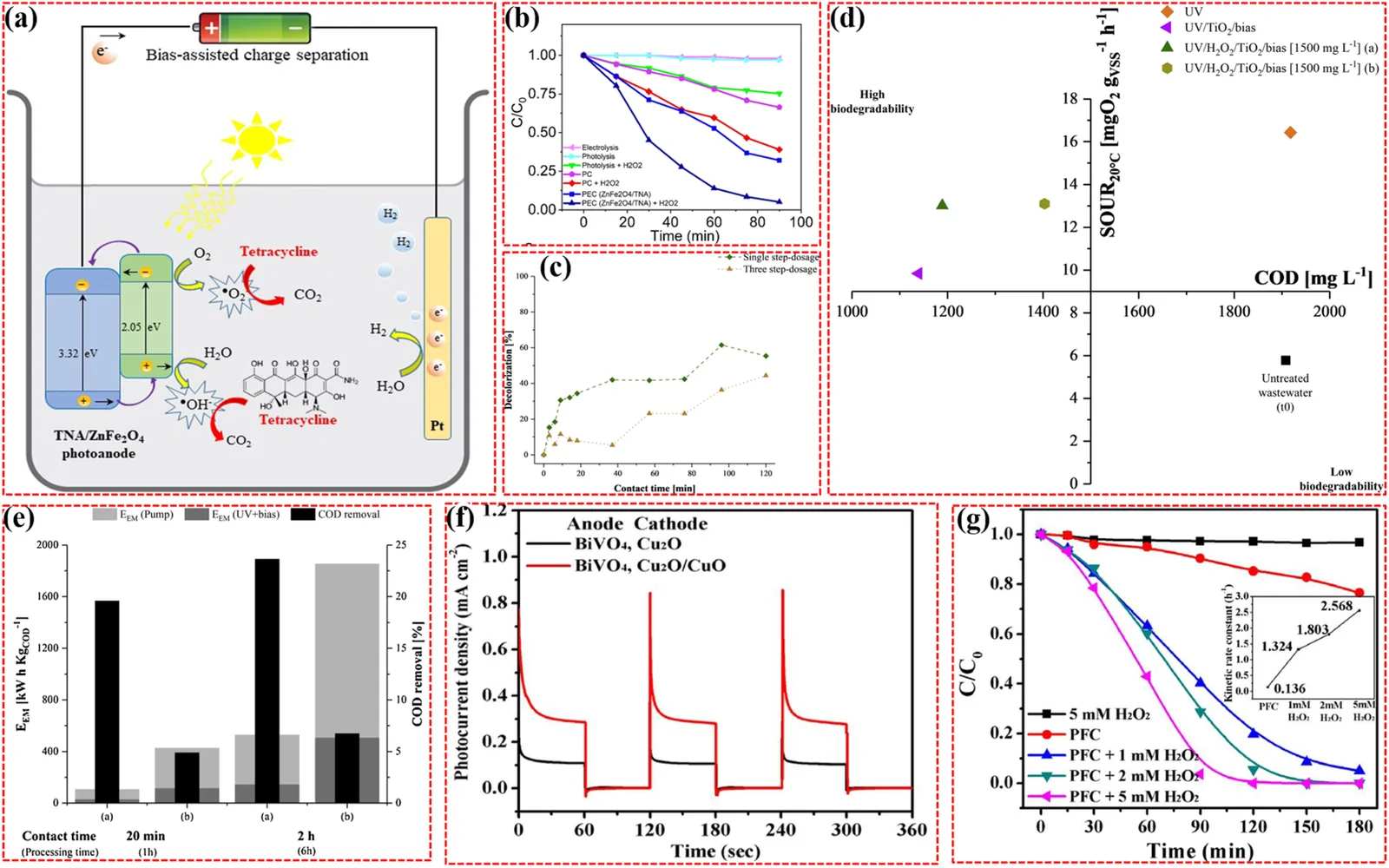
(a) Schematic illustration of tetracycline degradation, (b) comparison of the degradation of TC in various systems. Reproduced from ref. 135. Which is open access and permits unrestricted use of materials under the terms of Creative Commons CC-BY. (c) Relative percentage decolorization of the IWW during H_2_O_2_-assisted PEC (H_2_O_2_ dosage = 1500 mg L^−1^), (d) SOUR values after 2 h of H_2_O_2_-assisted PEC processes ((a) single-step and (b) three-step H_2_O_2_ dosage) and comparison with SOUR values after 2 h of PEC and UV, (e) values of electrical energy per unit of mass (*E*_EM_ kW h kg^−1^) after 20 min and 2 h (contact time) of UV/H_2_O_2_/TiO_2_/bias process with (a) single-step dosage and (b) three-steps dosage of H_2_O_2_ in comparison with COD removal efficiency. Reproduced with permission from ref. 168. Copyrights Elsevier, 2020. (f) The effect of CuO/Cu_2_O heterojunction on the photocurrent densities of the PFC systems, where the substrate is 0.05 M phenol and support electrolyte is 0.1 M Na_2_SO_4_, (g) effect of H_2_O_2_ concentration on phenol removal and corresponding kinetic constants (inset). Reproduced with permission from ref. 169. Copyrights Elsevier, 2018.

For instance, to degrade tetracycline, a nanotube photoanode of TiO_2_ decorated with ZnFe_2_O_4_ was developed by Sheikhpour *et al.*^135^ The cathodic reduction of O_2_ was followed by the formation of H_2_O_2_ in their system, which then reacted to yield ˙OH radicals (Fig. 7a). They demonstrated that the efficiency of TC removal could reach 95% in 90 min with 0.15% H_2_O_2_ added to the electrolyte (at 1.5 V bias under visible light) (Fig. 7b). This enhancement was due to two factors: electron sinks (H_2_O_2_), which reduced the recombination of the e^−^ and h^+^ and the possibility of reaction (14). The ZnFe_2_O_4_ sites also promoted the decomposition of H_2_O_2_ to ˙OH, which is a heterogeneous Fenton-like component. Modestly higher energy consumption was observed as compared to PEC, demonstrating that H_2_O_2_ doping can be energy efficient when appropriately designed.

Collivignarelli *et al.*^168^ studied the treatment of real pharmaceutical industrial wastewater using TiO_2_ mesh electrodes produced by plasma electrolytic oxidation (PEC), H_2_O_2_-assisted PEC, photolysis, and H_2_O_2_-assisted photolysis processes. The highest decolorization efficiency (55%) and COD removal (24%) were observed after 2 h with the UV/H_2_O_2_/TiO_2_/Bias system after 2 h, indicating the synergistic effect of UV/PEC and activation by H_2_O_2_ (Fig. 7c). This improvement is likely due to the production of more ˙OH radicals from the photolysis of H_2_O_2_ and the subsequent reaction with the photogenerated electrons, which would reduce electron–hole recombination and allow for more oxidative species to be available. The applied bias also enhanced the charge separation, which resulted in better oxidation of pollutants than with any UV or UV/H_2_O_2_ alone processes. Notably, PEC treatments also improved the wastewater biodegradability as seen by higher SOUR values, whereby some of the recalcitrant substances were partially converted to more biodegradable intermediates. Although decoloration and COD removal were observed, the relatively low efficiency of the decoloration (Fig. 7d) and COD removal indicates that the complex matrix of industrial wastewater will still be difficult to mineralize completely. The study shows, however, that the combination of H_2_O_2_-assisted PEC can be applied effectively to enhance the biodegradability and treatment of pharmaceutical wastewater, while preserving energy efficiency (Fig. 7e).

A self-driven photocatalytic fuel cell (PFC) was developed by Li *et al.*^169^ to degrade phenol, consisting of a BiVO_4_ photoanode and a photo-cathode based on a noble metal-free layer of Cu_2_O/CuO. The BiVO_4_–Cu_2_O/CuO system performed better than the BiVO_4_–Pt and the BiVO_4_–Cu_2_O systems, which was attributed to the favorable Fermi level alignments between the photoelectrodes, creating a larger internal bias and improving charge separation and electron transport. Thus, the photocurrent density increased to 0.30 mA cm^−2^ (Fig. 7f) and the photocatalytic activity was enhanced. Addition of H_2_O_2_ greatly enhanced the degradation of phenol with a pseudo-first-order rate constantly as high as 2.568 h^−1^ (Fig. 7g) as compared to 0.136 h^−1^ in the absence of H_2_O_2_. The dual activation of H_2_O_2_ by the photogenerated electrons and the Cu_2_O/CuO photocathode was enhanced, such enhancement by Cu(i)/Cu(ii) redox cycle, which induced Fenton-like reactions, and the continuous generation of the reactive oxygen species. The obtained results illustrate the superior efficiency of pollutant degradation by combining H_2_O_2_ activation to self-driven PEC systems, besides overcoming some drawbacks of the conventional Fenton technologies.

In the PEC/H_2_O_2_ studies, there are common elements. The reaction rate is dramatically increased by the presence of H_2_O_2_, sometimes doubling or tripling the rate over bare PEC. However, the gains level off at high H_2_O_2_ self-scavenging and parasitic decomposition impose an optimal concentration. Many experiments use acidic pH (3–5) as this is where H_2_O_2_ is most stable, but neutral operation is feasible for an even slower reaction rate. The focus of electrode materials is the promotion of 2e^−^ ORR (to produce H_2_O_2_) and activation of this species.^170^ The durability is generally good as H_2_O_2_ does not corrode stable semiconductors, unlike the case with metal leaching in EF. However, the instability of H_2_O_2_ (slow degradation in water) and the small window of active ROS (mainly ˙OH) may constrain the effectiveness in complex waters.^171^ PEC/H_2_O_2_ is non-oxidative, and selectivity is lower compared to sulfate-based systems.

The hybrids PEC/H_2_O_2_ are valued for their ability to produce the highest number of ˙OH. This results in the fast degradation of antibiotics susceptible to hydroxyl attack. It is more non-selective and rapidly quenched by components of the matrix (bicarbonate, organic matter), which is a drawback. PEC/H_2_O_2_ is often more effective than PEC alone but is not as fast as SO_4_˙^−^ or ozone. Energy-economically, the PEC/H_2_O_2_ system is not too expensive because the H_2_O_2_ can be electrogenerated rather than fed, but the overall cell voltage is comparable to other systems. The drawbacks are a very limited pH range (outside of the acidic regime), a dramatic drop in H_2_O_2_ production, and the system is essentially PEC only. Furthermore, too high a concentration of H_2_O_2_ can serve as a radical scavenger, and optimization of dosing is critical. For pharmaceutical wastewater, there are no pathways of high-valent or singlet oxygen, which could leave behind more biodegradable intermediates. In this sense, PEC/H_2_O_2_ is a powerful yet less precise oxidation system with limited working parameters and can be self-inhibited.

### 4.4 PEC/electro-Fenton

The PEC/Electro-Fenton (PEC/EF) hybrid merges PEC with classic electro-Fenton chemistry. In electro-Fenton, H_2_O_2_ is produced *in situ* at a cathode, and Fe^2+^ (either added or released from a sacrificial anode) catalyzes its conversion to ˙OH *via* the Fenton reaction.^158,172^ In PEC/EF, the photoanode provides holes and photons to assist these processes, while the cathode produces H_2_O_2_ and Fe cycles between Fe^2+^/Fe^3+^.^173^ This system developed to intensify the Fenton process by leveraging photogenerated charge separation and avoiding continuous chemical addition. The net effect is a highly potent oxidizing system photogenerated holes and ˙OH (from Fenton) attack pollutants in tandem. Importantly, PEC provides the electric field and light to regenerate Fe^2+^ from Fe^3+^ photochemically (photo-Fenton effect), maintaining the catalytic cycle.^174,175^ Homogeneous PEC/EF uses dissolved Fe^2+^ and two chambers (anode/cathode). Heterogeneous variants immobilize iron on cathodes or membranes to avoid sludge.

Mechanistically, the cathode in PEC/EF is typically a carbon or PTFE-based gas-diffusion electrode. Electrons reduce O_2_ to H_2_O_2_ (reaction (14)). Meanwhile, an iron anode (sacrificial) can release Fe^2+^, or Fe^2+^ is added externally. At acidic pH, Fe^2+^ reacts with H_2_O_2_ in solution *via* the classical Fenton reaction (reaction (19)). Photogenerated holes (on the photoanode) and UV/visible light accelerate Fe^3+^ reduction back to Fe^2+^*via* the photo-Fenton pathway (reaction (20)), which regenerates Fe^2+^ and produces additional ˙OH, essentially recycling the iron catalyst.^176^ Direct hole oxidation takes place at the photoanode, while the dominant ROS is the Fenton product, ˙OH, with some Fe(iv) species also being formed.^177^ Obviously, the synergy between the photoanode and the electro-Fenton is seen in the generation of a higher flux of ˙OH as compared to PEC alone or electro-Fenton alone.

19 Fe^2+^ + H_2_O_2_ → Fe^3+^ + ˙OH + OH^−^

20 Fe^3+^ + H_2_O + *hν* → Fe^2+^ + ˙OH + H^+^

**Fig. 8:**
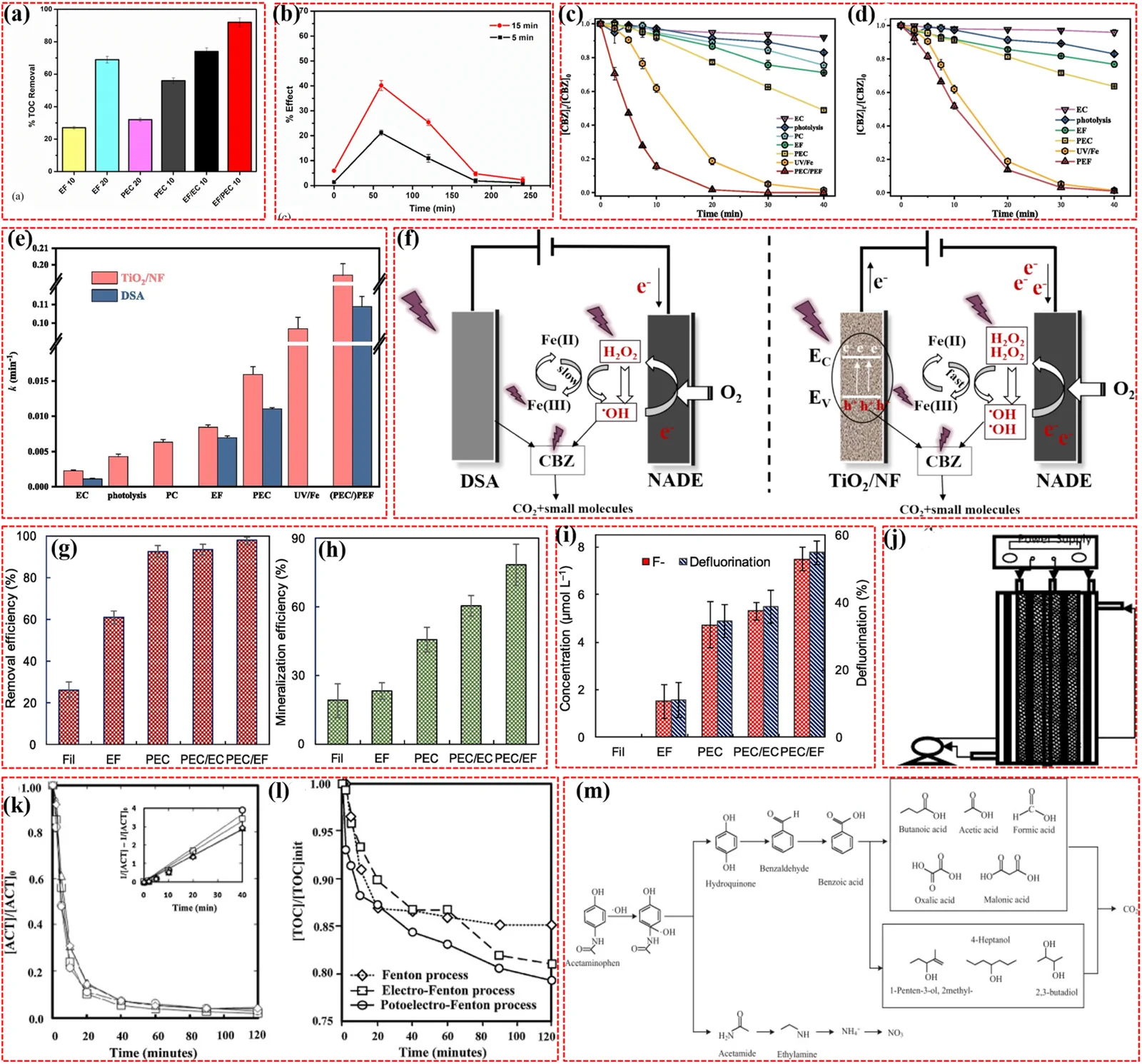
(a) Comparison between percentage TOC removal of paracetamol through coupled process of electro-Fenton/PEC degradation, electro-Fenton and PEC process at different current densities, (b) toxicity assessment for the coupled process. RSD (*n* = 5): 12% if % effect < 20%; 5% if % effect is 20–70% and 0% if % effect > 70%. (0.1 mM paracetamol; 50 mM Na_2_SO_4_; pH 3; inner electrode distance 3 cm). Reproduced with permission from ref. 136. Copyrights Elsevier, 2020. The removal of CBZ by different systems based on (c) TiO_2_/NF and (d) DSA, (e) comparison of the pseudo-first-order rate constants of CBZ removal in different systems. Experimental conditions: initial CBZ concentration = 10 mg L^−1^, initial pH = 3.0. If the following parameters are involved: Fe^2+^ dosage = 0.5 mM, for TiO_2_/NF, *E*_cell_ = 1.7 V while for DSA, *E*_cell_ = 1.46 V, (f) reaction mechanisms of PEF and PEC/PEF systems. Reproduced with permission from ref. 137. Copyrights Elsevier, 2024. (g) Removal efficiency, (h) mineralization efficiency of effluent from different filtration processes, (i) concentration of fluorine ion in the effluent and the defluorination efficiency of different processes. Reproduced with permission from ref. 138. Copyright Elsevier, 2020. (j) Schematic diagram of photoelectron-Fenton reactors, (k) acetaminophen removal and (l) TOC removal for different processes operated at optimum conditions (electro-Fenton process (□) and Fenton process (◇) operated at EF optimum: [Fe^2+^] = 0.087 mM, [H_2_O_2_] = 16.3 mM, current density = 38 A m^2^; photoelectro-Fenton process (○): [Fe^2+^] = 0.08 mM, [H_2_O_2_] = 14.8 mM, current density = 38 A m^−2^), (m) proposed acetaminophen degradation pathway. Reproduced with permission from ref. 178. Copyrights Elsevier, 2022.

For instance, Orimolade *et al.*^136^ used cathodic electro-Fenton and anodic PEC oxidation in the same cell to mineralize paracetamol. The catalyst was a thermally treated carbon felt (T-CF) as the cathode, which acts as an efficient *in situ* electroactive surface area for two-electron O_2_ reduction to H_2_O_2_ production, the photoanode was an FTO-supported BiVO_4_/BiOI composite photoanode under visible light irradiation. The PEC supplied the holes and the anodic ˙OH produced from the oxidation of water, and the EF produced the ˙OH from the Fenton reaction between the cathodically produced H_2_O_2_ and the added Fe^2+^. The dual ˙OH supply, one photoanodic, one Fenton-derived the mechanistic core of the synergy. Individually, the EF system was able to achieve 71% removal of the TOC after 4 h at 20 mA cm^−2^, while the EF/PEC coupled system achieved 92% TOC removal in the same duration at 10 mA cm^−2^, with an energy saving of 64% (Fig. 8a). Low effluent toxicity towards Vibrio fischeri confirmed not only decolorization but also detoxification (Fig. 8b). Its heterojunction of BiVO_4_/BiOI played a key role in its band alignment, where both phases were staggered, allowing charge separation and visible-light absorption to go beyond that of a single phase. The limitation is the requirement for acidic pH (≈3) to maintain Fe^2+^ solubility, and the use of an added iron salt, which generates Fe(OH)_3_ sludge upon neutralization.

Liu *et al.*^137^ engineered a synergistic PEC/PEF configuration by hydrothermally growing cone-structured TiO_2_ arrays on nickel foam (TiO_2_/NF) coupled with a natural air-diffusion electrode (NADE) (Fig. 8c and d). Complete elimination of 10 mg per L carbamazepine (CBZ) was achieved within 30 min, yielding a pseudo-first-order rate constant of 0.1939 min^−1^ (Fig. 8e), under the remarkably low operating conditions of 2 mA applied current (1.7 V cell potential) and an electrical energy per order (EEO) of merely 0.005 kWh per m^3^ per order (This value, however, was obtained in a 2 mA, small-volume batch reactor treating a single model pollutant (10 mg per L CBZ) over 30 min, and should not be read as representative of pilot-scale performance). The mechanistic basis for this energy efficiency lies in the optoelectronic behavior of the TiO_2_/NF photoanode (Fig. 8f) UV-generated electron–hole (e^−^–h^+^) pairs drive photogenerated electrons toward the NADE cathode, amplifying local current density and substantially enhancing electrochemical H_2_O_2_ accumulation relative to conventional dimensionally stable anode (DSA)-based PEF systems. The resulting elevated ˙OH production (6.17 µM *vs.* 5.82 µM in PEF), combined with direct valence-band hole (h^+^) oxidation, collectively accounts for the superior degradation kinetics. Synergy coefficient 6.14–6.5 times higher than the single use of PEF indicates that the PEC and PEF components act multiplicatively rather than additively, making this architecture very interesting for refractory organic remediation in a low-energy paradigm.

Jiang *et al.*^138^ developed a UV-driven electro-Fenton catalytic membrane (UV-EFCM) filtration platform, which combines the PEC and electro-Fenton (EF) reactions in a sequential flow-through design (Fig. 8g and h). Excellent degradation (98.4 ± 9.1%) and mineralization (78.4 ± 9.1%) of florfenicol were obtained in a hydraulic retention time of 0.98 h. The significant result is the high defluorination efficiency of 56 ± 3.6% in the case of UV-EFCM compared to 11 ± 5.3% for EF-only filtration (Fig. 8i), which is most mechanistically significant. The high bond dissociation energy of the aromatic C–F bonds, which not only provides structural stability but also contributes to the retained antibacterial activity, means that the C–F bond is easier to break with UV photons than the electro-Fenton oxidation step, which reduces the activation barrier for the subsequent ring opening reaction. The complete loss of antibacterial activity demonstrates that the UV-pretreated product is not an electrophile, but rather an electrofuge, and it is not merely degrading the peripheral substituents, but the pharmacophoric core itself is being targeted.

de Luna *et al.*^178^ systematically compared EF and PEF processes for the remediation of acetaminophen using a double-cathode electrochemical cell (Fig. 8j) and by applying Box–Behnken response surface methodology (RSM) to deconvolute the impacts of initial Fe^2+^ concentration, H_2_O_2_ dosage, and applied current density. It was found that the initial concentration of Fe^2+^ was the most important factor for both processes, consistent with its rate-determining role in the Fenton catalytic cycle. When optimal conditions were used, EF and PEF were essentially the same: acetaminophen removal was 98% and 97%, COD reduction was 43% and 42%, and TOC removal was 19% and 20% (Fig. 8k and l). The negligible enhancement expected from UV illumination due to Fe^2+^ regeneration by the photoreduction of Fe^3+^ and the generation of additional ˙OH through the photolysis of Fe(OH)^2+^ indicates that Fe^3+^ reduction was not a kinetic rate-determining step under the conditions studied, or that the applied UV intensity was not sufficient to provide a meaningful contribution to the rate of Fe^3+^ reduction. Hence, EF was seen to be more cost-effective. The presence of thirteen transformation products and the proposed degradation pathway (Fig. 8m) contribute to a mechanistic understanding of sequential hydroxylation, deamination, and ring-cleavage reactions that are key for ecotoxicological risk assessment and design of downstream treatments.

All these studies confirm that the synergy between PEC and EF is not simply the UV overlaid on the conventional EF cells but depends on intentional architecture engineering of the photoanode. Liu *et al.*^137^ and Jiang *et al.*^138^ prove this with NADE mediated electron rerouting and membrane-integrated sequential oxidation, respectively, and de Luna *et al.*^178^ show that there is negligible incremental benefit to UV unless electrode level innovation is made, not parameter optimization. The quantitative evidence supporting this comes from Liu *et al.*^137^ whose EEO is 0.005 kWh per m^3^ per order. The UV-EFCM of Jiang *et al.*^138^ is mechanistically different because it enables the defluorination of an aromatic C–F bond, which is not achieved by either the other two systems tackling this critical challenge under standard electrochemical conditions. All three works suffer from shared limitations: synthetic matrices containing only a single contaminant overestimate the performance of the real effluents the longevity of photoanodes under sustained polarization has not been characterized; and mechanistic interpretation is based on indirect ROS quantification, which is not directly measured *in situ*. In all the studies the limit of TOC removal is set at ≤20% which indicates that it is thermodynamically and economically not feasible to mineralize the pollutants completely by electrochemical oxidation only. Visible-light-active photoanodes (BiVO_4_, Z-scheme heterojunctions), strong carbon-based cathodes to achieve continuous production of H_2_O_2_, and biological post-treatment coupling to close the mineralization gap are some of the future priorities to be explored. Lifecycle and techno-economic assessments remain conspicuously absent and are prerequisite for transitioning PEC/EF systems from proof-of-concept to scalable implementation.

### 4.5 PEC/ozonation

Combining PEC with ozone provides a powerful oxidation strategy. Ozone (O_3_) is a strong oxidant that can react directly with many organics or decompose into radicals.^179,180^ In a PEC/O_3_ hybrid, O_3_ exists in two forms as a chemical oxidase to oxidize contaminants, particularly electron-rich structures, and as a photolizer/electroreducer to generate more radicals (˙OH, O_2_˙^−^) at the electrode surface. This hybrid is designed to take advantage of the complementary effects of ozone's powerful, non-selective oxidation and PEC photogenerated ˙OH and charge carriers.^181^ Since ozone is usually added as a gas, PEC/O_3_ systems are by nature challenged with gas-to-liquid mass transfer issues and reactor engineering issues (*e.g.*, bubble columns, diffusers). The main synergy in visible-light PEC systems is through sequential cathodic O_3_ reduction, which results in the amplification of radicals, and O_3_ can then react with ˙OH to create more radicals.

In practice, the PEC/O_3_ system is designed to work with an ozone generator injecting ozone into the reaction cell. The main reactive species generated from O_3_ are ˙OH (generated through the decomposition of O_3_) and O_3_ itself, which is electrophilic towards electron-rich groups such as double bonds and amine groups.^182^ The photogenerated electrons reduce O_3_ to O_3_˙^−^ at the electrode surface (reaction (21)), followed by fast reaction with H^+^ to form ˙OH and O_2_ (reaction (22)).^183^ At the same time, holes generated by the light oxidize organics or water at the surface of the photoanode. One of the important differences between PEC/O_3_ and other hybrid systems is that O_3_ is not activated *via* electrodes but is reactive as soon as it is placed in the aqueous phase. Also, if UV components exist in the spectrum of the irradiation, direct ozone photolysis generates singlet oxygen and eventually ˙OH (reaction (23)). Therefore, when used alongside radical generation by PEC, ozone can lead to a very significant increase in steady state ˙OH concentration. Notably, no other stable chemical oxidant is needed other than O_3_ itself, and the semiconductor not only provides supplementary electrons and holes but may also activate the O_3_.

21 O_3_ + e^−^ → O_3_˙^−^

22 O_3_˙^−^ + H^+^ → ˙OH + O_2_

23 O_3_ + *hν* → O_2_ + O(^1^D) → 2˙OH

The available literature on PECO and related ozone-assisted PEC systems is rather limited, yet the evidence presented here clearly shows that using ozone in combination with PEC processes, significantly better degradation and mineralization of pharmaceutical substances can be achieved than by photocatalysis, PEC or ozonation alone. Based on the available studies, a critical analysis reveals that the greater efficiency of PECO is based on the synergy effect between electrochemically created charge carriers, photocatalytic oxidation routes, and ROS formed based on ozone.

**Fig. 9:**
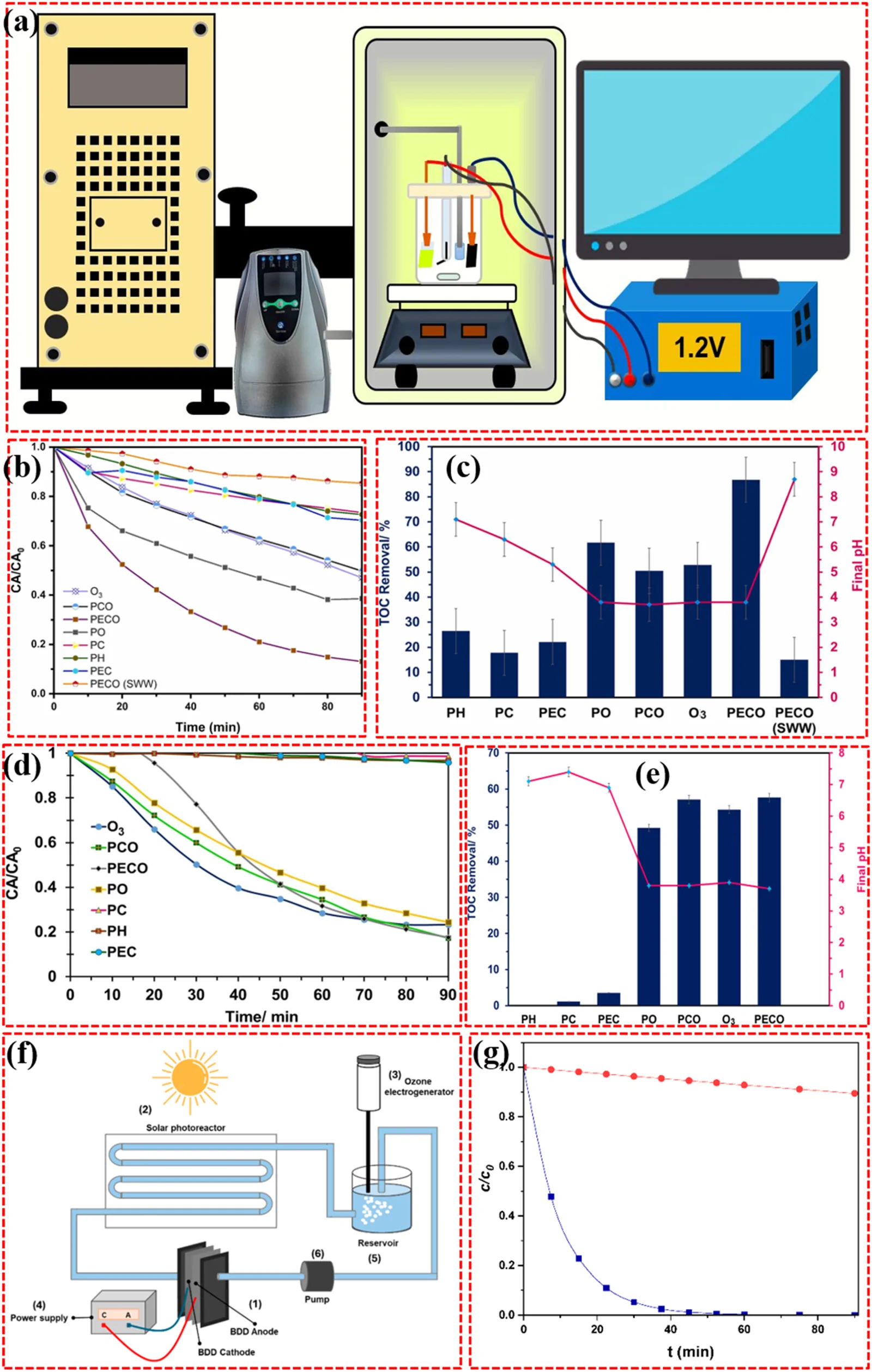
(a) Configuration of the catalytic photoelectric reactor: TiNTs-N working electrode, carbon paper counter electrode, Ag/AgCl reference electrode (3 M NaCl), Ozonator (O_3_ sterilizer) 18 W 500 mg h^−1^, Xenon lamp (150 W), and power supply, (b) normalized concentration collected during CIP degradation as a function of time obtained from UV-Vis spectra at 270 nm, in solutions containing 20 mg per L CIP and 0.05 mol per L Na_2_SO_4_ (distilled water) and SWW, (c) TOC removal percentages associated with ciprofloxacin elimination using different oxidation treatments labelled on the figure. Reproduced with permission from ref. 132. Copyrights Elsevier, 2022. (d) Normalized concentration of CFX destruction as a function of time obtained from UV/vis spectra at 264 nm, in solutions containing 20 mg per L CFX and 0.05 mol L^−1^ Na_2_SO_4_, (e) TOC percentage removal for CFX elimination using the treatments labelled on the figure, including the final pH obtained for each system. Reproduced with permission from ref. 139. Copyrights Elsevier, 2022. (f) Assembly of a CPC-type continuous flow reactor coupled with ozone. (1) filter press-type electrochemical cell, (2) CPC-type reactor, (3) ozone generator, (4) power supply, (5) reservoir, and (6) centrifugal pump, (g) normalized concentration for 20 mg L^−1^ of paracetamol by direct photolysis (red color) and solar photoelectro-Fenton (SPEF) processes (blue color) in a CPC-type reactor of 30 L and liquid flow rate of 300 L h^−1^. In all the tests, 0.05 mM Na_2_SO_4_ was used as the supporting electrolyte by adding 0.5 mM Fe^2+^ at *j* = 60 mA cm^−2^. Reproduced from ref. 184. Which is open access and permits unrestricted use of materials under the terms of Creative Commons CC-BY.

In the study, Palomares-Reyna *et al.*^132^ carbon and nitrogen co-doped TiO_2_ nanorough surfaces were electrochemically anodized and used to degrade ciprofloxacin (Fig. 9a). The PECO system was the most efficient system, with a ciprofloxacin degradation efficiency of 95% and a TOC degradation efficiency of 86.79% (Fig. 9b) in 90 min (Fig. 9c), among all investigated processes such as PC, PEC, O_3_, PCO, and PECO. Multiple interacting mechanisms account for improved performance. First, external bias in PEC inhibits electron–hole recombination, making more holes and electrons available that have been generated by the photons. Second, ozone is also a strong electron acceptor, which in turn can lead to the scavenging of electrons and facilitate the formation of ˙OH, O_2_˙^−^, and other ROS. Radical scavenging experiments showed that holes, O_2_˙^−^, and ˙OH all participated in pollutant degradation. In addition, the carbon and nitrogen doping resulted in a longer light absorption time for TiO_2_ and increased the efficiency of charge separation, which led to a higher utilization of the incident irradiation. The high mineralization efficiency not only shows the ability of the PECO system to transform ciprofloxacin into intermediate products but also shows a high degree of oxidation to CO_2_ and H_2_O, which is essential for wastewater treatment applications.

The same research group studied the degradation of cefadroxil by a similar process,^139^ and found the same type of depolymerization. The carbon and nitrogen co-doped TiO_2_ photoanode demonstrated good visible-light absorption, which was attributed to the introduction of TiO_2_ impurity energy states. The PECO process was more effective than the ozonation and photocatalytic ozonation processes in degrading cefadroxil with 96% in 90 min (Fig. 9d) and removed 57.6% TOC after 90 min (Fig. 9e). The degradation efficiency was similar to that achieved for ciprofloxacin; however, the extent of mineralization was considerably less than observed for ciprofloxacin. This difference indicates that the oxidation of cefadroxil produced more stable intermediate products, such as short-chain carboxylic acids, which are typically more stable to oxidative attack than the parent drug molecule. Despite this, the improved germination of lettuce seeds following PECO treatment showed the ability of the process to reduce wastewater toxicity, suggesting that, besides the percentage of degradation, it is necessary to consider the reduction of toxicity and mineralization performance.

More recently, Herrera-Chávez *et al.*^184^ expanded the concept of ozone-assisted PEC oxidation to a pilot-scale solar PEF system combined with ozone (Fig. 9f). In this work, BDD electrodes, instead of TiO_2_-based PECO studies, were used together with solar irradiation to degrade pharmaceutical mixtures related to COVID-19 treatment. In a complex four-drug mixture, the SPEF-O_3_ process resulted in 60% degradation of the pharmaceuticals (Fig. 9g) and 41% removal of COD. The values were lower compared to the values obtained in single-drug laboratory systems, but the challenge for the treatment was more significant in the presence of multiple pharmaceuticals and pilot-scale operating conditions. The improved performance was mainly attributed to the synergistic production of ˙OH by the combination of Fenton chemistry, electrochemical oxidation, solar photolysis, and ozone decomposition. The results show that ozone is also very effective in enhancing other PEC processes than conventional TiO_2_-based PECO.

These studies show some key trends when compared. First, ozone is always superior in oxidation performance in all the PEC configurations used. Second, degradation efficiency is not the only factor that affects the degree of mineralization, but also the structure and matrix complexity of pollutants. Third, catalyst engineering is an important factor that determines the advantages of ozone coupling; in particular, the introduction of heteroatoms and the development of advanced electrode materials. Lastly, the apparent treatment efficiencies also decrease as the system moves from laboratory-scale single-pollutant systems to pilot-scale complex wastewater matrices, giving an indication of the practical usability of the system.

Despite the encouraging outcomes, ozone-assisted PEC systems are one of the least studied fields of advanced oxidation technologies. There are only a few studies that have examined PECO processes, and there is a great lack of information on reaction mechanisms, long-term electrode stability, and efficiency of ozone utilization, energy use, degradation pathways, and the generation of potentially harmful transformation products. Further development of visible-light-responsive photoelectrodes, understanding of the role that ozone plays in interfacial charge-transfer processes, optimization of ozone dosage and electrical bias conditions, real wastewater matrices evaluation, and pilot- and full-scale demonstrations should be targeted in future research. Moreover, the synergy of novel materials like MXenes, single-atom catalysts, metal–organic frameworks, and heterojunction semiconductors with the ozone-assisted PEC systems could provide new opportunities to further improve radical generation efficiency and treatment efficacy. Therefore, although the present studies are already ample evidence of the benefits that can be drawn from PECO systems, this field is still in its early stages and requires a significant amount of future research and development to make it a practical option for large-scale wastewater treatment.

### 4.6 PEC/bio hybrid systems

The integration of PEC with biological treatment strategies has emerged as a highly promising approach for the removal of pharmaceutically active compounds (PhACs) from wastewater. PEC systems can generate *i.e.*, ˙OH, O_2_˙^−^, and persulfate radicals through the concerted action of photon absorption, semiconductor photoexcitation, and applied bias.^185^ They often face challenges including high energy consumption, incomplete mineralization, and sensitivity to complex real-water matrices. The PEC/bio hybrid systems overcome these limitations by means of a synergistic cooperation between the PEC and the bio section.^186^ Depending on the system configuration, the PEC component can oxidatively transform the recalcitrant PhACs into structurally simpler, less toxic and more biodegradable intermediates that are subsequently degradable by the bio component.^185^ Whereas the latter can provide electrons to the PEC reaction, generate H_2_O_2_*in situ* for use in the Fenton-type reaction, or buffer the operational pH of the system. This is a mutual benefit, significantly lowering external energy requirements, unwanted intermediate build-up and enabling degradation processes otherwise not achievable. The following subsections review key recent advances in PEC/bio hybrid configurations for PhAC removal, encompassing bio-electro-Fenton (BEF) systems, self-powered photo-bio-electrochemical systems (PBES), persulfate-photo-bioelectrochemical systems, PEC-microbial fuel cell (PEC-MFC) coupling, and enzyme-integrated bio-photoelectrochemical systems (BPECS).

**Fig. 10:**
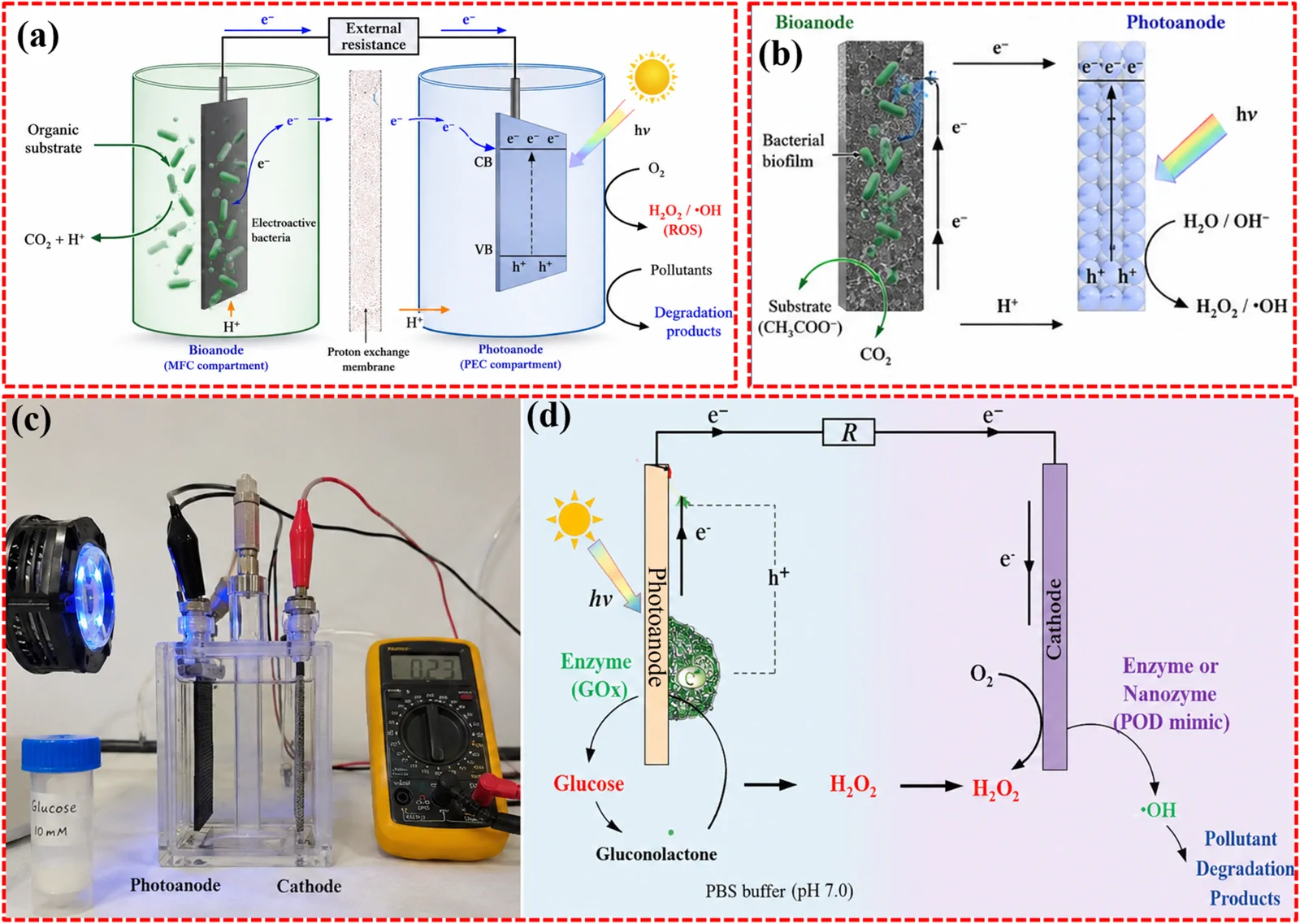
(a and b) Schematics of MFC-coupled PEC architecture. (c and d) Schematic of enzyme-integrated bio-photoelectrochemical configurations redox enzymes immobilized on functionalized photoanodes couple biochemical oxidation (*e.g.*, of glucose).

The mechanistic basis of PEC/bio hybrid systems varies across configurations but consistently converges on the principle of enhanced ROS generation and improved charge utilization. In MFC-coupled PEC architectures (Fig. 10a and b), electroactive bacteria oxidize organic substrates at the bioanode and release electrons that flow through an external circuit to drive PEC reactions at the semiconductor electrode rendering the overall system self-sustaining or even self-powered.^187,188^ In BEF systems, the microbially generated electrical current drives cathodic reduction of dissolved O_2_ to H_2_O_2_, which then reacts with added or in situ-generated Fe^2+^ to produce ˙OH *via* the classical Fenton reaction, eliminating the need for external H_2_O_2_ dosing. In PBES incorporating visible-light-responsive semiconductor photocathodes, photogenerated holes oxidize surface-adsorbed water or hydroxyl ions to produce ˙OH, while conduction band electrons reduce O_2_ to O_2_˙^−^, the bioanode simultaneously sustains continuous current flow, ensuring uninterrupted ROS generation.^9^ In enzyme-integrated bio-photoelectrochemical configurations (Fig. 10c and d), redox enzymes immobilized on functionalized photoanodes couple biochemical oxidation (*e.g.*, of glucose) with photocatalytic charge transfer, producing H_2_O_2_ at the cathode that is then activated by peroxidase enzymes or peroxidase-mimicking nanozymes to degrade target pollutants.^11^ Across all configurations, the interplay between photocatalysis, electrochemistry, and microbial or enzymatic activity creates a multi-ROS environment that substantially broadens the range of PhACs susceptible to degradation.

**Fig. 11:**
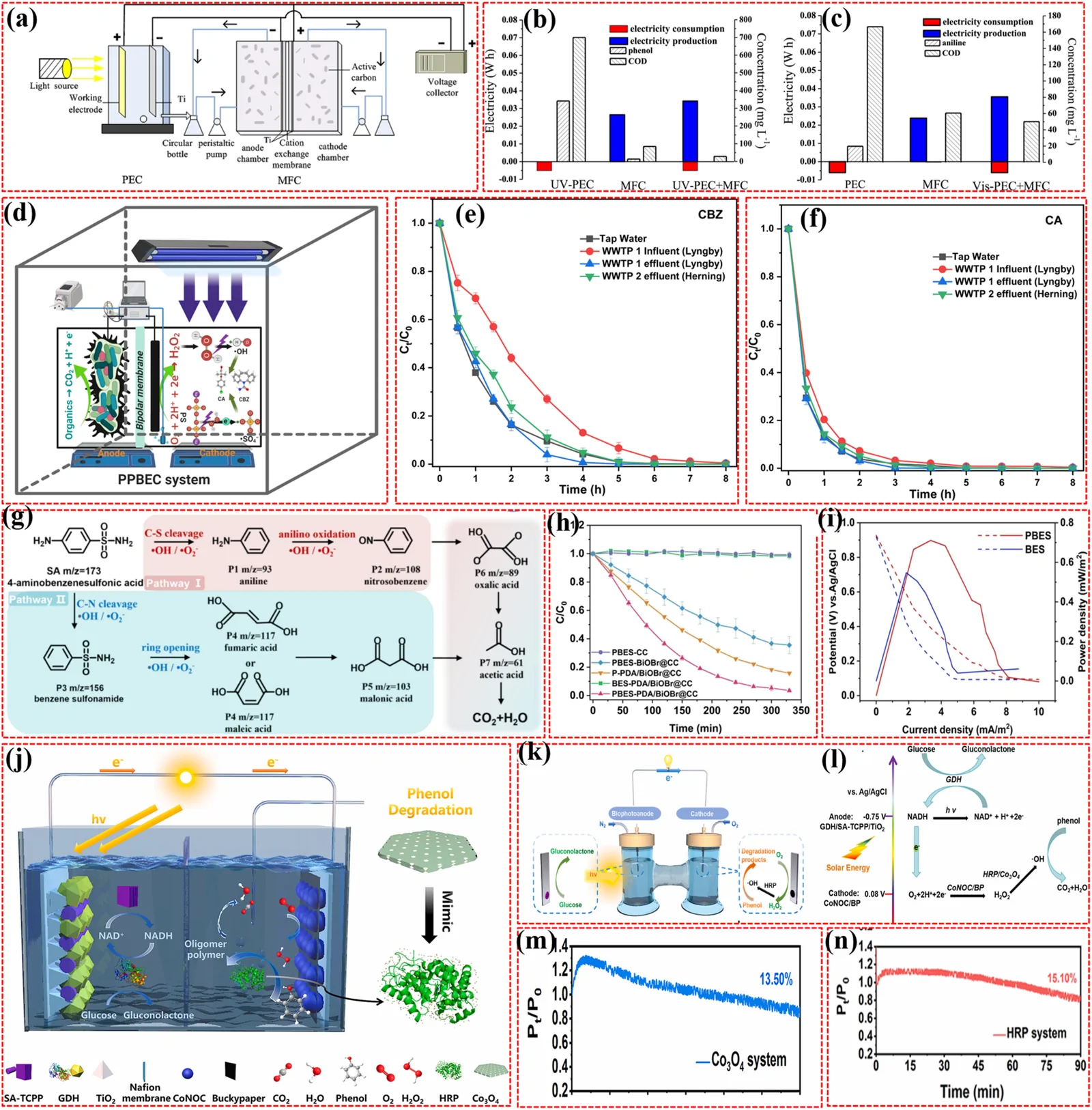
(a) Schematic of PEC-MFC system device, (b) and (c) the comparison of final concentrations of phenol and aniline and COD (the initial concentrations: 400 mg per L phenol and 50 mg per L aniline) and electricity production and consumption among three technologies (PEC, MFC and PEC-MFC combined process) after 6 h of treatment. Reproduced with permission from ref. 189. Copyrights Elsevier, 2019. (d) Schematic illustration of the PPBEC reactor, (e and f) the impact of water matrix on system performance. System operational parameters. Reproduced from ref. 140. Which is open access and permits unrestricted use of materials under the terms of Creative Commons CC-BY. The initial CBZ and CA concentrations of 10 mg L^−1^ were spiked into the selected water types, and other parameters were determined based on the earlier described tests, including PS dosage of 1 mM, applied voltage of 0.6 V, cathodic aeration velocity of 0.005 mL min mL^−1^, UV intensity of 10 mW cm^−2^ and without pH adjustment. (g) Proposed SA degradation pathways, (h) SA removal in PBES equipped with CC, BiOBr@CC, PDA/BiOBr@CC cathodes, including comparisons with photocatalysis (P) and BES modes using PDA/BiOBr@CC cathode (i) power density and polarization curves of PBES and BES systems with PDA/BiOBr@CC cathodes. Reproduced with permission from ref. 141. Copyright Elsevier, 2026. (j) Schematic diagram of the working mechanism of glucose/O_2_ bio-photoelectrochemical system for multi-energy conversion and phenol degradation, (k) schematic model of dual-chamber BPECS, (l) working mechanisms and electrons transfer path of BPECS, (m and n) the discharge stability of BPECS at 0.49 V. Reproduced with permission from ref. 190. Copyrights Elsevier, 2024.

For instance, Zhang *et al.*^189^ developed a combined photoelectrocatalytic-microbial fuel cell (PEC-MFC) system (Fig. 11a) and applied it to the simultaneous removal of two representative refractory organic pollutants, phenol and aniline, from wastewater, with concurrent *in situ* energy recovery. The system was designed such that the electrical current generated by the MFC through the oxidation of organic substrates by electroactive bacteria at the bioanode was directly applied to power the PEC reaction, establishing a self-sustaining energy loop. Performance evaluation revealed that the combined PEC-MFC process outperformed either individual process alone in terms of both pollutant removal and electricity production. COD removal efficiencies reached approximately 96% for phenol (from an initial concentration of 700 mg L^−1^ to 29 mg L^−1^) and 70% for aniline (from 165 to 49 mg L^−1^), with over 95% pollutant removal even at high initial concentrations (Fig. 11b and c). A particularly important mechanistic finding was that although the PEC component contributed only 16.5% and 43% to phenol and aniline removal, respectively, the PEC pretreatment of the wastewater produced quinones, hydroquinones, and low-molecular-weight organic acids as intermediates compounds that are highly bioavailable and electrochemically active. When these PEC-treated streams were fed to the MFC, they yielded significantly higher voltage output, coulombic efficiency, maximal volumetric power density, and lower internal resistance compared to untreated feed water. This bidirectional enhancement of PEC improving MFC performance, and MFC providing energy for PEC underscores the synergistic value of coupling the two processes and highlights the importance of intermediate product chemistry in hybrid system design.

Zou *et al.*^140^ addressed a fundamental limitation of conventional BEF systems namely, their requirement for strongly acidic operating conditions (pH 2–3), dependence on supporting electrolytes such as Na_2_SO_4_, and the generation of iron sludge as a secondary waste by proposing a novel hybrid persulfate-photo-bioelectrochemical (PPBEC) system (Fig. 11d). The PPBEC system was developed to treat real secondary wastewater effluent at neutral pH and low persulfate dosage, aiming at the removal of the two highly persistent pharmaceutical micropollutants, carbamazepine (CBZ) and clofibric acid (CFA). The system used a combination of UV light and persulfate activation in one reactor, and a bioelectrochemical cell to produce H_2_O_2_ and single-electron reduction of PS, which is further amplified by the UV light. Input voltage, cathodic aeration velocity and the dose of PS was systematically tested. Under optimal conditions (0.6 V applied voltage, aeration velocity of 0.005 mL min^−1^ mL^−1^, and 1 mM PS), the PPBEC system achieved 0.56–1.71-fold greater micropollutant removal compared to the individual UV/PS and PBEC processes alone (Fig. 11e and f), with a remarkable 93% reduction in energy consumption. The degradation transformation products of both CBZ and CFA were identified and probable degradation pathways were proposed. Effluent ecotoxicity assessment using Vibrio fischeri confirmed a non-toxic response, validating the system's capacity to produce environmentally safe effluent from complex pharmaceutical-laden secondary wastewater at circumneutral pH.

Ren *et al.*^141^ reported the development of a self-powered PBES featuring a polydopamine/BiOBr (PDA/BiOBr) composite-modified photocathode for the visible-light-driven degradation of sulfonamide (SA) antibiotics. BiOBr was selected as the photocatalytic semiconductor owing to its favorable visible-light bandgap and strong oxidizing valence band potential, while polydopamine (PDA) a bioinspired polymer was introduced as an electron-transfer mediator at the photocathode surface to facilitate efficient charge flow from the bioanode. In the proposed mechanism, PDA-mediated electron shuttling from the microbial bioanode to the BiOBr photocathode enabled the reduction of dissolved O_2_ to O_2_˙^−^, while photogenerated holes in the valence band of BiOBr oxidized surface-adsorbed H_2_O and OH^−^ to produce ˙OH under visible light irradiation. These ROS species preferentially attacked high electron-density sites in the SA molecule, cleaving C–S and C–N bonds that are characteristic of sulfonamide resistance (Fig. 11g). The PDA/BiOBr-modified PBES achieved 96.5 ± 1.1% SA removal (Fig. 11h) at a volumetric removal rate of 21.2 ± 0.1 g m^−3^ d^−1^ with a concurrent current density of 119.2 ± 10.4 mA m^−2^ (Fig. 11i), substantially outperforming unmodified PBES (2.0 ± 0.4% SA removal) and PBES-BiOBr without PDA modification (64.5 ± 6.1% removal). The system self-powered operation, high removal rate, and reduced intermediate toxicity position as a highly promising sustainable platform for antibiotic wastewater treatment.

Ding *et al.*^190^ presented a conceptually innovative bias-free PECS that simultaneously harvested solar and biochemical energy while degrading phenolic pollutants, addressing the dual challenge of energy conversion and environmental remediation in a single integrated device (Fig. 11j). The biophotoanode was constructed by immobilizing glucose dehydrogenase (GDH) on a self-assembled *meso*-tetrakis(4-carboxyphenyl)porphyrin (SA-TCPP)-sensitized TiO_2_ substrate, where the porphyrin sensitizer extended light absorption into the visible range and GDH enzymatically oxidized glucose to provide a continuous biochemical electron source. The cathode comprised a nitrogen/oxygen-doped cobalt single-atom catalyst (CoNOC) engineered for selective two-electron oxygen reduction, producing H_2_O_2_ as a by-product of the cathodic reaction. This cathode-generated H_2_O_2_ was then redeployed *in situ* by co-immobilized horseradish peroxidase (HRP) to oxidatively degrade phenol (Fig. 11k and l), achieving approximately 100% degradation efficiency within 60 min. The BPECS delivered a maximum power density of 296.98 µW cm^−2^ at 0.49 V with no applied external bias, demonstrating genuine simultaneous energy harvesting and pollutant remediation. To address the practical limitation of natural enzyme stability, the authors further substituted HRP with Co_3_O_4_ nanozymes peroxidase-mimicking inorganic catalysts with superior stability and found that comparable phenol degradation (∼100%) was achieved within 90 min (Fig. 11m and n), confirming the scalability and robustness of the BPECS framework. This work represents a significant conceptual advance in integrating renewable energy generation with environmental remediation in a bias-free, enzyme- or nanozyme-mediated photoelectrochemical architecture.

A cross-examination of the across these studies reveals that PEC/bio hybrid configurations achieved high PhAC removal efficiencies ranging from 95% in the PEC-MFC system to 99.04% in the BEF system and near-complete removal in the bias-free BPECS, confirming that the hybrid approach is broadly effective irrespective of the specific biological sub-process employed. From an energy standpoint, BEF-based and PEC-MFC systems achieve energy-autonomous or net-positive operation by harvesting bioelectricity from the microbial anode, with the PEC-MFC configuration uniquely demonstrating that PEC-generated intermediates (quinones and low-molecular-weight organic acids) can reciprocally enhance MFC power output a bidirectional energy synergy not replicated in other configurations. The PPBEC system achieved the most dramatic reduction in energy consumption (93% *vs.* individual processes), while operating at neutral pH without iron sludge generation, making it the most operationally practical configuration for real wastewater treatment. In contrast, conventional BEF systems still require acidic conditions (pH 3–4) for optimal ˙OH generation, although Fe-functionalized cathodes can extend effective operation toward near-neutral pH an important step toward deployment compatibility with municipal or hospital effluents that cannot tolerate upstream acid addition.^141^ As for the pharmaceutical and ecotoxicological validation, systems with single compounds had the highest absolute values for pharmaceutical removal, while multi-pharmaceutical matrices and real secondary effluent had somewhat lower but more practically relevant values. Critically, ecotoxicity was assessed in only three of six reviewed studies and in each case it was confirmed that a genuine degradation step was achieved, including a fivefold reduction in intermediate toxicity and a non-toxic Vibrio fischeri endpoint, indicating that ecotoxicological endpoints should be a standard output of future investigations into hybrid systems, as partial degradation could lead to products of similar or greater environmental concern than the parent PhAC. Precise co-engineering of materials and biology at the interface of the electrode, using single-atom catalysts, bioinspired electron mediators, and immobilized enzymatic cascades, has enabled configurations with the most architecturally complex designs reviewed-those that reach performance levels beyond simple electrode designs.

## 5. ROS in hybrid PEC systems

The classification of hybrid PEC systems establishes how different auxiliary processes and oxidants are integrated with PEC to enhance charge separation and pollutant degradation. However, the classification alone does not explain why these configurations exhibit different degradation efficiencies. The key link between system configuration and treatment performance is the generation, transformation, and utilization of ROS. Depending on the hybrid configuration, charge-transfer processes and auxiliary oxidants can alter the type, abundance, lifetime, and reaction pathways of ROS, thereby determining the extent and selectivity of pharmaceutical oxidation. Understanding these ROS pathways is therefore essential for interpreting how the hybrid systems discussed in Section 3 translate their structural and operational advantages into enhanced degradation and mineralization. Accordingly, this section examines the major ROS generated in hybrid PEC systems, their interconversion pathways, and their mechanistic contributions to pharmaceutical degradation. Advanced oxidation in PEC is fundamentally driven ROS such as ˙OH, SO_4_˙^−^, O_2_˙^−^, and ^1^O_2_. In hybrid PEC systems, engineering in which ROS are generated and how they interconvert is central to degradation kinetics, mineralization efficiency, and selectivity toward pharmaceuticals (Table 3). Hybridization with oxidants (H_2_O_2_, PMS/PDS, O_3_, *etc.*) or processes (electro-Fenton, ultrasound) further amplifies ROS for example, injected oxidants can be activated by photogenerated electrons or holes to yield new ROS (SO_4_˙^−^ or ^1^O_2_) beyond ˙OH.^191^ The applied bias in PEC induces separation of e^−^/h^+^ pairs, avoiding recombination.^114^ Thus, in PEC a larger production of ROS and enhancement of degradation/mineralization of pollutants as compared to PC can be attained.^192^ In such systems, the lifetime of ROS, redox potential, and diffusion behavior are important design parameters. For example, the lifetime of ˙OH is only nanoseconds, and is very short-lived and nonselective, while that of SO_4_˙^−^ is microseconds and is quite electrophilic and selective.^193^ O_2_˙^−^ is weaker (*E*° ≈ 0.94 V) and is generally an intermediate that gives rise to H_2_O_2_ or ^1^O_2_, which is comparatively long-lived (lifetime 3.5 µs) and highly selective towards electron-rich bonds.^194^ Current ROS engineering is focused on optimizing the band structure, surface chemistry, and co-oxidants of photocatalysts to favor the activation of ROS pathways. For instance, the transition from indiscriminate oxidation by ˙OH to selective oxidation by SO_4_˙^−^ and nonradical processes (^1^O_2_ or direct electron transfer) in complex matrices has been improved. This section summarizes the basic ROS pathways in the hybrid PEC system and highlights charge-transfer mechanisms, ROS interconversion, and mechanistic contributions in pharmaceutical degradation.

**Table 3:** Summary of the characteristic redox potentials, lifetimes, and mechanistic roles of major ROS and DET pathways in hybrid PEC systems^a^

Oxidative species/pathway	Characteristic redox potential	Lifetime	Principal pathway/role
˙OH	Highest among the major ROS discussed	<1 ns	Highly reactive, non-selective oxidation; major contributor to mineralization
SO_4_˙^−^	2.5–3.1 V	µs range	Electrophilic/selective oxidation; generated mainly through PMS/PDS activation
O_2_˙^−^	≈0.94 V	Up to ms under alkaline conditions	Intermediate/electron shuttle; precursor to H_2_O_2_ and ^1^O_2_
^1^O_2_	≈2.2 V	3.5 µs in water	Selective oxidation of electron-rich functional groups
DET	Surface-dependent; no single universal potential	Surface-confined	Direct electron abstraction from adsorbed pharmaceuticals without diffusive ROS

a DET does not have a universal redox potential or lifetime because it is a surface-confined electron-transfer process governed by the semiconductor/electrode surface properties and pollutant adsorption, rather than by a freely diffusing ROS.

### 5.1 Radical pathways

Radical ROS (˙OH, SO_4_˙^−^, O_2_˙^−^) are typically generated by semiconductor excitation coupled with oxidant activation. PEC is based on the promotion of a semiconductor through the action of the UV/visible photons, creating electron–hole pairs (reactions (8) and (9)). The valence-band holes oxidize surface water or the lattice OH^−^ to ˙OH, and the conduction-band electrons reduce dissolved O_2_ to superoxide.^195^ Molecular oxygen may also be reduced by electrons in a two-electron pathway to hydrogen peroxide (reaction (24)) that can store additional ˙OH.^196^ These radicals are amplified by the addition of oxidants or electrolytes in hybrid PEC systems. For instance, the Fenton type activation (reaction (19)) is extensively used, particularly in the electro-Fenton systems. Likewise, PMS/PDS can accept electrons or react with catalysts to produce sulfate radicals, and ozone or ultrasound can produce ˙OH through cavitation or photolysis. Importantly, these pathways interact with O_2_˙^−^, a relatively weak oxidant, which generates stronger oxidants (H_2_O_2_, ^1^O_2_) and acts as an electron shuttle, providing connections between radical and nonradical pathways.

Each radical has characteristic reactivity and limitations. ˙OH, has the highest oxidation potential and reacts with nearly all organics, which makes it ideal for complete mineralization. However, its lifetime (<1 ns) is extremely short and it is readily quenched by background ions (Cl^−^, CO_3_^2−^) or excess oxidant.^197^ In practice, ˙OH is generated in PEC/H_2_O_2_, PEC/electro-Fenton, PEC/O_3_, and sonophotocatalysis. For instance, Liang *et al.* designed an oxygen-vacancy rich TiO_2_ photoanode with an air-diffusion cathode that produces H_2_O_2_*via* two-electron O_2_ reduction. This system achieved 98.3% removal of target pollutants (*k* = 0.064 min^−1^) at only 0.5 V, attributing the performance to enhanced ˙OH formation from water and electrogenerated H_2_O_2_.^198^ Similarly, in PEC/electro-Fenton the *in situ* generation of Fe^2+^/H_2_O_2_ yields ˙OH near the cathode. In PEC/O_3_, catalytic ozonation on photoanodes produces ˙OH_ad_ (surface-bound ˙OH) and atomic oxygen (*O_ad_) that attack organics. Thus, ˙OH pathways are ubiquitous across hybrid systems, providing high mineralization rates.

Nevertheless, ˙OH pathways have drawbacks. ˙OH, exhibits an extremely short lifetime and diffusion length in aqueous systems, limiting pollutant–radical interactions, while excessive oxidant loading promotes radical–radical recombination (reaction (25)) and other scavenging pathways that reduce oxidation efficiency.^199,200^ H_2_O_2_ can break down *via* reaction (26) and can also react with 
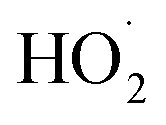
 at high concentration, reducing efficiency.^201^ Furthermore, ˙OH is not selective and attacks all moieties, which although useful for mineralization, reduces the selectivity, and may yield toxic byproducts. In contrast, SO_4_˙^−^ and nonradical oxidants provide more controlled oxidation. On a mechanistic level, the introduction of ˙OH sources in PEC primarily impacts the charge dynamics by scavenging electrons and by providing Fenton chemistry.^169^ But this benefit can be offset by the need for acidic pH (for Fenton) or careful dosing *e.g.* PEC/electro-Fenton typically requires pH < 4 to keep iron soluble, and H_2_O_2_ is unstable at high pH, limiting operational flexibility. ˙OH, pathways afford broad reactivity but suffer from short lifetime and poor selectivity. In H_2_O_2_ or O_3_-enriched PEC systems, in the cavitation effect provided by ultrasound, they are usually dominant and can only be practically applied if the scavenging effect can be controlled and the charge separation assisted.

24 O_2_ + 2e^−^ + 2H^+^ → H_2_O_2_

25 2˙OH → H_2_O_2_

26 H_2_O_2_ + *hv* → 2˙OH

#### 5.1.1 Hydroxyl radicals (˙OH)

**Fig. 12:**
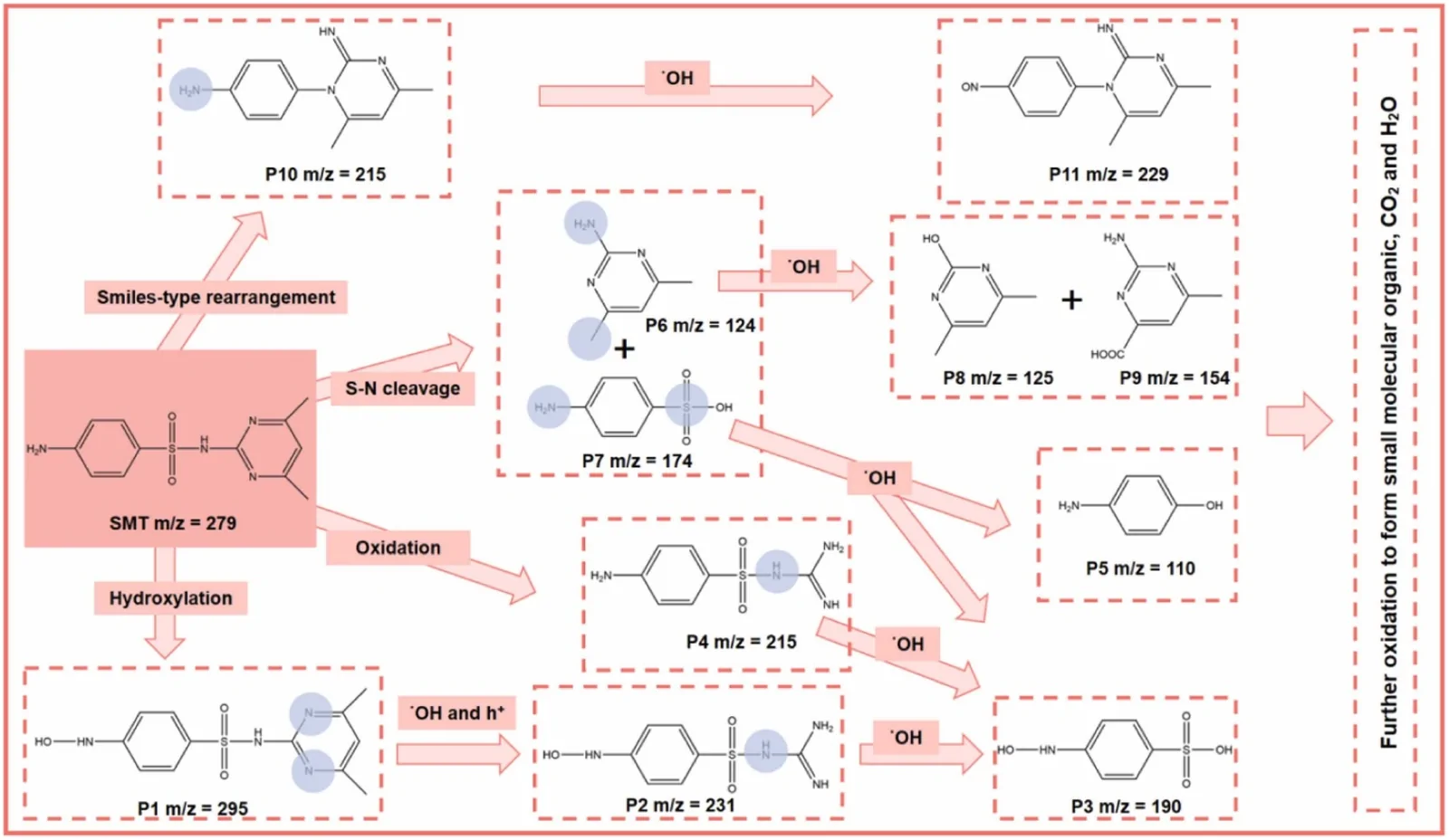
Proposed degradation pathways of contaminant SMT in b-TiO_2−*x*_/NADE PEC system. Reproduced with permission from ref. 198. Copyrights Elsevier, 2024.

˙OH, are formed through valence band oxidation of water/hydroxide, through the decomposition of water/hydroxide to H_2_O_2_ (photolysis or Fenton), or through attack of ozone. ˙OH, can also be generated from the photolysis or the Fenton reaction (reactions (15) and (19)) of H_2_O_2_ in the solution on illuminated photoanodes, usually through the action of the intermediates *O_ad_. In addition, the photogenerated holes oxidize H_2_O_2_ (reaction (26)). These mechanisms give the ˙OH its extreme oxidizing power (*E*° ≈ 2.8 V).^202^ In hybrid PEC, the bias helps to eliminate the recombination at the cathode and increase the yield of ˙OH at the anode, thus promoting further ˙OH formation and decreasing the recombination. For instance, Liang *et al.* reported that the degradation rate of model organics reached 98.3% in the PEC process, along with the simultaneous production of H_2_O_2_, which indicates that the production of ˙OH under low bias is enhanced, and the water can be purified efficiently (Fig. 12).^198^ But ˙OH pathways have been found to be limited. The lifetime of free ˙OH in water is very low, and radicals must form near the pollutant molecules to react,^203^ in addition, other radicals such as Cl^−^, C(O_3_)_2_^−^ can compete with the pollutant molecules, reducing the effectiveness of ˙OH even further.^163^ For example, radicals may be diverted as radicals of chloride by converting ˙OH to Cl˙ or HOCl.^204,205^ At high concentrations, H_2_O_2_ will self-quench (reaction (16)), thus decreasing the supply of radicals. In addition, the Fenton and photo-Fenton processes using ˙OH are only applicable to neutral waters, as the pH should be maintained at approximately 3 to keep Fe^2+^ in solution.^206,207^ In practice, thus, the PEC/H_2_O_2_ system and the PEC/electro-Fenton system need to find a compromise to compensate for these disadvantages and to optimize the ˙OH production. There are several scalability concerns related to this aspect, such as the expense of H_2_O_2,_ iron salts, and sludge disposal in Fenton. In addition, energy considerations suggest that it is advantageous to keep the production of ˙OH at a minimum, because of long irradiation or high current yield ˙OH, but at a decreasing rate. From a mechanistic point of view, in many pharmaceutical experiments, the ˙OH pathways are more prevalent, which is frequently demonstrated with the use of ˙OH-specific scavengers or ESR probes. However, various studies have documented the occurrence of incomplete mineralization because of radical scavenging or catalyst deactivation. Therefore, although ˙OH radical routes can lead to rapid degradation of many drugs, hybrid PEC design can also involve the use of additional ROS to overcome these drawbacks (as described below).

#### 5.1.2 Sulfate radicals (SO_4_˙^−^)

**Fig. 13:**
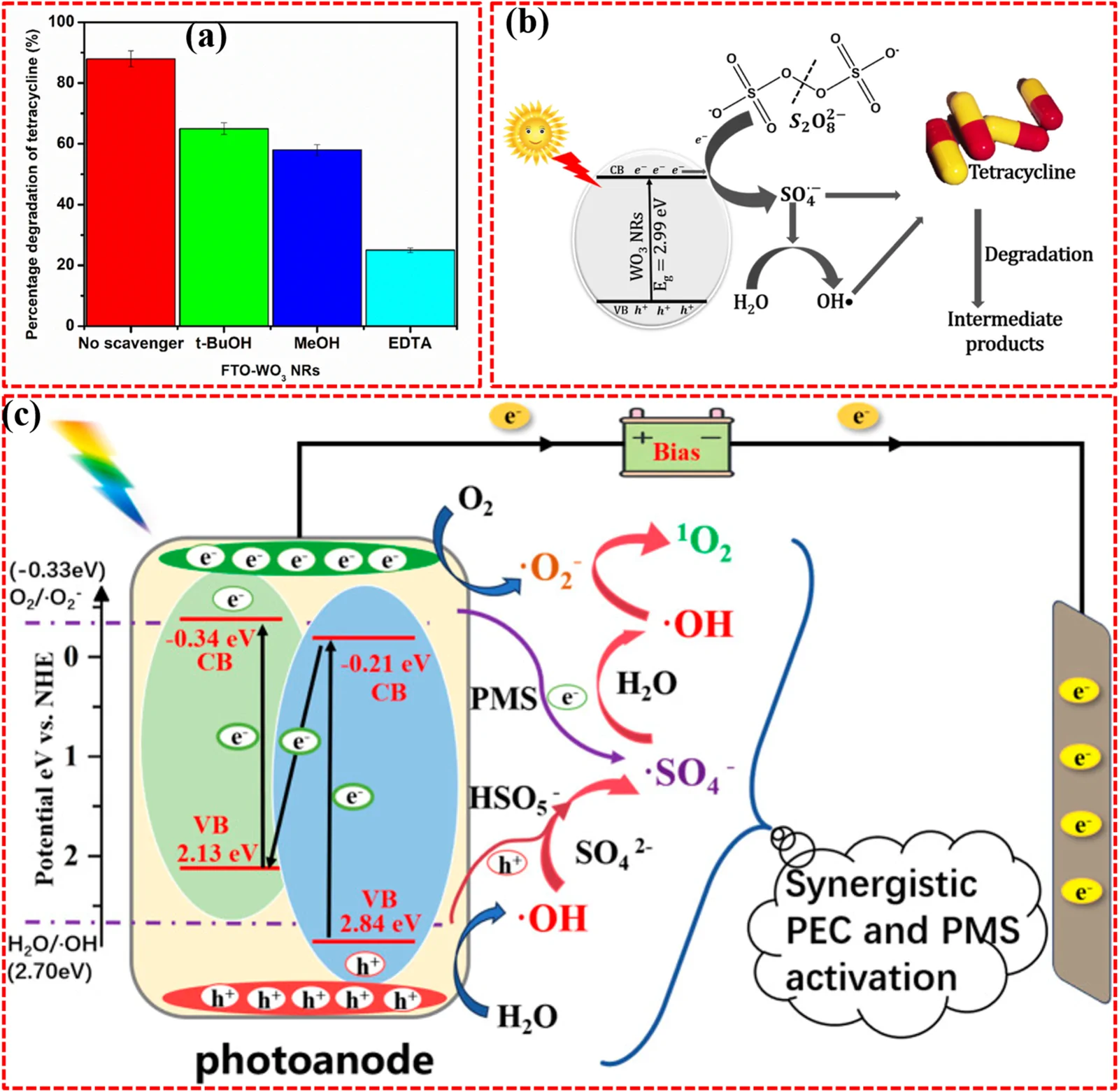
(a) Trapping experiment for the PEC degradation of tetracycline, (b) schematic diagram of the activation of PS for enhanced degradation of tetracycline. Reproduced from ref. 208. Which is open access and permits unrestricted use of materials under the terms of Creative Commons CC-BY. (c) Degradation pathways of tetracycline using PEC/PMS. Reproduced from ref. 209. Which is open access and permits unrestricted use of materials under the terms of Creative Commons CC-BY.

Sulfate radicals can be produced from the activation of persulfate species, namely peroxymonosulfate (PMS, HSO_5_^−^) or peroxydisulfate (PDS, S_2_O_8_^2−^). These anions may also gain electrons or be catalyzed to give SO_4_˙^−^. The important steps in PEC/PMS or PEC/PDS systems are the electron transfer reaction and the photochemical cleavage reaction (reaction (6) and (12)) and the competing two-electron reduction pathway that fully reduces the oxidant without radical formation (reaction (13)). Metals also activate persulfates; for example, Fe^2+^ catalyzes PMS like a Fenton reaction (reaction (27)). Therefore, the photogenerated electrons at the cathode or conduction band, as well as the holes at the photoanode, can be used to transform PMS/PDS into sulfuric radicals. In the case of Orimolade *et al.* used a Bi_2_WO_6_ photoanode in a PEC/PMS system, and 98% of sulfamethoxazole was removed after 90 min, with 3 mM PMS scavenging tests showing that SO_4_˙^−^ (and ˙OH) were the dominant oxidants.^121^ Likewise, Koiki and Arotiba showed a PEC/PDS process using WO_3_ photoanode, where 3 mM PDS increased the tetracycline degradation from 10% (PEC alone) to 99% (90 min, 1.5 V). In that work, trapping experiments indicated that all the radicals, SO_4_˙^−^ and ˙OH (and h^+^), were reactive in pollutant degradation, confirming the mixed-radical nature of PEC/PDS (Fig. 13a and b).^208^ In the case of Zhang *et al.* a TiO_2_/g-C_3_N_4_ photoanode was used in combination with PMS (0.2 mM, 1.0 V) to achieve 96% tetracycline degradation within 12 min. They discovered that the PEC/PMS system significantly enhanced electron transfer, generating ˙OH and SO_4_˙^−^ (as shown by kinetic analysis, the radical pathways were dominant with little contribution of ^1^O_2_) (Fig. 13 c).^209^ The complementary properties of SO_4_˙^−^ to ˙OH. They possess a high redox potential (2.5–3.1 V), although slightly lower in practice than ˙OH.^193^ Importantly, SO_4_˙^−^ is more selective and is less reactive toward highly resistant bonds, but is more reactive toward electron-rich sites (aromatic rings, phenolic groups).^210,211^ It has a significantly longer lifetime than ˙OH, leading to longer diffusion range and higher steady-state concentrations. Furthermore, SO_4_˙^−^ formation takes place over a wider pH range, PMS works over a pH range from acidic to neutral, and PDS works in alkaline conditions where SO_4_˙^−^ is converted to ˙OH and O_2_. These characteristics usually result in increased mineralization of pharmaceuticals.

However, SO_4_˙^−^ pathways also have challenges. The bond dissociation energies of PMS/PDS are high, and activation rates can be slower and need more energy input (UV or bias) than H_2_O_2_. If the excess of PMS/PDS is beyond control, scavenging by inorganic anions (*e.g.*, Cl^−^ converts SO_4_˙^−^ to Cl_2_˙^−^) or self-recombination can occur. Catalyst surface engineering (*e.g.*, oxygen vacancies, doped metals) is often needed to enhance persulfate activation. For instance, defective catalysts with strong PMS adsorption have been shown to generate ^1^O_2_*via* hole oxidation routes, but otherwise only radicals form.^194^ Also, the SO_4_˙^−^ may eventually give rise to sulfate ions, which may be present in treated water. SO_4_^2−^ is not harmful, but it can cause salinity at high concentrations and require regulation. Critically, SO_4_˙^−^ and ˙OH pathways are synergistic and trade off. In many hybrid systems, both are present PMS/PDS activation on a photoanode yields SO_4_˙^−^, which can further react with water to form ˙OH under alkaline conditions (reaction (7)). Balance is dependent on pH and the catalyst. Mechanistic studies show that contributions from both SO_4_˙^−^ and ˙OH are present in both PEC and PMS. Overall, SO_4_˙^−^ will offer greater selectivity and robustness in saline matrices but may need higher activation conditions. Optimization of persulfate dosage and design of the catalyst is needed in the future to obtain high yields of SO_4_˙^−^ with minimum energy cost and scavenging losses.

27 Fe^2+^ + HSO_5_^−^ → Fe^3+^ + SO_4_˙^−^ + OH^−^

#### 5.1.3 Superoxide radicals (O_2_˙^−^)

Single-electron reduction of O_2_ (reaction (9)) produces O_2_˙^−^.^134^ In PEC, this takes place at the cathode, conduction band. O_2_˙^−^ is also a relatively weak oxidant (*E*° ≈ 0.94 V) that reacts slowly with many organic contaminant.^212^ It plays its key role as a precursor for ROS networks. For instance, two O_2_˙^−^ can be disproportionate in an acidic medium to form H_2_O_2,_ which can react with Fe^2+^ to generate ˙OH,^213,214^ or react with UVA to generate ^1^O_2_. In solution, protons can form hydroperoxyl radicals 
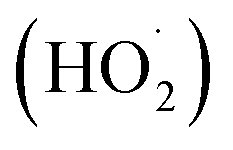
 from O_2_˙^−^, and 
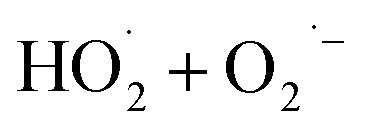
 (with H^+^) can generate H_2_O_2_ and ^1^O_2_*via* the Khan–Kasha dismutation pathway (reaction 10). Thus, O_2_˙^−^ links to other ROS generation, it can react with holes (reaction (28)) or lead to 1O_2_ by chain reactions. Most studies are able to observe the presence of superoxide (OOH-DMPO signal generation in ESR) but do not record it as the predominant oxidant in the treatment of pharmaceuticals. Instead, O_2_˙^−^ acts as an electron shuttle.^215^ For instance, in photoelectrochemical H_2_O_2_ generation systems, O_2_˙^−^ formation and its subsequent conversion to H_2_O_2_ (or directly to 1O_2_) is crucial for driving downstream oxidation. As photogenerated electrons reduce O_2_ to O_2_˙^−^ or H_2_O_2_.^196^ That is, O_2_˙^−^ is sometimes a pool of intermediates for stronger species. Selectivity-wise, O_2_˙^−^ is less destructive to organic molecules compared to ˙OH or SO_4_˙^−^. It can, however, cause oxidative degradation either by the production of H_2_O_2_ or by direct attack on sensitive moieties (*e.g.*, electron-rich aromatic systems). Some diffusion occurs because of its long half-life, up to ms in alkaline media. On the negative side, O_2_˙^−^ can recombine with holes or catalytically disproportionate, reducing its availability. From an energetic point of view, the generation of O_2_˙^−^ is possible with moderate potentials at the cathode; therefore, PEC may generate some O_2_˙^−^ alone. In hybrid PEC with cathodic ORR, O_2_˙^−^ is intentionally formed as the end route to H_2_O_2_ (two-electron ORR) or H_2_O (four-electron ORR).^165,167^ Overall, O_2_˙^−^ is more of a supporting than a lead oxidant. Mechanistically, it is involved in the correlation of charge carriers with ROS amplification, which indicates the successful transfer of e^−^ to O_2_, while its fate significantly determines the oxidative outcome. For instance, the conversion of O_2_˙^−^ to H_2_O_2_ raises the ˙OH production, the conversion to ^1^O_2_ raises the selectivity.^216^ In PEC/electro-Fenton, the O_2_˙^−^ concentrations dictate the amount of *in situ* H_2_O_2_ for Fenton. For PEC/PDS, the presence of any dissolved O_2_ will enable the formation of more O_2_˙^−^. Therefore, the formation and consumption routes of O_2_˙^−^ play a significant role in the ROS landscape in hybrid PEC, although they may not directly influence many pharmaceutical oxidation reactions.

28 O_2_˙^−^ + h^+^ → O_2_

### 5.2 Non-radical pathways

Non-radical oxidation routes are gaining importance in hybrid PEC due to their potential for high selectivity and low scavenging. Production of singlet oxygen (^1^O_2_) and direct electron-transfer (DET) are non-radical pathways that require no free radicals such as ˙OH or SO_4_˙^−^. These pathways can help to enhance performance in complex waters where radical quenching is wasteful. Non-radical ROS (^1^O_2_) have long lifetimes and prefer attack on unsaturated bonds; DET oxidation is an oxidation process that occurs in surface-confined reactions, with no diffusive radicals.

#### 5.2.1 Singlet oxygen (^1^O_2_)

^1^O_2_ is the first exciting state of O_2_, known as singlet oxygen. It is a strong oxidant (redox potential 2.2 V *vs.* NHE) and has a relatively long lifetime (3.5 µs in water).^194^^1^O_2_ is not a radical, but it can be generated through energy transfer or electron transfer reactions. Typical formation routes for PEC/hybrid systems are energy transfer from a photosensitizer or semiconductor to triplet O_2_ (reaction (29)) and chemical pathways through recombination of radicals (reaction (30)). Here, reaction (29) represents a triplet–triplet energy transfer where an excited photocatalyst transfers energy to ground-state O_2_, producing ^1^O_2_. Some photocatalytic ^1^O_2_ generation has been attributed to disproportionation of two ˙OH as shown in reaction (30). Specialized catalysts or defects can usually enable ^1^O_2_ in practice. For example, Zhang *et al.* showed that on certain Ti-sites, photogenerated holes can directly oxidize PMS to a metal-peroxo intermediate that decomposes into ^1^O_2_. They also highlight that ^1^O_2_ normally comes from energy transfer or from O_2_˙^−^ oxidation, making it harder to produce than radicals.^194^ The ^1^O_2_ oxidation reaction is highly selective, reacting preferentially with electron-rich functional groups such as activated aromatics or olefins, and as a rule not saturated C–H bonds.^217,218^ This may be useful in selectively targeting certain pharmaceutical structures without affecting bulk organic matter. In addition, many inorganic ions can quench ˙OH, but not ^1^O_2_. These characteristics make ^1^O_2_ desirable for saline or complex wastewater. Conversely the moderate reactivity of ^1^O_2_ can lead to slower degradation rates than those of radical pathways. It is difficult to determine actual ^1^O_2_ contributions in many cases of non-radical behavior where the contribution is to a radical intermediate. Specific quenchers (such as sodium azide) and ESR spin traps (such as TEMP give TEMP-^1^O_2_ adduct) are frequently used to support mechanistic evidence for ^1^O_2_. In the case of tetracycline oxidation, the TiO_2_/g-C_3_N_4_/PMS system detected a small amount of ^1^O_2_ in addition to the major pathways of ˙OH and SO_4_˙^−^.^209^ In some instances, ^1^O_2_ is the predominant oxidant, for example, in solar-activated oxygen-doped g-C_3_N_4_ systems where ^1^O_2_ is preferentially generated rather than the ˙OH.^219^ The spin-statistics of converting ground O_2_ to ^1^O_2_ typically requires an energy mediator, while direct hole oxidation of PMS to ^1^O_2_ (*via* an anionic intermediate) has been suggested, leading to mechanistic controversies. In addition, it is difficult to differentiate between ^1^O_2_ and other oxidants in a complex PEC environment. In summary, ^1^O_2_ offers high selectivity and stability in harsh conditions (high Cl^−^, high pH), making it valuable for pharmaceutical removal in real waters, but it often coexists with radicals and requires special generation strategies.

29 Sens* + O_2_ (^3^∑*g*^−^) → Sens + ^1^O_2_ (^1^Δ*g*)

30 2˙OH → H_2_O_2_ + ^1^O_2_

#### 5.2.2 Direct electron transfer mechanisms

Direct electron transfer (DET) is an oxidation process of pollutants by the surface-adsorbed holes (or electrons) generated by the photochemical reactions without the production of diffusive ROS.^220,221^ In DET, the semiconductor or an electrode surface directly abstracts electrons from an adsorbed contaminant, effectively burning it on the surface. It is frequently used to account for high selectivity or in the absence of a radical marker to rationalize the oxidation. A conductive carbon or metal interface, for instance, can be used to facilitate direct hole oxidation of organics by generating pollutant cations or fragments. But, in practice, the proof of DET is elusive. It requires high contact between the pollutant and the catalyst surface. In some studies, photocatalysts modified with carbonaceous materials (such as graphene and CNTs) are considered electron highways to achieve DET pathways.^222^ It has also been suggested that some photocatalysts (BiOX, layered semiconductors) trap holes at lattice oxygen, and the lattice oxygen oxidizes adsorbed molecules.^223^ These reactions, however, do not give rise to free radicals, and they can only be inferred by selective outcomes and the lack of free radical signatures. The dominant surface oxidation route in the HAOPs is direct transfer of electrons between the adsorbed pollutants and the oxidant.^224^ This means that complexes at the surface (such as peroxide or metal-oxo) may participate in DET. For instance, Meng *et al.* demonstrated that *p*-chlorophenol could be efficiently oxidized with surface-adsorbed PMS, suggesting DET-type behavior.^225^ DET is more likely to remove the contaminants with high electron density and can be conducted in the presence of quenched radicals. Its disadvantage is that it is generally only applicable to highly specific surface chemistry and high binding of pollutants. In pharmaceutical PEC systems, DET could appear as degradation improvement even in the presence of radical scavengers, which reduce the ˙OH/SO_4_˙^−^ concentration, or in the presence of inert matrices.^226^ This scavenger-based evidence, however, is indirect and must be interpreted with caution: incomplete or non-linear inhibition upon scavenger addition can equally arise from scavenger self-decomposition, non-quantitative or pH-dependent quenching kinetics. It may also be due to competitive consumption of the scavenger by the primary oxidant (PMS/PDS/H_2_O_2_) rather than from a genuine surface-confined electron-transfer pathway. Several of the DET assignments discussed above and in the wider literature including the surface-adsorbed-PMS-mediated oxidation proposed for chlorophenols and the nonradical pathways described for carbon- and single-atom-catalyst systems.^224,225^ Rest on exactly this type of indirect, scavenger-inefficiency reasoning, or on structure–activity correlations and should therefore be regarded as DET-consistent rather than DET-confirmed. A study interrogated the catalyst-pollutant interface directly, for example through chemical-probe/stoichiometry/electrochemical analysis capable of discriminating single-from double-electron-transfer pathways and high-valent surface species.^227^ Which constitutes considerably stronger mechanistic evidence than scavenger tests alone. To our knowledge, none of the DET-related studies discussed in this review employed true *operando* spectroscopic tools (*in situ* FTIR of adsorbed intermediates, *in situ* XAS of high-valent surface species, or transient absorption spectroscopy of hole states) under actual illuminated, biased PEC conditions. DET therefore remains a mechanistically attractive but largely inferred pathway in the pharmaceutical-PEC literature. It offers high selectivity and avoids overconsumption of oxidants but claims of DET dominance should be treated as provisional unless supported by direct spectroscopic or electrochemical interrogation of the interfacial complex, rather than by scavenger-inefficiency alone.

## 6. Effect of operational parameters on hybrid PEC systems

Hybrid PEC systems depend on various interconnected working parameters. Each of the parameters (light, pH, pollutant and oxidant concentrations, electrolyte, bias voltage, and temperature) affects charge generation/separation, production of reactive oxygen species (ROS), catalyst surface chemistry, and mass-transfer kinetics. Importantly, hybrid PEC systems display a synergistic effect *i.e.*, external bias inhibits recombination processes, whereas additional oxidants (PMS/PDS/H_2_O_2_) and/or co-processes (ozonation, biological) provide new ROS pathways and/or mass transfer regimes. This section focuses on the effect of each parameter on hybrid PEC performance for the degradation of pharmaceutical products in the context of recent publications in peer-reviewed literature.

### 6.1 Effect of pH

The pH of the solution plays a significant role in hybrid PEC systems in several ways. It controls the speciation and surface charge of pollutants and catalysts, the stability and activation pathways of the oxidants (PMS/PDS/H_2_O_2_), and the redox potential of charge carriers. Many pharmaceuticals with amino or acidic functional groups tend to become protonated at low pH, which changes their charge and adsorption affinity on the photoanode.^228,229^ At the same time, the surface charge of semiconductors below the pzc may increase to become more positive, thereby increasing the adsorptive capacity of an anion. For instance, acidic pH helps the generation of SO_4_˙^−^ in PEC/PMS and PEC/PDS systems, since the PMS activation is best in pH 2–4.^230^ In contrast, too low pH can decompose oxidants non-productively. Every hybrid system comes with its own ideal pH zone. The overall effect of pH is to regulate oxidant speciation and the properties of the catalyst surfaces, which in turn determine degradation kinetics and pathways.

**Fig. 14:**
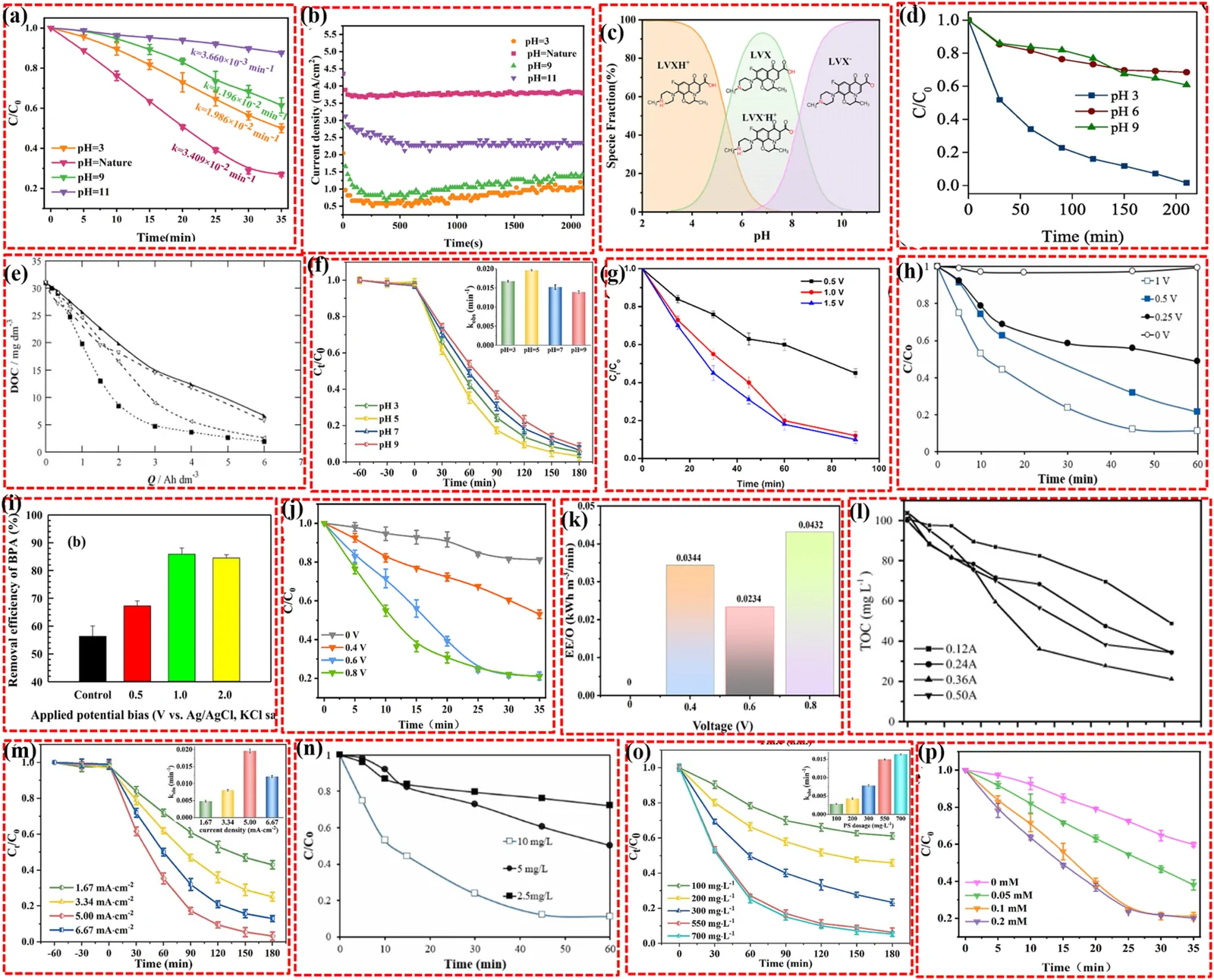
(a) Effect of initial pH on LVX degradation. (b) Current density under different pH. (c) LVX dissociation states *vs.* pH. Reproduced with permission from ref. 231. Copyrights Elsevier, 2026. (d) Normalized IBPA concentration *vs.* time recorded using Sn:Fe_2_O_3_–30 min during PEC degradation in 5 mM PMS (N_2_ bubbling; pH 3) under an applied 1.5 V *vs.* RHE at different pH values. Reproduced from ref. 232. Which is open access and permits unrestricted use of materials under the terms of Creative Commons CC-BY. (e) Effect of pH on DOC abatement of 41 mg per dm^3^ ibuprofen solutions with 0.5 mM Fe^2+^ and 0.05 M Na_2_SO_4_ treated by the PEF-BDD method at 33.3 mA cm^−2^ and 25.0 °C. Initial pH: () 2.0; (■) 3.0; (◁) 4.0; (▽) 6.0. Reproduced with permission from ref. 233. Copyrights Elsevier, 2009. (f) Effect of initial pH on acetaminophen. Reproduced with permission from ref. 234. Copyrights Elsevier, 2025. (g) Effect of applied bias potential on the PEC/PMS degradation process of sulfamethoxazole. Reproduced from ref. 121. Which is open access and permits unrestricted use of materials under the terms of Creative Commons CC-BY. (h) Effect of the applied potential on the removal of 1 mg per L EE2, PMS = 10 mg L^−1^; Na_2_SO_4_ = 0.05 M. Reproduced with permission from ref. 235. Copyrights Elsevier, 2026. (i) Effect of applied potential bias ([BPA]_0_ = 10.0 µM; [PDS]_0_ = 1.0 mM; [(NH_4_)_2_SO_4_]_0_ = 0.1 M; pH_*i*_ = 5.5). Reproduced with permission from ref. 131. Copyrights Elsevier, 2021. (j) LVX degradation under different applied voltages and (k) corresponding kinetic and rate constants. Reproduced with permission from ref. 231. Copyrights Elsevier, 2026. (l) Effect of applied current on TOC removal decay during the PEF treatment of 125 mL of 200 mg per L SMX solutions. Reproduced with permission from ref. 236. Copyrights Elsevier, 2026. (m) Current density on ATP degradation in the PEC system. Reproduced with permission from ref. 234. Copyrights Elsevier, 2025. (n) Effect of PMS concentration; EE2 = 1 mg L^−1^; PMS = 10 mg L^−1^; 1 V. Reproduced with permission from ref. 235. Copyrights Elsevier, 2026. (o) Effect of initial PS dosage on ATP degradation in the E-PS system (Mo–CuFeO_2_/CF). Reproduced with permission from ref. 234. Copyrights Elsevier, 2025. (p) LVX degradation at various PMS concentrations. Reproduced with permission from ref. 231. Copyrights Elsevier, 2026.

For instance, Li *et al.*^231^ designed a type-II Mo_72_Fe_30_/BiVO_4_ (MF/BVO) Photoanode to synergistically activate Photoelectrochemical (PEC) and PMS processes to degrade levofloxacin (LVF) efficiently. The degradation of levofloxacin (LVX) was studied by varying the solution pH at the start of the reaction, using the MF_3_/BVO photoanode, with HCl or NaOH. The degradation efficiency showed a clear pH dependence, with the best degradation around the natural pH of the reaction system (∼5.5) (Fig. 14a). The degradation performance decreased rapidly when the pH rose in the alkaline range, and about 10% removal of LVX was observed at pH 11 in the initial reaction period. This was attributed to the competitive adsorption of the reactive species, such as ˙OH and SO_4_˙^−^ radicals, on surface ≡Fe(iii) sites, thereby decreasing their interaction with the molecules of LVX and oxidation efficiency. Moreover, the electrochemical characteristics of the photoanode were negatively influenced by the extreme pH. At pH 3 and 9, the photocurrent density dropped to ∼1 mA cm^−2^ (Fig. 14b), suggesting that there is a loss of charge-transfer efficiency, which may be attributable to electrode corrosion. Additionally, the zeta potential results revealed that the surface charge of the MF_3_/BVO was negative in the entire range of pH under investigation. The electrostatic interaction between the catalyst surface and protonated LVX species (LVXH^+^) decreased as the zeta potential approached the isoelectric point of the catalyst at pH 3 (Fig. 14c). Thus, the adsorption efficiency and the degradation efficiency were lowered in a highly acidic solution.

Machreki *et al.*^232^ investigated the influence of the pH on the photoelectrochemical degradation of ibuprofen (IBPA) in the pH range of 3–9. The results revealed that degradation rates are significantly higher under an acidic environment, with degradation efficiencies of 99%, 30%, and 41% under the acidic pH of 3, 6, and 9, respectively (Fig. 14d). The significantly enhanced performance under acidic conditions indicates that solution pH plays a crucial role in determining both the generation and reactivity of oxidizing species. The enhancement of degradation at pH 3 was responsible for changes in semiconductor flat band potential and more efficient generation of sulfate radicals. Under acidic conditions, SO_4_˙^−^ are the predominant reactive species, and have an oxidation potential of 2.5–3.1 V *vs.* NHE, which is higher than that of ˙OH (1.8–2.7 V *vs.* NHE), making them more effective for oxidizing recalcitrant organic pollutants. The contribution of SO_4_˙^−^ became less as the pH increased towards the neutral to alkaline range, where SO_4_˙^−^ was converted to less reactive species such as ˙OH. In addition, the effect of surface property and charge-transfer characteristics of the semiconductor under alkaline conditions may cause further decreases in the efficiency of the pollutant oxidation. It is evident from the above results that the acidic media are favorable for the PEC degradation process involving the action of SO_4_˙^−^.

Skoumal *et al.*^233^ studied the effect of solution pH on the degradation of ibuprofen with a boron-doped diamond (BDD) anode and an oxygen-diffusion cathode at a fixed current density of 33.3 mA cm^−2^. Experiments were carried out at initial pH 2.0, 3.0, 4.0, and 6.0 and the outcome showed high mineralization efficiency on the pH of the solution (Fig. 14e). The degradation and highest rate of mineralization were achieved at pH of 3.0 and degradation was slower at pH of 4.0 although about 91% of DOC was removed after long electrolysis. In contrast, mineralization was significantly inhibited at pH 2.0 and 6.0, where only 79% and 82% of mineralization were achieved, respectively. This improved performance at pH 3.0 was attributed to the production of optimal amounts of hydroxyl radicals by Fenton chemistry. The reaction between Fe^2+^ and electrogenerated H_2_O_2_ achieves optimum efficiency at pH 2.8–3.0 and is rapid enough to generate homogeneous ˙OH radicals which effectively mineralize ibuprofen and its intermediates. Below pH 5.5, generation of hydroxyl radicals may be inhibited due to the formation of iron complexes and/or stabilization of H_2_O_2_ and above pH 5.5, the precipitation of iron species reduces the available soluble iron for the Fenton cycle. Thus, the efficiency of the oxidation process decreases, thereby validating the optimal operating condition of the PEF process for ibuprofen removal as pH 3.

Qin *et al.*^234^ studied the effect of initial solution pH on acetaminophen (ATP) degradation in a PEC system with electro-activated persulfate (PEC/E-PS) using a 3D nanostructured PbO_2_/Sb–SnO_2_ bifacial anode and a Mo–CuFeO_2_ modified with carbon felt cathode. The bifacial photoanode showed stable degradation performance over a relatively wide pH range (3–9) (Fig. 14f), indicating that it can be used for the degradation of wastewaters in different conditions. However, the highest degradation efficiency was obtained at pH 5, giving the first-order rate constant (*k*_obs_) of 0.0196 min^−1^. The improved degradation under mild acidic conditions was blamed on the more favorable production of ˙OH, which aids the transformation of sulfate ions into highly reactive SO_4_˙^−^. The synergistic effect of these oxidants remarkably promotes the oxidation of pollutants. Under alkaline conditions, however, ˙OH reacts with OH^−^ ions to produce oxygen-centered radicals(˙O^−^), which have much less oxidation potential than ˙OH and SO_4_˙^−^. Additionally, at high pH, the oxygen evolution reaction takes place, which results in an excessive number of cells forming on the surface of the electrode. Such bubbles prevent mass transfer, limit the pollutant from reaching active sites, and ultimately lower the efficiency of pollutant degradation. Thus, mildly acidic conditions were found to be best for ATP degradation in the PEC/E-PS system.

### 6.2 Effect of applied bias potential and current density

In a PEC cell, the externally applied anodic bias is a crucial lever to drive charge separation and redox reactions. Pure photocatalysis can be distinguished from PEC by the external bias. Applying a positive potential to the photoanode causes the photogenerated electrons to be removed (to the cathode), which reduces electron–hole recombination.^237^ This usually leads to higher photocurrent and enhances oxidant generation at the cathode. A higher bias increases the Fermi level of the holes on the anodic side, allowing for more aggressive oxidation of water or surface-adsorbed species to ˙OH.^238,239^ Therefore, the oxidation rates of pollutants generally increased at higher voltages. But, beyond an optimum range, higher voltage brings reduced benefits; current may be limited by light intensities or catalyst chemistry, and parasitic reactions like oxygen evolution may become significant, consuming energy and reducing the number of radicals produced per photon.

For instance, Orimolade *et al.*^121^ studied the influence of applied bias potential on the degradation of sulfamethoxazole using applied bias potentials of 0.5, 1.0, and 1.5 V. The results showed that increasing the bias potential led to a significant improvement in the degradation efficiency, and sulfamethoxazole was removed from 56% at 0.5 V to 98.2% at 1.0 V and 98.8% at 1.5 V (Fig. 14g). Improved separation of photogenerated electron–hole pairs within the photoanode was suggested to be responsible for the superior performance at higher bias potentials, which resulted in reduced charge recombination and facilitated more efficient PMS activation and generation of ROS. The degradation rate was also found to be higher for higher bias potential from kinetic studies. Nevertheless, the increase from 1.0 to 1.5 V was small, suggesting that the system had reached close to optimal charge separation by 1.0 V. The small increase in additional benefit at higher potentials was believed to be due to competing side reactions, such as oxygen evolution using part of the applied current, and lowering the current utilization efficiency.

Similarly, Dhawle *et al.*^235^ studied the effect of applied potential on the degradation of 17α-ethinyl estradiol with bias potentials of 0.25, 0.5, and 1.0 V. The degradation efficiency rose gradually as the applied potential was raised, and a maximum removal of 88.8% was achieved at 1.0 V, while only 51.3% was achieved at 0.25 V (Fig. 14h). The improvement was attributed to the effect of the external electric field on the migration of the photogenerated electrons to the cathode, which reduces the electron–hole recombination and enhances the availability of oxidative holes on the photoanode surface. This means the oxidation of organic pollutants was more effective. The authors also proposed that the potential applied more than 1.0 V is not expected to significantly affect degradation, as the process of oxygen evolution is expected to become more energetically favorable at higher applied anodic potentials. This side reaction reduces the current efficiency by withdrawing charge from the oxidation pathway of pollutants, and for this reason, 1.0 V is the optimal operating voltage.

Son *et al.*^131^ studied the performance of the PEC/PDS system for the degradation of bisphenol A (BPA) with varying applied biases. The results showed that electrical energy input was positively correlated with the efficiency of BPA removal, and the degradation rate was found to increase with the bias potential up to 1.0 V (Fig. 14i). The increased performance was primarily attributed to better charge carrier separation and efficient use of the photogenerated electrons and holes. The authors explained that the contribution of anodically generated persulfate from the electrolyte was very small for the operating conditions due to the low concentration of electrolyte and applied potential. Interestingly, the degradation efficiency was slightly reduced with the bias exceeding 1.0 V. This behavior was explained by the start of oxygen evolution, considered as an unwanted competing reaction. In addition to reducing the overall efficiency of anodic oxidation, oxygen generation can also adversely affect the access of organic molecules to the reactive sites on the electrode surface, resulting in lower degradation efficiency.

Li *et al.*^231^ systematically optimized the operating parameters of an MF_3_/BVO photoanode for the degradation of levofloxacin and studied the effect of the applied bias potentials of 0.4, 0.6, and 0.8 V *versus* Ag/AgCl. The degradation efficiency increased from 42% for 0.4 V to 78.72% at 0.8 V as shown in Fig. 14j, wherein higher bias potentials facilitated charge separation, increased photocurrent generation, and accelerated interfacial redox reactions. The authors noted, however, that degradation efficiency is not enough to determine the optimum operating conditions. Hence, the efficiency of the processes was evaluated using the energy per order (EE/O) metric. The smallest EE/O value of 0.0234 kWh m^−3^ min^−1^ was found for the condition at 0.6 V (Fig. 14k), which represents the most energy efficient condition. While this resulted in slightly higher pollutant removal, the increased energy demand was combined with an increased risk of electrode corrosion, catalyst degradation, and material leaching, making this process less attractive in practice. Thus, the bias potential of 0.6 V was selected as the optimal bias potential due to the consideration of degradation performance, energy consumption, and electrode stability.

Wang *et al.*^236^ investigated the influence of applied current on sulfamethoxazole mineralization during the PEF process using currents of 0.12, 0.24, 0.36, and 0.50 A (Fig. 14l). The TOC removal increased from 53% at 0.12 A to 66% and 80% at 0.24 and 0.36 A, respectively, demonstrating that increasing current promotes mineralization. This enhancement has been attributed to the greater electro generation of H_2_O_2_ at the cathode, and its electro generation is believed to lead to greater amounts of ˙OH *via* Fenton reactions. The current output directly affects the ˙OH production, which means that pollutant oxidation and mineralization are initially increased. However, when the applied current was increased to 0.50 A, TOC removal decreased to 67%, indicating a reduction in process efficiency. This decrease was attributed to several parasitic reactions that become significant at high current density. These include excessive oxidation of Fe^2+^ to Fe^3+^, which reduces the Fenton cycle activity, dimerization of ˙OH, *etc.* Such side reactions decrease the effective concentration of the oxidative species and lower the mineralization efficiency overall.

The effect of applied current density on ATP degradation in the PEC/E-PS system was also studied by Qin *et al.*^234^ The increase of current density from 1.67 to 5.00 mA cm^−2^ resulted in an increase of ATP removal from 56.87% to 96.77%, and the first-order rate constant increased from 0.0048 to 0.0196 min^−1^ as shown in Fig. 14m. This excellent degradation was attributed to the increased generation of hydroxyl radicals and other ROS at higher current densities. The enhancement of electron transfer processes will further increase current density and electrochemical activation of persulfate, which in turn will increase the concentration of sulfate radicals and improve oxidation kinetics. When the current density was raised to 6.67 mA cm^−2^, however, it was found that degradation efficiency decreased. This drop was largely due to the overproduction of oxygen on the anode surface. When high current densities are used, a significant amount of the electrical energy is consumed in oxidizing the water instead of degrading the pollutant, resulting in poor current utilization efficiency. Furthermore, the formation of oxygen bubbles may partially deactivate active sites and reduce mass transfer, which decreases the contact between the pollutants and the reactive species. These results suggest that there is a best current density for which parasitic reaction is minimized but radical production is maximized.

Overall, the studies show that potential and current density have a significant effect on the PEC-based degradation systems. An increase in these parameters typically results in greater charge separation, photocurrent generation, and production of reactive oxygen species, hence better degradation kinetics. Outside of this optimum range of operation, however, there are competing reactions that limit the benefits, including oxygen evolution, radical self-quenching, too much oxidant use, and higher energy needs. Hence, careful optimization of the electrical operating conditions is crucial to achieve the highest possible pollutant degradation with high current efficiency, energy economy, and lifetime of the electrodes.

### 6.3 Effect of oxidant concentration

In a hybrid PEC, the concentration of the auxiliary oxidant is one of the key parameters that directly affect the formation of strong radicals. At optimal concentration, oxidants accept electrons or react with photogenerated charges to produce radicals (SO_4_˙^−^, ˙OH, ^1^O_2_) that attack pollutants. In general, a higher oxidant concentration provides more reactant for activation, generating more SO_4_˙^−^ or ˙OH radicals as a result of the photo- or electro-activation process.^240^ This normally increases the rate of oxidation of pollutants up to the optimum level. Beyond this optimum, however, excess oxidants become counterproductive, and radicals begin to scavenge each other or form less-reactive species.^241^ For instance, excess PMS can exhibit quenching and/or radical recombination, reducing the net oxidizing power.^242^ Likewise, excess H_2_O_2_ can transform ˙OH into less reactive 
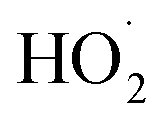
 radicals.^243^ It is assumed that the curve will have a characteristic bell shape, and the optimum oxidant dosage will yield the highest degradation rate and efficiency.

For instance, Dhawle *et al.*^235^ studied the effect of PMS concentration on the degradation of 17α-ethinyl estradiol (EE2) using TiO_2_/WO_3_ electrodes under the potential of 1 V (*vs.* Ag/AgCl) at natural pH in 0.05 M Na_2_SO_4_ (Fig. 14n). When there was no PMS, the system shows moderate removal efficiency of 47.9% in 60 min with an apparent rate constant of 0.01 min^−1^. When 10 mg per L PMS was added, the degradation efficiency jumped to 88.8% with a significant increase in rate constant (*k*) from 0.016 to 0.042 min^−1^. This improvement provides further evidence of the significant role PMS plays as an external oxidant in facilitating the generation of radicals and enhancing PEC rates. Additional adjustments of PMS concentration showed a clear dependency of degradation performance on the amount of oxidant used. At PMS concentrations of 5 mg L^−1^ and 2.5 mg L^−1^, the removal efficiency was reduced to 49.8% and 27.9%, respectively, which is a decrease of almost 1.7-fold and 3-fold, respectively in comparison with the optimum condition. This decrease is due to the lack of sufficient production of SO_4_˙^−^ and ˙OH, directly reducing the oxidation capacity of the system. While the addition of PMS improves the efficiency of PEC by providing extra reacting radical species, high PMS concentration can lead to scavenging of the radicals and the generation of less effective radical intermediates, thus decreasing the overall efficiency, the authors said. Thus, the optimum dosage of PMS is vital to control the generation of radicals and prevent undesirable side reactions.

Qin *et al.*^234^ also investigated how the ATP degraded in the presence of PS. Increasing the PS concentration from 100 to 550 mg L^−1^ significantly enhanced the degradation performance, with ATP removal increasing from 38.85% to 93.65%, while the corresponding first-order rate constant increased from 0.0028 to 0.0163 min^−1^ (Fig. 14o). The enhancement is due to the larger number of persulfate molecules available for activation, which leads to the growth of SO_4_˙^−^ and other ROS. Furthermore, higher concentrations of persulfate may lead to more production of H_2_O_2_ and radical formation, which can increase the oxidation kinetics. The positive impact of PS concentration was not, however, permanent. With a higher concentration (700 mg L^−1^), no significant improvement in ATP degradation was observed. The authors explained this phenomenon by the presence of side reactions between persulfate and radical species that lead to the consumption of active oxidants *via* self-quenching and scavenging processes. This is because such reactions decrease the available concentration of sulfate radicals to degrade pollutants. As a result, 550 mg L^−1^ was determined to be the most suitable persulfate concentration to ensure a good balance of radical generation and consumption.

Li *et al.*^231^ investigated the degradation of levofloxacin under the MF_3_/BVO photoanode with different PMS concentrations. The degradation efficiency increased from 60% to 78.72% when PMS concentration was increased from 0.05 to 0.1 mM (Fig. 14p), which indicates that a moderate concentration of PMS effectively promotes the generation of ROS, such as SO_4_˙^−^ and ˙OH radicals, and thus increases the degradation of the pollutant. However, further increasing the PMS concentration to 0.2 mM did not lead to any additional improvement in degradation performance. At higher PMS concentrations, this behavior indicates a more important role of radical–radical interactions, particularly SO_4_˙^−^ self-quenching reactions, leading to a decrease in the effective concentration of active oxidizing species. This interpretation is further confirmed by the trend observed in the kinetic rate constants, which show that the optimal dosage of PMS for this system is 0.1 mM.

**Fig. 15:**
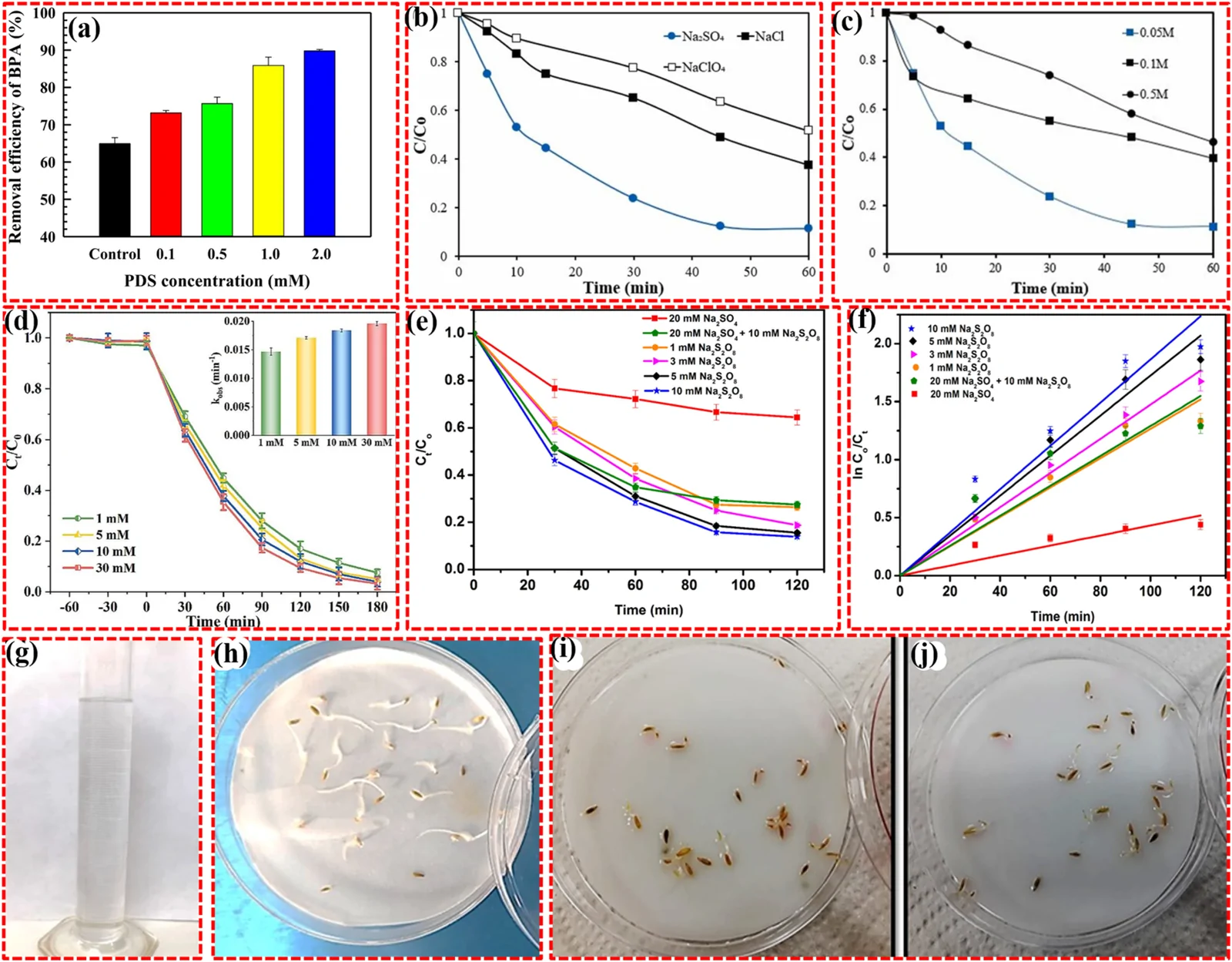
(a) Removal efficiency of bisphenol-A (BPA) under different PDS concentration ([BPA]_0_ = 10.0 µM; [(NH_4_)_2_SO_4_]_0_ = 0.1 M; potential bias = 1.0 V *vs.* Ag/AgCl; pH_*i*_ = 5.5). Reproduced with permission from ref. 131. Copyrights Elsevier, 2021. (b) Effect of 0.05 M of various electrolytes on the removal of EE2, (c) effect of Na_2_SO_4_ concentration. EE2 = 1 mg L^−1^; PMS = 10 mg L^−1^; 1 V. Reproduced with permission from ref. 235. Copyrights Elsevier, 2026. (d) Effect of electrolyte concentration on ATP degradation in the PEC system.^234^ (e) PEC degradation plots of sulfamethoxazole, (f) kinetics plots for degradation of sulfamethoxazole. Reproduced with permission from ref. 244. Copyrights Elsevier, 2021. Images of the (g) sample collection and measurement to perform the lettuce sativa germination biotoxicity test in (h) distilled water, (i) solution of 20 mg per L CIP and 0.05 mol per L Na_2_SO_4_, and (j) solution of 20 mg per L CIP and 0.05 mol per L Na_2_SO_4_ after 90 min of treatment with PECO. Reproduced with permission from ref. 245. Copyright Elsevier, 2022.

The effect of PDS concentration on PEC/PDS degradation of bisphenol A (BPA) was examined by Son *et al.*^131^ The results showed that increasing the initial PDS dosage progressively enhanced BPA removal, with the pseudo-first-order rate constant increasing from 1.02 h^−1^ (PEC alone) to 1.86 h^−1^ at 1.0 mM PDS. In accordance, the removal efficiency of BPA improved from 65.0% to 85.9% after 1 h (Fig. 15a), showing a high correlation between the oxidant concentration and degradation efficiency. No saturation was observed in the range of oxidant concentrations investigated, as some systems have shown saturation or inhibition at higher concentrations. The improved performance was explained by the higher production rate of SO_4_˙^−^ and ˙OH, the better charge separation in the PEC system, and the synergistic effect of the interaction between the photogenerated charge carriers and the PDS activation. These effects act in concert to provide more efficient oxidation pathways and faster degradation rates, making it very important that oxidant availability determines the performance of the PEC/PDS system.

### 6.4 Effects of electrolyte concentration

The supporting electrolyte affects hybrid PEC processes in terms of solution conductivity, ionic strength, and availability of secondary radicals. Increasing the concentration of electrolytes (*e.g.*, Na_2_SO_4_, NaCl) decreases the resistance of the solution and thus increases the photocurrent and charge transport efficiency, thereby enhancing the rate of ROS generation at the photoanode in principle.^246^ Some ions, however, can also react chemically, such as the radical formation of chlorine species, such as Cl˙, Cl_2_˙^−^, or HOCl, by the anodic or photolytic pathways.^247^ Similarly, carbonate/bicarbonate can consume ˙OH to generate CO_3_˙^−^, which is a longer-lived radical, thereby decreasing the amount of ˙OH.^248^ The net effect of an electrolyte is determined by its type and concentration; the effect of inert salts such as Na_2_SO_4_ is primarily on conductivity, while the effect of reactive salts such as NaCl and NaHCO_3_ is more complex.

For instance, Dhawle *et al.*,^235^ for example, explored the effects of different electrolytes and concentrations on the photoelectrochemical degradation of 17α-ethinyl estradiol (EE2). The results indicated that chloride-containing electrolytes harmed EE2 removal (Fig. 15b). Although NaClO_4_ is generally considered an inert electrolyte, its use led to a significant reduction in degradation efficiency. This reduction was attributed to the reduction of active catalytic sites, consumption of ˙OH, and the adverse effect of the highly acidic reaction medium, all of which reduced the availability of the reactive species for the oxidation of the pollutant. The influence of the electrolyte concentration was investigated by using Na_2_SO_4_ as a supporting electrolyte (Fig. 15c). As the concentration of Na_2_SO_4_ increases, EE2 degradation efficiency gradually drops, and 60.4% and 53.8% degradation efficiencies were achieved at 0.1 M and 0.5 M Na_2_SO_4_ concentrations, respectively. The decrease in performance was believed to be caused by the higher concentration of persulfate species formed by the sulfate radicals at higher sulfate concentrations. The conversion of highly reactive radicals to less reactive oxidants result in a reduction in the overall degradation efficiency, as persulfate has a lower oxidation potential than SO_4_˙^−^ and ˙OH radicals. The results showed that an increase in the electrolyte concentration has a negative effect on PEC performance despite the enhancement of the solution conductivity, suggesting that excess electrolyte concentrations may adversely affect PEC performance.

The effect of the supporting electrolyte on ATP degradation was also studied by Qin *et al.*^234^ with the use of Na_2_SO_4_. To investigate the practical application and economic feasibility, the electrolyte concentration was changed in the relatively low range of 1–30 mM. The ATP removal results (Fig. 15d) revealed that ATP removal gradually improved with increasing electrolyte concentration. This improvement was attributed to the increased solution conductivity, which allows for electron transfer, reduces ohmic resistance, and enhances charge transfer throughout the PEC system. Better conductivity leads to more efficient currents and promotes the production of reactive oxidative species, which contributes to the effectiveness of degradation. The improvement in removal was not very large, however, suggesting that conductivity was not the only rate-limiting factor. The authors also pointed out that extremely high electrolyte concentrations can have negative effects on degradation as Na^+^ and SO_4_^2−^ ions may compete with pollutant molecules to bind to the active catalytic sites. This competition can slow down the pollutant–electrode interactions and lower oxidation efficiency. Therefore, it is desirable to choose moderate electrolyte concentration to obtain the maximum conductivity benefits and to reduce competitive adsorption effects.

Koiki *et al.*^244^ reported a novel PEC approach for generating SO_4_˙^−^ from persulfate ions using an FTO–Cu_2_O photoanode for sulfamethoxazole degradation. In the presence of 20 mM Na_2_SO_4_, only 36% sulfamethoxazole degradation was achieved after 2 h (Fig. 15e and f). The efficiency of degradation was significantly enhanced to 72% when 10 mM Na_2_S_2_O_8_ and 20 mM Na_2_SO_4_ were added, highlighting the significant contribution of persulfate activation in enhancing the removal of pollutants through SO_4_˙^−^ generation. Interestingly, an even higher degradation efficiency of 84% was achieved using 10 mM Na_2_S_2_O_8_ without the addition of Na_2_SO_4_. The additive effect of Na_2_SO_4_ and Na_2_S_2_O_8_ also indicates that excess sulfate ions might react with the reactive species generated by persulfate or engage in radical scavenging reaction, which diminishes the effective concentration of sulfate radicals for oxidizing sulfamethoxazole. These results highlight the importance of optimizing sulfate and persulfate concentrations to maximize radical utilization and degradation efficiency in PEC systems.

### 6.5 Concluding remarks on parameter interactions

Overall, the operational parameters in hybrid PEC systems are strongly coupled, and their effects cannot be optimized independently. For example, pH simultaneously influences semiconductor surface charge, pollutant speciation, oxidant activation, and the balance between SO_4_˙^−^ and ˙OH pathways, while the applied bias controls charge separation and therefore interacts directly with light intensity, electrolyte composition, and oxidant concentration. Similarly, increasing oxidant or pollutant concentration can alter ROS availability, interfacial mass transfer, and scavenging reactions, whereas electrolyte composition affects conductivity, charge transport, and the formation or quenching of reactive species. Therefore, the optimum operating window represents a multidimensional balance among charge-transfer efficiency, ROS generation, pollutant adsorption, mass transport, and energy consumption rather than an optimum for any single parameter. Future studies should consequently employ systematic multivariable optimization and real-water validation to identify robust operating conditions for practical hybrid PEC applications.

## 7. Toxicity, environmental safety, and by-product assessment

Many highly reactive species are produced during the hybrid PEC process that breaks down the parent drug molecules. On the contrary, stepwise oxidation is more likely to generate transformation products (TPs) in the form of hydroxylated, dealkylated, or ring-opened fragments along with carboxylic acids prior to complete mineralization.^249,250^ Many of these intermediates may be more toxic or persistent than the parent compound or have biological activity. In hybrid PEC systems, the tracking of TPs takes place with the help of various analytical techniques. To explain degradation pathways, intermediate products are identified using liquid chromatography-mass spectrometry (LC-MS or LC-MS/MS), and reaction pathways are proposed. TOC or COD measurements can differentiate between transformation and true mineralization. Changes in solution toxicity are then monitored by standard bioassays. Common tests include the luminescent bacterium *Aliivibrio* (Vibrio) *fischeri* (Microtox) assay, which detects acute toxicity *via* inhibition of light emission, algal growth inhibition tests (*e.g.*, *Pseudokirchneriella subcapitata*) for effects on primary producers, immobilization or reproduction tests with *Daphnia magna* or other crustaceans for zooplankton sensitivity, and vertebrate assays such as zebrafish embryo toxicity for higher organisms.^251–253^ Terrestrial bioassays, such as growth or biomass tests, can also be used to test effluents for phytotoxicity.^254^ Ultimately, the goal of any hybrid PEC process is not just pollutant removal but also producing safe effluent. Environmental risk assessment considers other risks than those from acute toxicity, including persistence, bioaccumulation, and long-term ecological risks of remaining TPs. Even if an intermediate is moderately toxic in a lab assay, if it rapidly degrades or is taken up and metabolized by microorganisms, its actual risk may be low. Conversely, a seemingly benign organic acid that is extremely persistent could pose chronic risks. Evaluators compare the properties of identified TPs with known toxicological data. Computational tools (QSAR, quantitative structure–activity relationships) can predict, for example, estrogenic potential or fish toxicity of novel by-products.

For instance, Palomares-Reyna *et al.*^245^ evaluated the phytotoxicity of ciprofloxacin (CIP)-contaminated water before and after photoelectrocatalytic oxidation (PECO) treatment using *Lactuca sativa* seed germination and hypocotyl elongation tests (Fig. 15g–j). In the control experiment, 85% of the seeds germinated in distilled water, whereas only 13 out of 20 seeds germinated in untreated CIP-contaminated water, corresponding to a 23.5% reduction in germination. Following PECO treatment, seed germination slightly improved, with 15 seeds successfully germinating. A more pronounced improvement was observed in hypocotyl growth, which increased from 0.17 mm in untreated CIP-contaminated water to 0.33 mm after treatment. Despite this improvement, the value remained significantly lower than those observed in pure distilled water (0.89 mm). The authors proposed that possible acidic degradation residues remaining after CIP may still inhibit plant growth. The lower pH of the treated solution can affect nutrient availability and promote microbial growth, ultimately interfering with water and nutrient uptake by plant tissues. The results suggest that the toxicity of the parent pharmaceutical was decreased after PECO treatment; however, complete detoxification had not been achieved because potentially harmful transformation products remained.

**Fig. 16:**
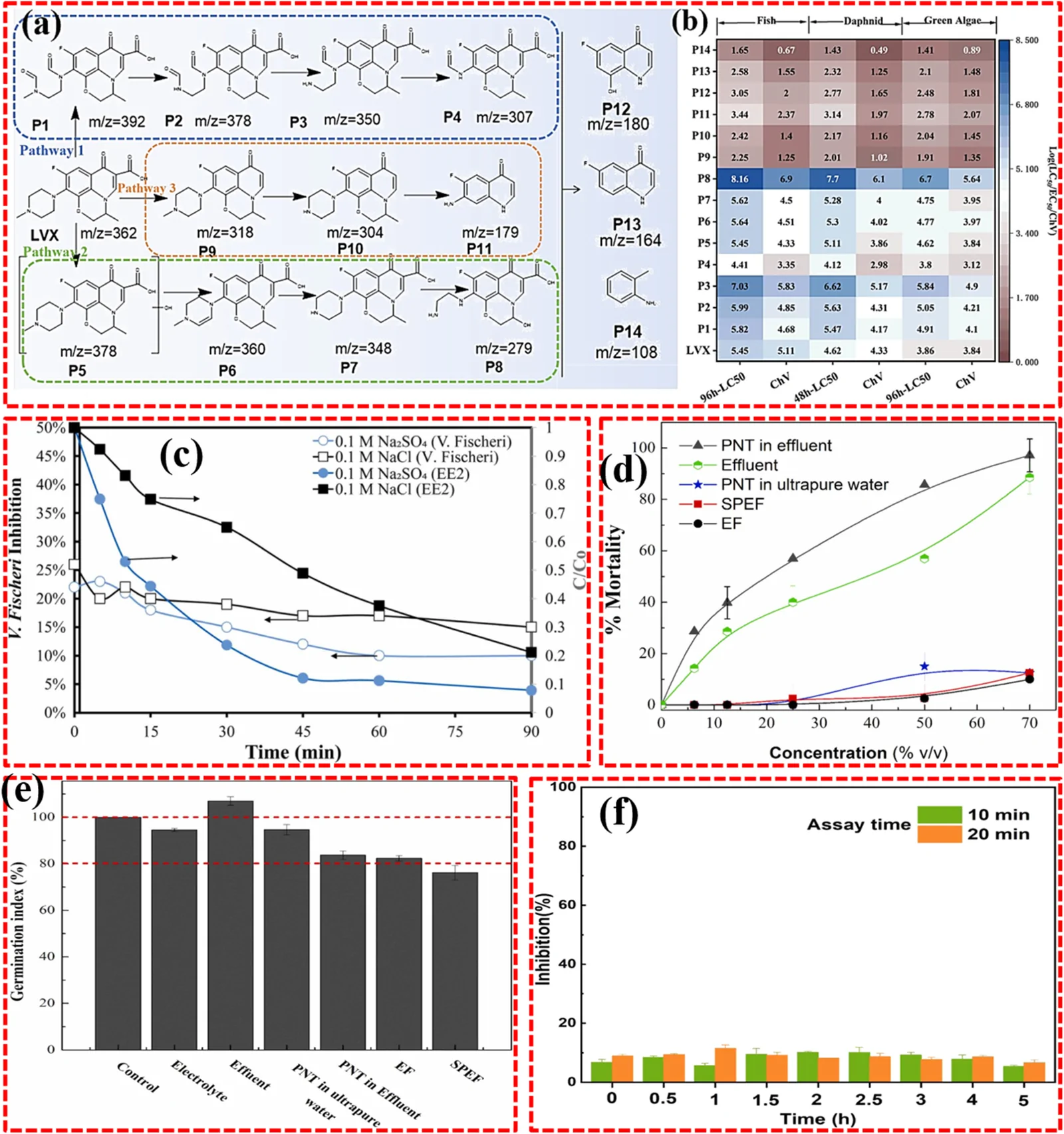
(a) Proposed degradation pathways of LVX; (b) estimated acute and chronic toxicity (log 10 values) of LVX and its intermediates toward fish, daphnia, and green algae using ECOSAR. Reproduced with permission from ref. 231. Copyrights Elsevier, 2026. (c) Inhibition of *V. fischeri* and associated EE2 removal. Na_2_SO_4_ or NaCl = 0.05 M; EE2 = 1 mg L^−1^; PMS = 10 mg L^−1^; 1 V. Reproduced with permission from ref. 235. Copyrights Elsevier, 2023. (d) Toxicity to *Artemia salina*, determined as a relationship between the number of dead individuals and the dilution employed, (e) toxicity to *Lactuca sativa*, determined by germination index (GI%) for the treatment of effluents carried out under optimum EF and SPEF mineralization conditions. Reproduced with permission from ref. 255. Copyrights Elsevier, 2026. (f) Results of bacterial inhibition. Reproduced from ref. 140. Which is open access and permits unrestricted use of materials under the terms of Creative Commons CC-BY.

Li *et al.*^231^ evaluated the environmental safety of the PEC/PMS degradation of levofloxacin (LVX) by the ECOSAR toxicity prediction model based on a type-II [Mo_72_Fe_30_]/BiVO_4_ photoanode. The toxicities of LVX and its degradation intermediates were assessed for acute and chronic exposure to fish, daphnia, and algae using LC_50_, EC_50_, and chronic toxicity values (Fig. 16a and b). The results showed that most degradation products were not toxic to the organisms used in the test, and that only certain intermediates, such as P9 and P14, had measurable toxicity. For chronic toxicity, several intermediates were found to be toxic or harmful, particularly when tested against the sensitive species *Daphnia* and algae. Notably, all the products of transformation identified were not marked as highly toxic in the acute or chronic toxicity analyses. The results obtained in this study confirm the ability of the PEC/PMS process to break down LVX and reduce the generation of highly hazardous by-products, making it a promising, environmentally friendly treatment technology.

Dhawle *et al.*^235^ studied toxicity development during the degradation of the synthetic estrogen 17α-ethinyl estradiol under PEC/PMS conditions using the bioindicator Vibrio fischeri. The study highlighted that high-performance advanced oxidation processes can effectively eliminate the target contaminant, but the possibility of total mineralization is limited, leading to the generation of transformation products, which might cause the same or greater toxicity. The results indicated that the reduction of toxicity was not directly proportional to the reduction of 17α-ethinyl estradiol, and that some oxidation intermediates were still toxic to *V. fischeri* (Fig. 16c). However, the toxicity of the untreated solution was not high (20–25%). Interestingly, experiments were carried out with NaCl as the supporting electrolyte, and no increase in toxicity was observed, which means that toxic organochlorinated by-products were not formed in large amounts under the investigated conditions. The authors noted that the toxicity data used on a single species may not accurately reflect environmental hazards and suggested that additional research be conducted using several bioassays, including estrogenicity tests, to evaluate treated effluents more completely.

Cardoso *et al.*^255^ investigated the toxicity of the wastewater before and after treatment by electro-Fenton (EF) and solar photoelectro-Fenton (SPEF) processes using two bioindicators: *Artemia salina* and *Lactuca sativa*. The untreated secondary effluent was found to have high acute toxicity, and further increased with the inclusion of PNT, with toxicity unit (TU) values of 1.4 ± 1.3 and 2.5 ± 1.2, respectively (Fig. 16d). Optimum treatment resulted in significant decreases in toxicity for both EF and SPEF. The EF process effectively reduced toxicity by 89.6% with a TU value of 0.26 ± 1.4 (non-toxic effluent, TU < 0.4). The sample treated with SPEF, however, showed 78.0% reduction in toxicity with a TU value of 0.55 ± 0.06, reflecting slight acute toxicity. The authors believe that the mineralization rate during treatment and the high sensitivity of the test organism are responsible for this residual toxicity. Further assessment was made of the phytotoxicity of treated effluents by conducting Lactuca sativa germination tests. Seed germination was not significantly affected by either the raw effluent or the PNT-containing solutions, showing germination index (GI) of 107.0%, 83.7%, and 94.7% for the raw effluent, PNT-enriched effluent, and PNT solution, respectively (Fig. 16e). The result revealed that the effluent treated with EF had a GI value of 82.3%, while the effluent treated with SPE had a lower GI value of 76.2% after treatment showed that there is no phytotoxic effect on the seeds of EF treated effluent, whereas the seeds of the SPE-treated effluent showed mild inhibition on seed germination. This inhibition was felt to be caused by the formation of oxidation by-products during treatment. The overall study results showed that both EF and SPEF are effective methods for reducing the toxicity of treated wastewater, but the results indicated that a high degree of mineralization is needed to ensure that the transformation products of the treated wastewater are not significant environmental hazards.

Zou *et al.*^140^ assessed the ecotoxicity of water samples before and after treatment by the PPBEC process using the luminescence inhibition assay of *Vibrio fischeri*. The results indicated that the bacterial luminescence inhibition increases slightly at the beginning of the treatment and then decreases gradually, while the values are almost the same during the whole treatment process, ranging around 10% (Fig. 16f). This means that degradation of pharmaceuticals was not associated with the production of significant amounts of highly toxic intermediates and that the overall toxicity of the treated water was decreased. Some advanced oxidation processes like ozonation and UV treatments result in more ecotoxic transformation products in the degradation process, which is not observed in this case. The lower toxicity in the PPBEC system was attributed to the effective oxidation and subsequent transformation of the intermediate products, which were not allowed to accumulate in the treated effluent. The results obtained in the present assessment indicate that the PPBEC process is capable of efficiently removing contaminants with a low ecotoxicological risk; thus, it is a potential technology for environmentally friendly wastewater treatment.

## 8. Real wastewater treatment and challenges

Although most PEC hybrid systems have demonstrated excellent performance in synthetic aqueous solutions, their practical application ultimately depends on their effectiveness in real wastewater. Unlike synthetic matrices, real wastewater contains a complex mixture of natural organic matter (NOM), inorganic ions (*e.g.*, HCO_3_^−^, Cl^−^, NO_3_^−^, and SO_4_^2−^), suspended solids, dissolved organic carbon, and microorganisms, all of which can significantly influence degradation pathways.^256–258^ These compete with pharmaceutical molecules for the adsorption to the active sites of the catalysts and they readily scavenge ROS, which reduces the efficiency of oxidant utilization. Furthermore, suspended particles decrease light transmittance and constant exposure to the components of wastewater causes catalyst fouling and electrode deactivation.^259^ Therefore, assessing PEC hybrid systems under real wastewater is vital to evaluate their stability, selectivity and feasibility for practical water treatment.

Dong *et al.*^260^ evaluated the efficiency of a Pt@CeO_2_@MoS_2_-assisted PEC/PMS system, using municipal sewage samples from the sewage treatment plants of Shatin and Sai Kung. The wastewater matrix was complex, but the integrated PEC/PMS process was able to completely mineralize TOC in 20 min, while PEC and PMS alone had significantly lower mineralization efficiencies. The remarkable enhancement can be attributed to the synergistic interaction between photoelectrocatalysis and PMS activation, which continuously generated abundant SO_4_˙^−^ and ˙OH. These highly active species not only mineralize pharmaceutical compounds, but also natural organic matter that is more resistant in sewage. The progressive reduction of protein-like and humic-like substances confirmed by excitation–emission matrix (EEM) analysis suggests a significant degradation of dissolved organic matter that often hinders the realization of AOP. The degradation efficiency of the catalyst was kept above 95% in repeated reactions, but the reaction kinetics were gradually reduced. The reduction was primarily attributed to the natural organic matter adsorption on the surface of the catalyst, which resulted in the active sites' blockage, charge transfer restriction, and limitation of reactive species-pollutants interactions. The results presented here have shown that the treatment performance of the PEC/PMS system is excellent for real wastewater. However, the fouling of the catalyst by matrix components is still a significant drawback for long-term use.

Similarly, Zheng *et al.*^124^ demonstrated the practical applicability of a PMS-assisted PEC system employing a 1T/2H-MoS_2_@C/CC photoanode for norfloxacin removal from drinking water. In the presence of PMS, 2 ppm norfloxacin was completely degraded within 30 min at an applied potential of 1.5 V. Most importantly, degradation efficiency was not significantly affected in various water matrices, revealing that there was a high tolerance for interference from coexisting ions and dissolved organic matter. This superior performance can be attributed to the unique 1T-rich MoS_2_ structure, which provides high electrical conductivity, abundant catalytically active sites, and rapid charge separation, thereby sustaining efficient PMS activation even in complex water environments. In addition, multiple different reactive oxygen species were generated simultaneously, such as SO_4_˙^−^, ˙OH, O_2_˙^−^, and ^1^O_2,_ thereby balancing the scavenging of individual species. The photoanode proved to be very stable in operation, maintaining 87.6% of its initial activity ground water 5 consecutive cycles with almost no Mo release (0.05 mg L^−1^), and thus ensuring its stability and not causing any significant secondary metal contamination. The results show that the design of highly conductive and chemically stable photoelectrodes, which can produce more than one type of reactive species, is an effective method to reduce the matrix interference in real water treatment.

Although all these promising developments, several barriers still hinder the widespread application of the PEC hybrid system for real wastewater treatment. The continuous consumption of ROS by natural organic matter and inorganic ions *via* radical scavenging reactions decreases the efficiency of ROS utilization and increases chemical consumption.^261^ The suspended solids and dissolved organic substances reduce the light transmission and cause catalyst fouling that causes the progressively reduced activity of photo electrodes.^262,263^ Moreover, long-term operation can lead to corrosion of electrodes, deactivation of the catalysts, and loss of active components, thereby reducing the lifetime of the system.^264^ High energy consumption and operational costs are further driven by the need for external oxidants like PMS or PDS to be continuously supplied and the use of electric bias.^259,265^ Notably, although high pharmaceutical removal has been often reported, the complete mineralization process may be challenging, resulting in persistence of transformation products that may present long-term ecological risks. Future work should therefore focus on continuous-flow pilot-scale research with real wastewater, on designing fouling-resistant and self-cleaning photoelectrodes, on enhancing the efficiency of the use of these oxidants, on combining renewable energy sources to reduce the energy requirement, and on detailed toxicity and mineralization studies to ensure that the process not only removes contaminants efficiently but remains environmentally safe.

## 9. Electrical energy per order (EEO) and techno-economic feasibility

Energy demand is one of the principal barriers separating hybrid PEC-AOPs from full-scale deployment, yet energy consumption is rarely quantified in a form that allows systematic comparison across studies.^264^ Electrical Energy per Order (EEO) defined as the electrical energy required to reduce the concentration of a target pollutant by one order of magnitude in one cubic meter of water has become the standard figure of merit for benchmarking the energy efficiency of AOPs since its introduction in 2001, because it normalizes energy input against both treated volume and degradation extent, unlike simple percentage energy-reduction claims.^266^ The IUPAC technical report on EEO reporting explicitly cautions that the metric is strongly dependent on reactor geometry, light-source efficiency, pollutant identity and concentration, and oxidant dose, and recommends that these parameters always be disclosed alongside EEO values to allow valid cross-study benchmarking.^266^ Conventional UV/H_2_O_2_ AOPs treating trace organic contaminants in real water matrices typically report EEO values in the range of ≈0.06–3 kWh m^−3^ per order at pilot to full scale,^266–268^ while bench-scale UV/H_2_O_2_ or O_3_-based systems applied specifically to pharmaceuticals and other emerging contaminants have reported considerably higher values, for example 4.6 kWh m^−3^ for ozone/peroxymonosulfate degradation of the artificial sweetener acesulfame and up to several tens of kWh m^−3^ per order for less optimized UV/H_2_O_2_ configurations.^269^ Electrochemically activated PMS systems, which are mechanistically closer to the PEC/PMS hybrids discussed throughout this review, have reported an EEO of 2.12 kWh m^−3^ per order for sulfamethoxazole degradation using a carbon-cloth anode,^270^ while photocatalytic TiO_2_ systems operating without added oxidant have achieved EEO values between 0.96 and 4.07 kWh m^−3^ per order for acetaminophen, sulfamethoxazole, and carbamazepine in natural waters.^267^

Within the hybrid PEC-AOP literature reviewed in the preceding sections, only a small number of studies reports EEO explicitly, and the reported values are strikingly favorable by comparison. Liu *et al.*^137^ achieved complete removal of 10 mg L^−1^ carbamazepine within 30 min using a TiO_2_/nickel-foam photoanode coupled to a natural air-diffusion cathode in a PEC/photoelectro-Fenton configuration, operating at only 2 mA applied current (1.7 V cell potential) and reporting an EEO of 0.005 kWh m^−3^ per order roughly two to three orders of magnitude below the benchmark values described above. Li *et al.*^231^ systematically varied the applied bias (0.4–0.8 V) for levofloxacin degradation over an (Mo_72_Fe_30_)/BiVO_4_ PEC/PMS photoanode and found that, although degradation efficiency continued to rise with bias up to 0.8 V, the minimum EEO of 0.0234 kWh m^−3^ min^−1^ occurred at the intermediate bias of 0.6 V, leading the authors to select 0.6 V as the optimal operating condition once energy efficiency and electrode-stability considerations were weighed against raw removal efficiency. In a related but distinct approach, Zou *et al.*^140^ coupled persulfate activation with a photo-bioelectrochemical cell (PPBEC) for the removal of carbamazepine and clofibric acid from real secondary wastewater effluent, reporting a 93% reduction in overall energy consumption relative to the individual UV/PS and PBEC processes operated alone, although this study reported only a relative energy saving rather than an absolute EEO value.

While these EEO figures suggest that hybrid PEC-AOP systems can, under bench-scale conditions, substantially outperform conventional AOP benchmarks in terms of raw electrochemical energy efficiency. Several critical caveats limit the interpretation of this comparison as evidence of true techno-economic feasibility. First, none of the hybrid PEC studies discussed in this review report EEO under the standardized conditions recommended by the IUPAC protocol, and the values that are reported are not even expressed in consistent units which precludes direct, like-for-like benchmarking. Second, every reported EEO value originates from small-volume, single-pollutant, bench-scale batch reactors; none of the studies account for the auxiliary energy demand of pumping, aeration, or electrode/photocatalyst regeneration expected at pilot or full scale, nor for the parasitic resistive losses that typically accompany the scale-up of biased photoelectrodes. Third, and most importantly for practical feasibility, none of the reviewed hybrid PEC-AOP studies report capital expenditure, electrode or catalyst fabrication and replacement cost, or the recurring reagent cost of the PMS/PDS/H_2_O_2_ oxidant costs that frequently dominate the overall operating expenditure of oxidant-assisted AOPs and that a low EEO value alone cannot capture. Consequently, the very low EEO values reported for hybrid PEC systems such as should be interpreted as evidence of favorable electrochemical energy efficiency under specific, non-standardized bench-scale conditions, rather than as proof of overall economic viability at scale. Future studies on hybrid PEC-AOP systems should therefore (i) report EEO according to the standardized IUPAC protocol and in consistent units to enable direct benchmarking against conventional AOPs and (ii) accompany EEO reporting with a preliminary techno-economic analysis incorporating electrode/catalyst cost and lifetime and oxidant consumption cost, validated wherever possible at pilot scale using real wastewater. Energy efficiency, life-cycle assessment, and techno-economic feasibility are critical considerations for translating hybrid PEC systems from laboratory demonstrations to practical wastewater treatment. However, the available literature remains dominated by laboratory-scale investigations, and systematic LCA and TEA studies specifically addressing hybrid PEC systems are still very limited. Most reported studies evaluate energy performance using electrical energy per order (EEO), electrical energy per unit mass (EEM), or specific energy consumption (SEC), rather than complete life-cycle or cost assessments. Reported energy requirements vary substantially with reactor configuration, pollutant concentration, water matrix, irradiation source, applied potential, and treatment endpoint. For example, a PEC/PEF system for carbamazepine removal reported an exceptionally low EEO of 0.005 kWh m^−3^ per order,^137^ whereas PEC treatment of real industrial wastewater has been reported at approximately 274–297 kWh m^−3^ per order,^271–273^ illustrating the strong influence of matrix complexity and reactor operating conditions. Solar photoelectro-Fenton treatment of a pharmaceutical mixture has also demonstrated an energy consumption of 7.15 kWh m^−3^, while specific energy requirements of 11.73–39.32 kWh kg^−1^ pollutant have been reported for individual pharmaceuticals.^274^ These differences demonstrate that energy performance cannot be compared solely on the basis of removal efficiency and that standardized reporting of EEO, EEM/SEC, irradiation energy, applied electrical energy, and mineralization is necessary. Importantly, comprehensive LCA and TEA incorporating electrode and catalyst manufacturing, oxidant consumption, electricity demand, reactor construction, catalyst/electrode replacement, sludge or by-product management, and treatment lifetime are largely lacking for hybrid PEC systems. Consequently, future studies should combine standardized energy metrics with cradle-to-gate or cradle-to-grave LCA and TEA under realistic continuous-flow and real-water conditions to determine whether the reported laboratory-scale improvements translate into genuine environmental and economic advantages.

## 10. Conclusion

This review demonstrates that hybrid photoelectrocatalytic (PEC) advanced oxidation processes (AOPs) provide a versatile strategy for addressing the persistent and biologically active pharmaceutical micropollutants that remain inadequately treated by conventional wastewater processes. The central advantage of PEC originates from the integration of semiconductor photochemistry with an external electrical bias, which promotes interfacial charge separation, suppresses electron–hole recombination, and increases the availability of oxidative charge carriers. Coupling PEC with complementary oxidation or biological processes further establishes synergistic reaction pathways that can substantially improve pharmaceutical degradation and mineralization. Among the hybrid configurations evaluated, PEC/PMS and PEC/PDS systems are particularly effective because persulfate oxidants act as electron acceptors while generating multiple reactive species, including SO_4_˙^−^, ˙OH, O_2_˙^−^, and ^1^O_2_. The reviewed studies demonstrate pharmaceutical removal efficiencies commonly approaching 90–100%, with representative systems achieving 98% sulfamethoxazole removal, complete norfloxacin degradation, and enhanced degradation kinetics compared with individual processes. PEC/H_2_O_2_ and photoelectro-Fenton systems predominantly promote ˙OH generation and provide an additional route for rapid oxidation. For example, 95% tetracycline removal was achieved within 90 min in a PEC/H_2_O_2_ system. Ozonation-assisted PEC similarly provides strong oxidative capacity, while bio-PEC configurations offer an important pathway for coupling chemical oxidation with biological treatment and reducing overall energy requirements. Importantly, high parent-compound removal does not necessarily indicate complete treatment. The reviewed evidence shows that mineralization, transformation-product formation, and toxicity must be considered alongside pharmaceutical disappearance. In real municipal wastewater, a PEC/PMS system completely mineralized TOC within 20 min and maintained degradation efficiency above 95% during repeated operation; however, progressive kinetic decline was associated with natural organic matter adsorption and catalyst fouling. Another PMS-assisted PEC system completely degraded 2 mg per L norfloxacin within 30 min at 1.5 V and retained 87.6% of its initial activity after five cycles, with only 0.05 mg per L Mo release. These findings demonstrate both the practical potential and the persistent influence of wastewater matrix effects. Mechanistically, the review highlights that radical pathways provide high oxidation strength but are vulnerable to scavenging by natural organic matter and inorganic ions, whereas non-radical pathways involving ^1^O_2_ and high-valent metal-oxo species can provide greater selectivity toward electron-rich pharmaceutical structures. Consequently, future system design should move beyond maximizing ROS production toward controlled ROS utilization, selective oxidation, and efficient mineralization. Despite substantial laboratory-scale progress, catalyst/electrode fouling, corrosion, catalyst leaching, oxidant consumption, mass-transfer limitations, incomplete mineralization, transformation-product toxicity, and electrical energy demand remain major obstacles to scale-up. Therefore, future investigations should prioritize continuous-flow operation with real wastewater, long-term electrode stability, standardized energy and toxicity metrics, mineralization assessment, oxidant utilization efficiency, and techno-economic evaluation. Overall, hybrid PEC-AOPs represent a promising transition from laboratory-scale pollutant degradation toward practical pharmaceutical wastewater treatment, but their industrial deployment will depend on simultaneously achieving high removal, deep mineralization, low toxicity, operational stability, and energy-efficient treatment.

## 11. Future prospective

• Design of visibly-active nano-architectures and phase engineering: improved structural materials such as MXenes, covalent organic frameworks (COFs), and single-atom catalyst cover layers should be integrated into future photoelectrodes so that they can work in the visible light spectrum instead of wide-bandgap semiconductors. Engineering mixed-phase heterojunctions (*e.g.*, IT/2H phase-engineered structures) or S-scheme systems could help to reduce onset potential and optimize band bending, thereby maximizing the use of solar energy.

• Transitioning from indiscriminate radical attacks to selective non-radical paths: future systems should be designed to promote selective non-radical degradation of the natural organic matter and inorganic ions with severe radical-scavenging properties present in real wastewater. This can be accomplished through the synthesis of vacancy-rich catalysts or the synthesis of counter-electrodes made of a carbon matrix with intentionally bound carbonyl groups that would directly convert PMS or O_3_ into singlet oxygen (^1^O_2_) or high-valent metal-oxo species, thus selectively directing the oxidation potential towards the targeted pharmaceutical rings.

• Upscaling continuous-flow pilot plants and optimization of fluid dynamics: the current literature is heavily dominated by batch-scale, single-pollutant laboratory setups that fail to simulate realistic operational dynamics. Future milestones must focus on constructing continuous-flow, multi-stage pilot installations, utilizing advanced response surface methodology and computational fluid dynamics (CFD) to optimize reactor geometry, light transmission paths, and hydraulic retention times.

• Mitigating photoelectrode fouling and development of self-cleaning substrates: a major barrier to long-term industrial application is the rapid loss of photoelectrode performance caused by surface poisoning, inorganic scaling, and organic macromolecule fouling. Future designs should integrate multi-functional, conductive, and self-cleaning catalytic membranes that leverage localized surface acidity and continuous radical generation to repel foulants and maintain stable target photocurrents actively.

• Ecotoxicological profiling and complete transformation pathway elucidation: the disappearance of the parent drug is also assessed using standard chromatography procedures, which need to be complemented by rigorous multi-trophic ecotoxicological evaluations. Systematically mapping out intermediate transformation pathways using high-resolution mass spectrometry (LC-MS/MS), followed by bioassays, would be a good next step to enable hybrid PEC treatments to be designed to reduce biological activity, while avoiding the inadvertent release of more toxic or persistent by-products.

• Integration with renewable energy sources for self-sustaining treatment loops: the costs associated with the use of external chemical feed and electrical power are significant and continue after installation. Therefore, engineering for the future should focus on the integration of PEC systems directly with green hydrogen-producing cathodes or a closed-loop, self-sustaining microbial fuel cell (MFC), which would create a solar and biochemical energy-driven bias-free treatment system.

## Conflicts of interest

The authors declare that they have no conflict of interest.

## Data Availability

No new data was generated or analyzed in this study.

## References

[cit1] Saravanan A. (2021). *et al.*, Effective water/wastewater treatment methodologies for toxic pollutants removal: Processes and applications towards sustainable development. Chemosphere.

[cit2] Oladipo A. A., Ahmad M. (2025). Energy-Efficient Ion Recovery from Water Using Electro-Driven Membranes: A Comprehensive Critical Review. Water.

[cit3] Kyere-Yeboah K., Bique I. K., Qiao X.-C. (2023). Advances of non-thermal plasma discharge technology in degrading recalcitrant wastewater pollutants. A comprehensive review. Chemosphere.

[cit4] khan S. (2026). *et al.*, Radical-Driven UV-Light-Responsive MnO2–Biochar Hybrid From Calotropis procera for Complete Degradation of Paracetamol Pollutant. J. Nanotechnol..

[cit5] Hama Aziz K. H. (2025). *et al.*, Biochar-based catalysts: an efficient and sustainable approach for water remediation from organic pollutants via advanced oxidation processes. J. Environ. Manage..

[cit6] Rahman S. U. (2026). *et al.*, Engineering MXene-based platforms for electrocatalytic CO2 and N2 reduction reactions; synthesis, modification strategies, and mechanistic insights. J. Environ. Chem. Eng..

[cit7] Khan S. (2025). *et al.*, Cutting-edge research progress in synthesis and applications of MXene-based nanocomposites for water purification: A comprehensive review. J. Environ. Chem. Eng..

[cit8] Hama Aziz K. H. (2024). *et al.*, Biochar as green adsorbents for pharmaceutical pollution in aquatic environments: A review. Desalination.

[cit9] Hama Aziz K. H. (2025). *et al.*, Pharmaceutical pollution in the aquatic environment: advanced oxidation processes as efficient treatment approaches: a review. Mater. Adv..

[cit10] Nyaga M. N. , Nyagah D. M., and Njagi A., Pharmaceutical Waste: Overview, Management, and Impact of Improper Disposal, 2020

[cit11] Wu M. , et al., Dosed without Prescription: Preventing Pharmaceutical, Natural Resources Defense Council (NRDC), Inc, 2009

[cit12] Khan S. (2026). *et al.*, MXene-based membranes for advanced desalination: Properties, engineering strategies, and emerging applications. Desalination.

[cit13] Aziz K. H. H., Mustafa F. S., Hama S. (2025). Pharmaceutical removal from aquatic environments using multifunctional metal-organic frameworks (MOFs) materials for adsorption and degradation processes: a review. Coord. Chem. Rev..

[cit14] Hama Aziz K. H. (2026). Metal-organic frameworks (MOFs) based adsorbents for efficient removal of antibiotic pollutants from water. Front. Mater..

[cit15] O'eill J. , Antimicrobials in agriculture and the environment: reducing unnecessary use and waste. The review on antimicrobial resistance, London:The Review on Antimicrobial Resistance, 2015, p. 2103774

[cit16] Sardar M. (2026). *et al.*, Fluorescence-based detection of antibiotics in aquatic environments using carbon nanodots: a review. RSC Sustainability.

[cit17] Wilkinson J. L. (2022). *et al.*, Pharmaceutical pollution of the world's rivers. Proc. Natl. Acad. Sci..

[cit18] Chaber-Jarlachowicz P., Gworek B., Kalinowski R. (2025). Removal efficiency of pharmaceuticals during the wastewater treatment process: Emission and environmental risk assessment. PLoS One.

[cit19] Mohd Hanafiah Z. (2025). *et al.*, Global pharmaceutical pollution in waterways: Insights from sewage treatment point sources. Emerging Contam..

[cit20] Hama Aziz K. H. (2023). Heterogeneous catalytic activation of peroxydisulfate toward degradation of pharmaceuticals diclofenac and ibuprofen using scrap printed circuit board. RSC Adv..

[cit21] Oladoye P. O. (2024). *et al.*, Advancements in adsorption and photodegradation technologies for Rhodamine B dye wastewater treatment: fundamentals, applications, and future directions. Green Chem. Eng..

[cit22] Abdalla S. A. (2025). *et al.*, Sustainable and efficient persulfate activation by pristine pumpkin seed pomace biochar: a low-energy regeneration strategy and singlet oxygen-dominated pathways. RSC Adv..

[cit23] Ramzanipour M. M., Mousavi S., Vajargah M. F. (2023). Overview of Municipal Wastewater and Sludge Treatment Process. Ecol. Conserv. Sci. Open Access 2.

[cit24] khan S. (2025). *et al.*, Smart and Sustainable Microplastic Removal: Hybrid Systems, Bio-Inspired Technologies, Real-Time Sensing, and Policy Integration. Water, Air, Soil Pollut..

[cit25] Kashif M. (2026). *et al.*, Synthesis Strategies and Multifaceted Applications of Molybdenum-Based Nanomaterials: A Comprehensive Review. Scientifica.

[cit26] Deblonde T., Cossu-Leguille C., Hartemann P. (2011). Emerging pollutants in wastewater: A review of the literature. Int. J. Hyg. Environ. Health.

[cit27] Comber S. (2018). *et al.*, Active pharmaceutical ingredients entering the aquatic environment from wastewater treatment works: A cause for concern?. Sci. Total Environ..

[cit28] Wang Y. (2018). *et al.*, Monitoring, mass balance and fate of pharmaceuticals and personal care products in seven wastewater treatment plants in Xiamen City, China. J. Hazard. Mater..

[cit29] Verlicchi P., Al Aukidy M., Zambello E. (2012). Occurrence of pharmaceutical compounds in urban wastewater: removal, mass load and environmental risk after a secondary treatment—a review. Sci. Total Environ..

[cit30] Khan S. (2026). *et al.*, Bias-Free Catalytic Photoelectrochemical Degradation of Congo Red Using a Boron–Iron Oxide Coated Electrode: A Green and Sustainable. Catal. Surv. Asia.

[cit31] Kashif M. (2025). *et al.*, Bridging MOFs and MXenes: from synthesis to environmental and energy technologies. Phys. Chem. Chem. Phys..

[cit32] Oladipo A. A. (2026). Spinel ferrite–based advanced oxidation processes for recalcitrant and emerging contaminants: Mechanistic insights, structure–property relationships, and hybrid AOP strategies. J. Environ. Chem. Eng..

[cit33] Aziz K. H. H. (2025). *et al.*, Pharmaceutical pollution in the aquatic environment: advanced oxidation processes as efficient treatment approaches: a review. Mater. Adv..

[cit34] Madhavan J. (2019). *et al.*, Hybrid advanced oxidation processes involving ultrasound: an overview. Molecules.

[cit35] Oladipo A. A. (2026). The double-edged nanoparticle: remediation benefits vs. mechanistic toxicity risks in aquatic systems. Environ. Sci.: Nano.

[cit36] Khan S. (2024). *et al.*, Photocatalytic dye degradation from textile wastewater: a review. ACS Omega.

[cit37] Rahman S. U. (2026). *et al.*, Synthesis, Properties, and Photocatalytic Hydrogen Production of G-C3N4/MXene Composites: A Review. Clean: Soil, Air, Water.

[cit38] Amini-Seresht N. (2025). *et al.*, Hybrid of photoelectrocatalysis and membrane processes as an efficient combination in pollutant removal from wastewaters: A review. Sep. Purif. Technol..

[cit39] Li X. (2022). *et al.*, Challenges of photocatalysis and their coping strategies. Chem Catal..

[cit40] Huang Y. (2024). *et al.*, All-inorganic lead halide perovskites for photocatalysis: a review. RSC Adv..

[cit41] Jia W. (2024). *et al.*, A well-designed hierarchical Bi 19 S 27 Br 3 nanorods@ SnIn 4 S 8 nanosheet core–shell S-scheme heterostructure for robust photothermal-assisted photocatalytic CO 2 reduction. J. Mater. Chem. A.

[cit42] Cowan A. J., Durrant J. R. (2013). Long-lived charge separated states in nanostructured semiconductor photoelectrodes for the production of solar fuels. Chem. Soc. Rev..

[cit43] Liu N. (2023). *et al.*, Modulation of photogenerated holes for enhanced photoelectrocatalytic performance. Microstructures.

[cit44] Khan S. (2026). *et al.*, Photoelectrocatalytic advanced oxidation of dyes and pharmaceuticals: a comprehensive review of electrode materials, reactor designs, mechanisms and influencing parameters. Environ. Sci.: Water Res. Technol..

[cit45] Zheng Z. , A Multifunctional Photoelectrochemical System for Coupled Sewage Treatment with H_2_ Evolution Using the BiVo_4_-Based Photoanode, Hong Kong University of Science and Technology, Hong Kong, 2022

[cit46] Hu Z. (2023). *et al.*, Efficient bias-free degradation of sulfamethazine by TiO2 nanoneedle arrays photoanode and Co3O4 photocathode system under LED-light irradiation. Catalysts.

[cit47] Ruofan X. (2025). *et al.*, Engineering photocatalytic membrane reactors for sustainable energy and environmental applications. Catalysts.

[cit48] Kaushik R. (2024). *et al.*, Design and investigation of photoelectrochemical water treatment using self-standing Fe3O4/NiCo2O4 photoanode: In-situ H2O2 generation and fenton-like activation. Chem. Eng. J..

[cit49] Ahmed H. R., Ealias A. M., George G. (2025). Advanced oxidation processes for the removal of antidepressants from wastewater: a comprehensive review. RSC Adv..

[cit50] Argurio P. (2018). *et al.*, Photocatalytic membranes in photocatalytic membrane reactors. Processes.

[cit51] An J. (2025). *et al.*, Design and engineering of photocatalytic graphitic carbon nitride for environmental and biological disinfection. ACS ES&T Eng..

[cit52] Xu P. (2020). *et al.*, The feasibility of ofloxacin degradation and electricity generation in photo-assisted microbial fuel cells with LiNbO3/CF photocatalytic cathode. Sep. Purif. Technol..

[cit53] Moghiseh Z., Almasi F. (2025). Metabolic activity and pathway study of emerging contaminants biodegradation using a photo-bioelectrochemical system: a review. 3 Biotech.

[cit54] Valenzuela L. (2024). *et al.*, An overview of the advantages of combining photo-and electrooxidation processes in actual wastewater treatment. Catalysts.

[cit55] Chhablani A., Jain V., Makasana J. (2025). Sulfonamides: Historical perspectives, therapeutic insights, applications, challenges, and synthetic strategies. ChemistrySelect.

[cit56] Mohammed H. H. (2019). *et al.*, Current trends and future directions of fluoroquinolones. Curr. Med. Chem..

[cit57] Zhang X. (2017). *et al.*, Occurrence, removal, and risk assessment of antibiotics in 12 wastewater treatment plants from Dalian, China. Environ. Sci. Pollut. Res..

[cit58] Larsson D. G. J., de Pedro C., Paxeus N. (2007). Effluent from drug manufactures contains extremely high levels of pharmaceuticals. J. Hazard. Mater..

[cit59] Gogić A. (2026). *et al.*, From Painkillers to Antidiabetics: Structural Modification of NSAID Scaffolds for Drug Repurposing. Future Pharmacol..

[cit60] Hamed M. M. (2023). *et al.*, Synthesis, biological evaluation, and molecular docking studies of novel diclofenac derivatives as antibacterial agents. J. Mol. Struct..

[cit61] Huynh N. C. (2023). *et al.*, Occurrence, toxicity, impact and removal of selected non-steroidal anti-inflammatory drugs (NSAIDs): A review. Sci. Total Environ..

[cit62] Baker M. E., Lathe R. (2018). The promiscuous estrogen receptor: Evolution of physiological estrogens and response to phytochemicals and endocrine disruptors. J. Steroid Biochem. Mol. Biol..

[cit63] Levin E. R. and Hammes S. R., Estrogens and progestins. Goodman & Gilman's The Pharmacological Basis of Therapeutics, 2011, vol. 12, pp. 1163–1194

[cit64] Yazdan M. M. S., Kumar R., Leung S. W. (2022). The Environmental and Health Impacts of Steroids and Hormones in Wastewater Effluent, as Well as Existing Removal Technologies: A Review. Ecologies.

[cit65] Mohagheghian A. (2014). *et al.*, Distribution of estrogenic steroids in municipal wastewater treatment plants in Tehran, Iran. J. Environ. Health Sci. Eng..

[cit66] Taddei S., Tsabedze N., Tan R.-S. (2024). β-blockers are not all the same: pharmacologic similarities and differences, potential combinations and clinical implications. Curr. Med. Res. Opin..

[cit67] Pirogov A. (2025). *et al.*, Highly-sensitive quantification of carbamazepine and identification of its degradation and metabolism products in human liver by high performance liquid chromatography–High resolution mass spectrometry. Toxicol Rep.

[cit68] Scheurink T. A. , *et al.*, Serum metabolic signatures of cognitive resilience in a longitudinal aging cohort, bioRxiv, 2026, preprint, 10.64898/2026.03.29.715122

[cit69] Ekpeghere K. I. (2018). *et al.*, Occurrence and distribution of carbamazepine, nicotine, estrogenic compounds, and their transformation products in wastewater from various treatment plants and the aquatic environment. Sci. Total Environ..

[cit70] Kostich M. S., Batt A. L., Lazorchak J. M. (2014). Concentrations of prioritized pharmaceuticals in effluents from 50 large wastewater treatment plants in the US and implications for risk estimation. Environ. Pollut..

[cit71] Björklund E., Svahn O. (2022). Total Release of 21 Indicator Pharmaceuticals Listed by the Swedish Medical Products Agency from Wastewater Treatment Plants to Surface Water Bodies in the 1.3 Million Populated County Skåne (Scania), Sweden. Molecules.

[cit72] Sirtori C. R. (2020). *et al.*, Recent advances in synthetic pharmacotherapies for dyslipidaemias. European Journal of Preventive Cardiology.

[cit73] Bakshi A. , *et al.*, Fate and behaviour of pharmaceutical and personal care products in wastewater, Detection and Treatment of Emerging Contaminants in Wastewater, 2024, p. 181

[cit74] Shah M. B. (2022). Inhibition of CYP2C8 by acyl glucuronides of gemfibrozil and clopidogrel: pharmacological significance, progress and challenges. Biomolecules.

[cit75] Hu Y. (2025). *et al.*, Iodinated contrast media (ICM)-induced thyroid dysfunction: a review of potential mechanisms and clinical management. Clin. Exp. Med..

[cit76] Sengar A., Vijayanandan A. (2021). Comprehensive review on iodinated X-ray contrast media: Complete fate, occurrence, and formation of disinfection byproducts. Sci. Total Environ..

[cit77] Kłysik K. (2020). *et al.*, Acyclovir in the treatment of herpes viruses–a review. Curr. Med. Chem..

[cit78] Kuroda K. (2021). *et al.*, Predicted occurrence, ecotoxicological risk and environmentally acquired resistance of antiviral drugs associated with COVID-19 in environmental waters. Sci. Total Environ..

[cit79] Sun Y. (2021). *et al.*, The influence of cell cycle regulation on chemotherapy. Int. J. Mol. Sci..

[cit80] Al-Jumaili M. H. A. (2025). *et al.*, Development of heterocyclic-based anticancer agents: A comprehensive review. Heterocycl. Commun..

[cit81] Zon G. (2021). Chemistry, Cyclophosphamide, Cancer Chemotherapy, and Serendipity: Sixty Years On. Substantia.

[cit82] Nassour C. (2020). *et al.*, Occurrence of anticancer drugs in the aquatic environment: a systematic review. Environ. Sci. Pollut. Res..

[cit83] Zheng J. (2019). *et al.*, Efficiency and stability of narrow-gap semiconductor-based photoelectrodes. Energy Environ. Sci..

[cit84] Qureshi A., Shaikh T., Niazi J. H. (2023). Semiconductor quantum dots in photoelectrochemical sensors from fabrication to biosensing applications. Analyst.

[cit85] Hussain S. A. (2024). *et al.*, Preparation of C-doped g-C3N4 by Co-polycondensation of melamine and sucrose for improved photocatalytic H2 evolution. Int. J. Hydrogen Energy.

[cit86] Alulema-Pullupaxi P. (2021). *et al.*, Fundamentals and applications of photoelectrocatalysis as an efficient process to remove pollutants from water: A review. Chemosphere.

[cit87] Liang X. (2025). *et al.*, Advanced TiO2-Based Photoelectrocatalysis: Material Modifications, Charge Dynamics, and Environmental–Energy Applications. Catalysts.

[cit88] Wang J. (2025). *et al.*, Transfer dynamics of photo-generated carriers in catalysis. Chem. Soc. Rev..

[cit89] Lee S., Bae H.-S., Choi W. (2023). Selective control and characteristics of water oxidation and dioxygen reduction in environmental photo (electro) catalytic systems. Acc. Chem. Res..

[cit90] Ta X. M. C. (2022). *et al.*, Alternatives to water photooxidation for photoelectrochemical solar energy conversion and green H2 production. Adv. Energy Mater..

[cit91] Gao X. W. (2024). *et al.*, Nanodiamond: a promising metal-free nanoscale material in photocatalysis and electrocatalysis. Rare Met..

[cit92] García-Eguizábal A. (2026). *et al.*, Stabilizing Cu-Based Photocathodes: From Interfacial Engineering to Advanced Architectures. Global Challenges.

[cit93] Hu Y. (2023). *et al.*, What controls direct hole-mediated oxidation kinetics in a carbon nitride-based photocatalytic system: a model study for aqueous aromatic compounds. ACS Catal..

[cit94] Geldasa F. T. (2023). *et al.*, Experimental and computational study of metal oxide nanoparticles for the photocatalytic degradation of organic pollutants: a review. RSC Adv..

[cit95] Chemin A. (2025). *et al.*, Modulating Surface Redox Reactions and Solvated Electron Emission on Boron-Doped Diamond by (Photo) electrochemistry. PRX Energy.

[cit96] Pareek A., Borse P. H. (2022). Hurdles and recent developments for CdS and chalcogenide-based electrode in “Solar electro catalytic” hydrogen generation: A review. Electrochem. Sci.
Adv..

[cit97] Li Z., Yan T., Fang X. (2023). Low-dimensional wide-bandgap semiconductors for UV photodetectors. Nat. Rev. Mater..

[cit98] Ding P. C. (2025). *et al.*, Anisotropy of Single-Crystal Semiconductors in Photo (electro) Catalysis. Angew. Chem., Int. Ed..

[cit99] Zhang H. (2024). *et al.*, Effective charge separation in photoelectrochemical water splitting: a review from advanced evaluation methods to materials design. Sustainable Energy Fuels.

[cit100] Chen L., Ren J. T., Yuan Z. Y. (2023). Enabling internal electric fields to enhance energy and environmental catalysis. Adv. Energy Mater..

[cit101] Li S. (2022). *et al.*, Recent progress on semiconductor heterojunction-based photoanodes for photoelectrochemical water splitting. Small Sci..

[cit102] Yue X., Fan J., Xiang Q. (2022). Internal electric field on steering charge migration: modulations, determinations and energy-related applications. Adv. Funct. Mater..

[cit103] Miao J. (2024). *et al.*, Improving charge transfer beyond conventional heterojunction photoelectrodes: fundamentals, strategies and applications. Adv. Funct. Mater..

[cit104] Ma Q. (2025). *et al.*, Photoelectrocatalytic degradation mechanism of fluorinated pollutants using a bilayer WO3 photoanode: synergistic role of holes and hydroxyl radicals. Angew. Chem., Int. Ed..

[cit105] Gao R.-T. (2024). *et al.*, Unveiling the promotion of dual-metal-atom Ir-Fe pair sites on charge transfer for photoelectrochemical water splitting. Matter.

[cit106] Jin C. (2024). *et al.*, Solar-driven photoelectrochemical conversion of biomass: recent progress, mechanistic insights and potential scalability. Energy Environ. Sci..

[cit107] Bondi L. (2024). *et al.*, Photovoltage Generation at p-Type Semiconducting Polymer/Electrolyte Interfaces. ACS Appl. Electron. Mater..

[cit108] Awan T. I. , Afsheen S. and Mushtaq A., Noble Metal Nanoparticles for Pollutant Decomposition, in Influence of Noble Metal Nanoparticles in Sustainable Energy Technologies, Springer, 2025, pp. 223–249

[cit109] Boochakiat S. (2023). *et al.*, Bismuth-based oxide photocatalysts for selective oxidation transformations of organic compounds. ChemNanoMat.

[cit110] Yang M. (2024). *et al.*, Emerging Advanced Photo-Rechargeable Batteries. Adv. Funct. Mater..

[cit111] Xu W. (2022). *et al.*, Asymmetric charge carrier transfer and transport in planar lead halide perovskite solar cells. Cell Rep. Phys. Sci..

[cit112] Wang T. (2022). *et al.*, Enhanced charge separation for efficient photocatalytic H2 production by long-lived trap-state-induced interfacial charge transfer. Nano Lett..

[cit113] Al-Senani G. M. (2026). *et al.*, Enhancing photoelectrochemical catalytic performance of zeolite-Co3O4 composites through optimized ball milling duration. Sci. Rep..

[cit114] Wang J. (2026). *et al.*, Hole Storage Layers in Photoelectrodes for Mitigating Recombination and Driving Stable PEC Water Splitting. Small.

[cit115] Reyes Cruz E. A. (2022). *et al.*, Molecular-modified photocathodes for applications in artificial photosynthesis and solar-to-fuel technologies. J. Chem. Rev..

[cit116] Yang H. (2023). *et al.*, Monolithic FAPbBr3 photoanode for photoelectrochemical water oxidation with low onset-potential and enhanced stability. Nat. Commun..

[cit117] Alley O. J. (2022). *et al.*, Best practices in PEC water splitting: how to reliably measure solar-to-hydrogen efficiency of photoelectrodes. Frontiers in Energy Research.

[cit118] Indah Sari F. N. (2025). *et al.*, Low-Cost, Earth-Abundant, and Eco-Friendly Fe-Containing Catalyst for Hydrogen Production via (Photo) electrolysis. Energy Fuels.

[cit119] Li Y. , *et al.*, Fe_3_O_4_/N-doped g-C3N4 composite biochar material activated with persulfate for photodegradation of oxytetracycline in aqueous systems. Available at SSRN: https://ssrn.com/abstract=6223242 or https://ssrn.com/abstract=6223242

[cit120] Dong C. (2021). *et al.*, Visible-light-driven peroxymonosulfate activation in photo-electrocatalytic system using hollow-structured Pt@CeO2@MoS2 photoanode for the degradation of pharmaceuticals and personal care products. Environ.
Int..

[cit121] Orimolade B. O. (2022). *et al.*, Peroxymonosulfate assisted photoelectrocatalytic degradation of pharmaceuticals at a FTO-Bi2WO6 electrode: Mechanism and kinetics studies. Catal. Commun..

[cit122] Xing Y. (2025). *et al.*, Construction of BiFeO3/BiVO4 composite photoanode and its application in PMS-assisted photoelectrocatalytic degradation of levofloxacin. J. Environ. Chem. Eng..

[cit123] Xing Y. (2026). *et al.*, Construction of FeOCl/BiVO4 photoanode for photoelectrocatalytic degradation of levofloxacin via PMS activation. J. Alloys Compd..

[cit124] Zheng Z. (2023). *et al.*, Facilitating peroxymonosulfate activation for effective antibiotics degradation from drinking water by photoelectrocatalytic system using MoS2 embedded carbon substrate. Chem. Eng. J..

[cit125] Dhawle R. (2023). *et al.*, Peroxymonosulfate enhanced photoelectrocatalytic degradation of 17α-ethinyl estradiol. Catal. Today.

[cit126] Song M. (2025). *et al.*, Photo-electrocatalysis assisted peroxymonsulfate activation by S-scheme BiVO4/Cu2O junction for efficiently tetracycline degradation. Appl. Surf. Sci..

[cit127] Li C. (2026). *et al.*, Photoelectrocatalytic Peroxymonosulfate activation over a {Mo72Fe30}/BiVO4 type-II heterojunction for efficient levofloxacin degradation under synergistic electric fields. Inorg. Chem. Commun..

[cit128] Wang W. (2026). *et al.*, Unraveling synergistic effect of ferric oxide modifying BiVO4 for durable photoelectrocatalytic activation of peroxymonosulfate for antibiotic removal. Appl. Surf. Sci..

[cit129] Pan T. (2023). *et al.*, BiVO4 modifying with cobalt-phosphate cluster cocatalyst for persulfate assisted photoelectrocatalytic degradation of tetracycline. Mol. Catal..

[cit130] Yuan W.-B. (2026). *et al.*, Double Z-scheme TiO2NTAs/Bi2WO6/BiOI ternary heterojunction for synergistic photoelectro-PDS degradation of ofloxacin. Appl. Surf. Sci..

[cit131] Son A. (2021). *et al.*, Persulfate enhanced photoelectrochemical oxidation of organic pollutants using self-doped TiO2nanotube arrays: Effect of operating parameters and water matrix. Water Res..

[cit132] Palomares-Reyna D. (2022). *et al.*, Photo-electrochemical and ozonation process to degrade ciprofloxacin in synthetic municipal wastewater, using C, N-codoped TiO2 with high visible-light absorption. J. Environ. Chem. Eng..

[cit133] Zhang B. (2022). *et al.*, Visible-light photoelectrocatalysis/H(2)O(2) synergistic degradation of organic pollutants by a magnetic Fe(3)O(4)@SiO(2)@mesoporous TiO(2) catalyst-loaded photoelectrode. RSC Adv..

[cit134] Yuan W.-B. (2025). *et al.*, Peroxydisulfate enhanced photoelectrocatalytic degradation of ofloxacin by double Z-scheme TiO2NTAs/CeO2/Bi2S3 heterojunction under visible light irradiation. J. Alloys Compd..

[cit135] Sheikhpour E. (2026). *et al.*, Hydrogen peroxide-assisted photoelectrocatalytic degradation of tetracycline by ZnFe2O4-decorated TiO2 nanotube arrays. Chem. Eng. J. Adv..

[cit136] Orimolade B. O. (2020). *et al.*, Coupling cathodic electro-fenton with anodic photo-electrochemical oxidation: A feasibility study on the mineralization of paracetamol. J. Environ. Chem. Eng..

[cit137] Liu J. (2024). *et al.*, Photoelectrocatalysis/photoelectro-Fenton system based on cone-like TiO2/nickel foam photoanode for efficient degradation of carbamazepine: Comparison with DSA. Chem. Eng. J..

[cit138] Jiang W.-L. (2020). *et al.*, A novel TiO2/graphite felt photoanode assisted electro-Fenton catalytic membrane process for sequential degradation of antibiotic florfenicol and elimination of its antibacterial activity. Chem. Eng. J..

[cit139] Palomares-Reyna D. (2022). *et al.*, Degradation of cefadroxil by photoelectrocatalytic ozonation under visible-light irradiation and single processes. J. Photochem. Photobiol., A.

[cit140] Zou R. (2021). *et al.*, A novel persulfate-photo-bioelectrochemical hybrid system promoting the degradation of refractory micropollutants at neutral pH. J. Hazard. Mater..

[cit141] Ren M.-Q. (2026). *et al.*, Synergistic antibiotic degradation by polydopamine/BiOBr-modified photocathode in self-powered photo-bio-electrochemical system. Bioresour. Technol..

[cit142] Koiki B. A. (2023). *et al.*, Sulfate radical in (photo) electrochemical advanced oxidation processes for water treatment: a versatile approach. J. Phys. Chem. Lett..

[cit143] Hu Y. (2025). *et al.*, Interface and defect engineering in 3D Co3O4-Ov/TiO2 to boost simultaneous removal of BPA and Cr (VI) upon photoelectrocatalytic/peroxymonosulfate (PEC/PMS) system. Adv. Funct. Mater..

[cit144] Zheng Z. (2023). *et al.*, Facilitating peroxymonosulfate activation for effective antibiotics degradation from drinking water by photoelectrocatalytic system using MoS2 embedded carbon substrate. Chem. Eng. J..

[cit145] Wang K. (2020). *et al.*, Peroxymonosulfate enhanced photoelectrocatalytic degradation of ofloxacin using an easily coated cathode. Sep. Purif. Technol..

[cit146] Thamilselvan A., Doong R.-A. (2023). Ni-Co bimetallic decorated dodecahedral ZIF as an efficient catalyst for photoelectrochemical degradation of sulfamethoxazole coupled with hydrogen production. Sci. Total Environ..

[cit147] Zhang J. (2025). *et al.*, Selective generation of Co (IV)= O in-situ on cobalt-doping MoS2@ CC photoanode through photoelectrochemical activation of peroxymonosulfate for efficient antibiotic degradation. Chem. Eng. J..

[cit148] Wu Q.-Y. (2023). *et al.*, Oxygen doping of cobalt-single-atom coordination enhances peroxymonosulfate activation and high-valent cobalt–oxo species formation. Proc. Natl. Acad. Sci. U. S. A..

[cit149] Guo J. (2025). *et al.*, Sustainable water decontamination: advanced high-valent iron active species-driven peroxymonosulfate activation for global challenges. CleanMat.

[cit150] Giannakis S., Lin K.-Y. A., Ghanbari F. (2021). A review of the recent advances on the treatment of industrial wastewaters by Sulfate Radical-based Advanced Oxidation Processes (SR-AOPs). Chem. Eng. J..

[cit151] Alam M. M., Das A., Adak A. (2024). Hydroxyl and sulfate radicals-based electrochemical advanced oxidation process for treating dye-bearing wastewater–A review. Water Practice & Technology.

[cit152] Liao C.-H., Kang S.-F., Wu F.-A. (2001). Hydroxyl radical scavenging role of chloride and bicarbonate ions in the H2O2/UV process. Chemosphere.

[cit153] Song H. (2020). *et al.*, Electrochemically activated PMS and PDS: Radical oxidation versus nonradical oxidation. Chem. Eng. J..

[cit154] Yang Y. (2017). *et al.*, Degradation of sulfamethoxazole by UV, UV/H2O2 and UV/persulfate (PDS): formation of oxidation products and effect of bicarbonate. Water Res..

[cit155] Dong T. (2025). *et al.*, Recent advances in oxidant-involved photoelectrochemical systems for sustainable wastewater treatment: mechanisms, applications, and perspectives. npj Mater. Sustainability.

[cit156] Wang M. (2023). *et al.*, Enhanced tetracycline degradation by NC codoped Fe2O3 with rich oxygen vacancies in peroxymonosulfate assisting photoelectrochemical oxidation system: performance, mechanism and degradation pathway. Chem. Eng. J..

[cit157] Zhang Y. (2019). *et al.*, Electricity generating & high efficiency advanced oxidation process including peroxymonosulfate activation in photocatalytic fuel cell. Chem. Eng. J..

[cit158] Ji J. (2023). *et al.*, In situ H2O2 generation and corresponding pollutant removal applications: a review. Chem.–Eur. J..

[cit159] Yamazaki S.-I. (2008). *et al.*, A fuel cell with selective electrocatalysts using hydrogen peroxide as both an electron acceptor and a fuel. J. Power Sources.

[cit160] Sun J. , *et al.*, Photoelectrochemical H2O2 Production from Oxygen Reduction, in Clean Energy Materials, ACS Publications, 2020, pp. 93–109

[cit161] Laskowski F. A. (2018). *et al.*, Metal oxide/(oxy) hydroxide overlayers as hole collectors and oxygen-evolution catalysts on water-splitting photoanodes. J. Am. Chem. Soc..

[cit162] Leitner N. K. V. , Sulfate radical ion–based AOPs, Advanced Oxidation Processes for Water Treatment, ed. M. I. Stefan, IWA Publishing, London, UK, 2018, pp. 429–460

[cit163] Ahmed N. (2021). *et al.*, A review on the degradation of pollutants by fenton-like systems based on zero-valent iron and persulfate: Effects of reduction potentials, pH, and anions occurring in waste waters. Molecules.

[cit164] Hirakawa T., Nosaka Y. (2002). Properties of O_2_˙^−^ and OH˙ formed in TiO2 aqueous suspensions by photocatalytic reaction and the influence of H2O2 and some ions. Langmuir.

[cit165] Wang Z. (2025). *et al.*, Challenges and prospects of catalyst design and environmental applications for on-site hydrogen peroxide production via diverse (photo) electrochemical reaction pathways. Small.

[cit166] Sun T. (2026). *et al.*, Design strategies and application progress of covalent organic frameworks in photocatalytic oxidation reactions. Chem. Sci..

[cit167] Soejima T., Naya S. I., Tada H. (2026). Photoelectrochemical Hydrogen Peroxide Production via Simultaneous Reduction and Oxidation Processes. Chem.–Eur. J..

[cit168] Collivignarelli M. C. (2020). *et al.*, Decolorization and biodegradability of a real pharmaceutical wastewater treated by H2O2-assisted photoelectrocatalysis on TiO2 meshes. J. Hazard. Mater..

[cit169] Li X. (2018). *et al.*, Synergetic activation of H2O2 by photo-generated electrons and cathodic Fenton reaction for enhanced self-driven photoelectrocatalytic degradation of organic pollutants. Appl. Catal., B.

[cit170] Li X. (2026). *et al.*, Catalytically Active Carbon for Electrochemical Production of H 2O_2_ via 2e^−^ ORR: Design Strategies, Catalytic Mechanisms, and Application Potential. Catal. Sci. Technol..

[cit171] Liu T. (2018). *et al.*, The role of reactive oxygen species and carbonate radical in oxcarbazepine degradation via UV, UV/H2O2: Kinetics, mechanisms and toxicity evaluation. Water Res..

[cit172] Xia C., Shen X. (2024). Analysis of factors influencing on Electro-Fenton and research on combination technology (II): a review. Environ. Sci. Pollut. Res..

[cit173] Sirés I. and Brillas E., The use of nanomaterials in electro-Fenton and photoelectro-Fenton processes, in Advanced Nano-Bio Technologies for Water and Soil Treatment, Springer, 2020, pp. 257–288

[cit174] Wu C. (2025). *et al.*, Enhanced Fe3+/Fe2+ cycle by a novel 2D/2D FeS/ACN composite for rapid photo-Fenton degradation of tetracycline-based wastewater. J. Environ. Manage..

[cit175] Huang Z. (2026). *et al.*, Degradation of per-and polyfluoroalkyl substances (PFAS) by Fenton reactions. Environ. Sci.: Adv..

[cit176] Zhang R., Liu Z., Chen T. (2024). Enhanced visible light photo-Fenton catalysis by Fe-doping oligo-layer natural molybdenite with efficient carrier spatial-driven Fe3+/Fe2+ cycle. Photochem. Photobiol..

[cit177] Zhao Q. (2026). *et al.*, Fenton-Assisted ZnIn2S4/C-PPy Cathode for Synergistic PVA Degradation and Photoelectrical Conversion in a Dual-Chamber Photocatalytic Fuel Cell. J. Environ. Chem. Eng..

[cit178] de Luna M. D. G. (2012). *et al.*, Acetaminophen degradation by electro-Fenton and photoelectro-Fenton using a double cathode electrochemical cell. J. Hazard. Mater..

[cit179] Wang Y. (2020). *et al.*, Nanocarbon-based catalytic ozonation for aqueous oxidation: engineering defects for active sites and tunable reaction pathways. ACS Catal..

[cit180] Hama Aziz K. H. (2019). Application of different advanced oxidation processes for the removal of chloroacetic acids using a planar falling film reactor. Chemosphere.

[cit181] De Brito J. F. (2019). *et al.*, Combination of photoelectrocatalysis and ozonation as a good strategy for organics oxidation and decreased toxicity in oil-produced water. J. Electrochem. Soc..

[cit182] Wang D. (2023). *et al.*, Degradation of odor compounds in drinking water by ozone and ozone-based advanced oxidation processes: a review. ACS ES&T Water.

[cit183] Xiao J. (2017). *et al.*, Fast electron transfer and ˙OH formation: key features for high activity in visible-light-driven ozonation with C3N4 catalysts. ACS Catal..

[cit184] Herrera-Chávez S. (2025). *et al.*, Hybrid Solar Photoelectro-Fenton and Ozone Processes for the Sustainable Removal of COVID-19 Pharmaceutical Contaminants. Processes.

[cit185] Dong T. (2025). *et al.*, Recent advances in oxidant-involved photoelectrochemical systems for sustainable wastewater treatment: mechanisms, applications, and perspectives. npj Mater. Sustainability.

[cit186] Oladipo A. A. (2026). Electro-Driven Membrane Separations for Sustainable Bio-Based Chemical Recovery: Energetics, Selectivity Engineering, Scale-Up Challenges, and Industrial Translation. Water.

[cit187] Khater D. Z. (2026). *et al.*, Microbial electrolysis cell (MEC) as a promising renewable clean technology for green hydrogen and value-added compounds production: towards achieving sustainable development goals. Materials for Renewable and Sustainable Energy.

[cit188] Noori M. T., Ganta A., Tiwari B. R. (2020). Recent advances in the design and architecture of bioelectrochemical systems to treat wastewater and to produce choice-based byproducts. J. Hazard., Toxic Radioact. Waste.

[cit189] Zhang M. (2019). *et al.*, Combined photoelectrocatalytic microbial fuel cell (PEC-MFC) degradation of refractory organic pollutants and in-situ electricity utilization. Chemosphere.

[cit190] Ding J. (2024). *et al.*, Bias-free glucose/O2 bio-photoelectrochemical system for multi-energy conversion and phenolic pollutant degradation. Biosens. Bioelectron..

[cit191] Nosaka Y., Nosaka A. Y. (2017). Generation and detection of reactive oxygen species in photocatalysis. J. Chem. Rev..

[cit192] Brillas E., Garcia-Segura S. (2023). Recent progress of applied TiO2 photoelectrocatalysis for the degradation of organic pollutants in wastewaters. J. Environ. Chem. Eng..

[cit193] Koiki B. A. (2023). *et al.*, Sulfate Radical in (Photo)electrochemical Advanced Oxidation Processes for Water Treatment: A Versatile Approach. J. Phys. Chem. Lett..

[cit194] Zhang J. (2026). *et al.*, Specific activation pathways of peroxymonosulfate to facilitate singlet oxygen evolution via hole-induced oxidation and hydroxyl radical disproportionation. Appl. Catal., B.

[cit195] Sahmi A. (2026). *et al.*, Advancements in sono-photo-electrocatalytic oxidation for the removal of toxic contaminants in wastewaters. Int. J. Environ. Sci. Technol..

[cit196] Tan X. (2026). *et al.*, Vacancy-confinement-mediated generation of surface-bound hydroxyl radical for enhanced photocatalytic degradation of perfluorooctanoic acid. Appl. Catal., B.

[cit197] Tian K. (2026). *et al.*, Surface-bound reactive oxygen species dominated catalytic ozonation for decontamination via CuO-Co3O4 heterostructure. Appl. Catal., B.

[cit198] Liang R. (2024). *et al.*, Novel photoelectrocatalytic system of oxygen vacancy-rich black TiO2-x nanocones photoanode and natural air diffusion cathode for efficient water purification and simultaneous H2O2 production. Appl. Catal., B.

[cit199] Pignatello J. J., Oliveros E., MacKay A. (2006). Advanced Oxidation Processes for Organic Contaminant Destruction Based on the Fenton Reaction and Related Chemistry. Crit. Rev. Environ. Sci. Technol..

[cit200] Lv X. H. (2024). *et al.*, The lifetime of hydroxyl radical in realistic fuel cell catalyst layer. ChemSusChem.

[cit201] Yu W. , *et al.*, Enhanced Photoelectrocatalytic Degradation of Salicylic Acid Using an nn Heterojunction NH2-La-MOFs@B-GQDs@NTFs/Ti Photoelectrode. Available at SSRN: https://ssrn.com/abstract=5817528 or http://dx.doi.org/10.2139/ssrn.5817528

[cit202] Karimi-Shamsabadi M., Nezamzadeh-Ejhieh A. (2016). Comparative study on the increased photoactivity of coupled and supported manganese-silver oxides onto a natural zeolite nano-particles. J. Mol. Catal. A: Chem..

[cit203] Gligorovski S. (2015). *et al.*, Environmental implications of hydroxyl radicals (˙OH). J. Chem. Rev..

[cit204] Yang Y., Pignatello J. J. (2017). Participation of the halogens in photochemical reactions in natural and treated waters. Molecules.

[cit205] Hamdaoui O., Alghyamah A. (2025). Scavenger-Probed Mechanisms in the Ultrasound/Chlorine Sono-Hybrid Advanced Oxidation Process. Catalysts.

[cit206] Micheletto J. (2020). *et al.*, The solar photo-Fenton process at neutral pH applied to microcystin-LR degradation: Fe2+, H2O2 and reaction matrix effects. Photochem. Photobiol. Sci..

[cit207] Giannakis S. (2018). Analogies and differences among bacterial and viral disinfection by the photo-Fenton process at neutral pH: a mini review. Environ. Sci. Pollut. Res..

[cit208] Koiki B. A., Arotiba O. A. (2023). Peroxydisulphate activated FTO-WO3 nanorods based photoelectrocatalytic degradation of tetracycline: Intermediate products, degradation pathway and ecotoxicity studies. Heliyon.

[cit209] Zhang X. (2025). *et al.*, Enhanced mechanism of a TiO 2/gC 3 N 4 photoanode in photoelectrocatalytic synergistic persulfate activation for antibiotic degradation. RSC Adv..

[cit210] Tian L. (2021). *et al.*, Selective oxidation of diclofenac sodium with different electronegative moieties via coexisting SO4− and OH. Sci. Total Environ..

[cit211] Duan X. (2022). *et al.*, Comparison of sulfate radical with other reactive species. Curr. Opin. Chem. Eng..

[cit212] Wei W. (2026). *et al.*, Molecular Oxygen Activation for Organic Pollutants Degradation in Water: Strategies, Mechanisms, and Applications. Catalysts.

[cit213] Wolfrum E. J. and Ollis D. F., Hydrogen peroxide in heterogeneous photocatalysis, Aquatic and Surface Photochemistry, 2018, pp. 451–466

[cit214] Goncharuk V., Soboleva N., Nosonovich A. (2010). Photooxidative destruction of organic compounds by hydrogen peroxide
in water. Journal of Water Chemistry and Technology.

[cit215] Zhang Y., Dai M., Yuan Z. (2018). Methods for the detection of reactive oxygen species. Anal. Methods.

[cit216] Yi Y. (2013). *et al.*, Safe direct synthesis of high purity H2O2 through a H2/O2 plasma reaction. Angew. Chem., Int. Ed..

[cit217] Wang C., Xiao J. (2025). Activation of molecular oxygen and selective oxidation with metal complexes. Acc. Chem. Res..

[cit218] Sarfraz H. (2026). *et al.*, Carbon–carbon bond formation reaction overactive sites of heterogeneous catalysts: understanding the role of catalytic sites with reaction mechanism, performance, stability, and product selectivity. Pure Appl. Chem..

[cit219] Zhang X. (2024). *et al.*, Tetracycline degradation through selective activation of molecular oxygen by a fluidic sequential photoelectro-Fenton system. Sep. Purif. Technol..

[cit220] Kong D. (2025). *et al.*, Revisiting the design of direct-electron-transfer oxidation systems: Synergistic roles of thermodynamic and hydrodynamic properties. Appl. Catal., B.

[cit221] Liu J. (2024). *et al.*, Heterogeneous catalysis for the environment. Innovation Mater..

[cit222] Ahmed M. A., Mahmoud S. A., Mohamed A. A. (2025). Interfacially engineered metal oxide nanocomposites for enhanced photocatalytic degradation of pollutants and energy applications. RSC Adv..

[cit223] Hussain A. (2024). *et al.*, Recent advances in BiOX-based photocatalysts to enhanced efficiency for energy and environment applications. Catal. Rev..

[cit224] Liu J. (2026). *et al.*, Electron-transfer management at carbon–iron–oxidant interfaces: carbon architectures bridging radical/non-radical pathways for selective, self-sustained advanced oxidation. Environ. Sci.: Nano.

[cit225] Meng Y. (2024). *et al.*, Nanoconfinement steers nonradical pathway transition in single atom fenton-like catalysis for improving oxidant utilization. Nat. Commun..

[cit226] Mao H. , *et al.*, Construction and Enhancement of Internal Electric Field In Fe-G-C3n4/Bi2wo6(O) Heterostructure with Oxygen Vacancies for Fenton-Like Degradation of Tetracycline and Photo-Fenton Treatment of Complex Wastewater, Available at SSRN: https://ssrn.com/abstract=5202714 or http://dx.doi.org/10.2139/ssrn.5202714

[cit227] Sathiyan K. (2024). *et al.*, Revisiting the electron transfer mechanisms in Ru (III)-mediated advanced oxidation processes with peroxyacids and ferrate (VI). Environ. Sci. Technol..

[cit228] Li Z., Zhu Y., Matson J. B. (2022). pH-responsive self-assembling peptide-based biomaterials: designs and applications. ACS Appl. Bio Mater..

[cit229] Alabdulrahem M. A. , Alaqeel M. A. and Saleh T. A., Nanostructure-Based Materials for Sorption and Photoelectrochemical Degradation of Water Pollutants: Water Purification Techniques, in Breakthroughs in Water Engineering, Treatment, and Hydraulics through Nanomaterial Innovation, IGI Global Scientific Publishing, 2026, pp. 75–120

[cit230] Ahmed H. R., Ealias A. M. (2026). Comparative evaluation of persulfate and peroxy monosulfate-based advanced oxidation processes for amoxicillin degradation: mechanisms, efficiency, and challenges. RSC Adv..

[cit231] Li C. (2026). *et al.*, Photoelectrocatalytic Peroxymonosulfate activation over a {Mo72Fe30}/BiVO4 type-II heterojunction for efficient levofloxacin degradation under synergistic electric fields. Inorg. Chem. Commun..

[cit232] Machreki M. (2024). *et al.*, Photoelectrochemical activation of peroxymonosulfate using Sn-doped α-Fe2O3 thin film for degradation of anti-inflammatory pharmaceutical drug. J. Photochem. Photobiol., A.

[cit233] Skoumal M. (2009). *et al.*, Electro-Fenton, UVA photoelectro-Fenton and solar photoelectro-Fenton degradation of the drug ibuprofen in acid aqueous medium using platinum and boron-doped diamond anodes. Electrochim. Acta.

[cit234] Qin W. (2025). *et al.*, Enhanced acetaminophen degradation by photoelectro-persulfate system with 3D nanostructured PbO2/Sb-SnO2//blue-TiO2//WO3 bifacial photoanode and Mo-CuFeO2/CF cathode. Sep. Purif. Technol..

[cit235] Dhawle R. (2023). *et al.*, Peroxymonosulfate enhanced photoelectrocatalytic degradation of 17α-ethinyl estradiol. Catal. Today.

[cit236] Wang A., Li Y.-Y., Estrada A. L. (2011). Mineralization of antibiotic sulfamethoxazole by photoelectro-Fenton treatment using activated carbon fiber cathode and under UVA irradiation. Appl. Catal., B.

[cit237] Xing Z., Wang Z.-J., Yang S. (2022). Capturing solar energy for cathodic protection of metals: the life of photoexcited charge carriers. Adv. Energy Sustainability Res..

[cit238] Cho Y., Le T. A., Lee H. (2020). Understanding surface modulation to improve the photo/electrocatalysts for water oxidation/reduction. Molecules.

[cit239] Toqeer I. (2026). Ferrite-Based photocatalysts for environmental remediation and solar-driven energy conversion. Environ. Technol. Rev..

[cit240] Pang L. (2025). *et al.*, The activation mechanisms of permonosulfate and pollutant degradation pathways by generated reactive species. Environ. Sci.: Nano.

[cit241] Santos A. , Lorenzo D. and Dominguez C. M., Persulfate in remediation of soil and groundwater contaminated by organic compounds, in Electrochemically Assisted Remediation of Contaminated Soils: Fundamentals, Technologies, Combined Processes and Pre-pilot and Scale-Up Applications, Springer, 2021, pp. 221–262

[cit242] Ai S. (2025). *et al.*, Photocatalytic Activation of Peroxodisulfate over the CuFe2O4/C/SiO2 Nanocomposite for Tetracycline Hydrochloride Robust Degradation. ACS Omega.

[cit243] Wang J. L., Xu L. J. (2012). Advanced oxidation processes for wastewater treatment: formation of hydroxyl radical and application. Crit. Rev. Environ. Sci. Technol..

[cit244] Koiki B. A. (2021). *et al.*, Sulphate radical enhanced photoelectrochemical degradation of sulfamethoxazole on a fluorine doped tin oxide-copper (I) oxide photoanode. J. Electroanal. Chem..

[cit245] Palomares-Reyna D. (2022). *et al.*, Photo-electrochemical and ozonation process to degrade ciprofloxacin in synthetic municipal wastewater, using C, N-codoped TiO2 with high visible-light absorption. J. Environ. Chem. Eng..

[cit246] Lee Y., Khim J. (2021). Effects of electrolyte concentration and anion identity on photoelectrochemical degradation of phenol: Focusing on the change at the photoanode/solution interface. J. Environ. Chem. Eng..

[cit247] De Laat J. and Stefan M., UV/Chlorine process, Advanced Oxidation Processes for Water Treatment: Fundamentals and Applications, 2017, vol. 16, pp. 383–428

[cit248] Deblonde G. J.-P. (2023). *et al.*, Impact of a biological chelator, lanmodulin, on minor actinide aqueous speciation and transport in the environment. Environ. Sci. Technol..

[cit249] Paiu M., Favier L., Gavrilescu M. (2025). Assessing the Ecotoxicological Effects of Emerging Drug and Dye Pollutants on Plant–Soil Systems Pre-and Post-Photocatalytic Wastewater Treatment. Plants.

[cit250] Konstantinou I. K., Antonopoulou M., Lambropoulou D. A. (2014). Transformation products of emerging contaminants formed during advanced oxidation processes. Transform. Prod. Emerging Contam. Environ..

[cit251] Echeverri-Jaramillo G. (2020). *et al.*, Acute toxicity of chlorpyrifos and its metabolite 3, 5, 6-trichloro-2-pyridinol alone and in combination using a battery of bioassays. Environ. Sci. Pollut. Res..

[cit252] Rodrigues S., Alves R. S., Antunes S. C. (2025). Impact of caffeine on aquatic ecosystems: assessing Trophic-Level biological responses. J. Xenobiot..

[cit253] de Souza C. S. R. (2025). *et al.*, Ecotoxicity of Entecavir in Aliivibrio fischeri, Microcystis novacekii, Artemia salina, and Biomphalaria glabrata. ACS Chem. Health Saf..

[cit254] Priac A., Badot P.-M., Crini G. (2017). Treated wastewater phytotoxicity assessment using Lactuca sativa: focus on germination and root elongation test parameters. C. R. Biol..

[cit255] Cardoso R. (2024). *et al.*, Effective degradation of phenacetin in wastewater by (photo) electro-Fenton processes: Investigation of variables, acute toxicity, and intermediates. J. Environ. Chem. Eng..

[cit256] Gowland D. , Sustainable Methods for the Removal of Bacteria and NOM from Drinking Water, 2024

[cit257] Basu S. , *et al.*, An Overview of Chemistry and Microbiology of Water, Water Management in Petroleum Industries, 2025, pp. 1–59

[cit258] Dhiman N. , Bharti B. and Singla N., Current Advances in Water Remediation Approaches, Biotechnology Innovations for a Sustainable Future: Integrating Clean Energy, Life on the Planet, Clean Water, and Climate Action, 2026, pp. 1163–1195

[cit259] Weng B. (2025). *et al.*, Photo-assisted technologies for environmental remediation. Nature Reviews Clean Technology.

[cit260] Dong C. (2021). *et al.*, Visible-light-driven peroxymonosulfate activation in photo-electrocatalytic system using hollow-structured Pt@ CeO2@ MoS2 photoanode for the degradation of pharmaceuticals and personal care products. Environ. Int..

[cit261] Liu H., Wang C., Wang G. (2020). Photocatalytic advanced oxidation processes for water treatment: recent advances and perspective. Chem.–Asian J..

[cit262] Khan A., Ersoz M. (2026). Unveiling the photocatalyst surface poisoning during water splitting: mechanisms, theoretical modeling and anti-poisoning strategies. Catal. Sci. Technol..

[cit263] Yu J. (2024). *et al.*, Basic comprehension and recent trends in photoelectrocatalytic systems. Green Chem..

[cit264] Marquez R. A. (2025). *et al.*, Insights into catalyst degradation during alkaline water electrolysis under variable operation. Energy Environ. Sci..

[cit265] Hu H., Huang J. (2026). Engineering pathway control in single-atom catalysis for advanced oxidation and electro-oxidation water treatment. Catal. Sci. Technol..

[cit266] Keen O. (2018). *et al.*, Standard reporting of electrical energy per order (E EO) for UV/H2O2 reactors (IUPAC technical report). Pure Appl. Chem..

[cit267] Willis D. E. (2023). *et al.*, Efficient chemical-free degradation of waterborne micropollutants with an immobilized dual-porous TiO2 photocatalyst. ACS ES&T Eng..

[cit268] Barazesh J. M. (2015). *et al.*, Modular advanced oxidation process enabled by cathodic hydrogen peroxide production. Environ. Sci. Technol..

[cit269] Shao Y. (2019). *et al.*, Efficient degradation of acesulfame by ozone/peroxymonosulfate advanced oxidation process. Molecules.

[cit270] Fu J. (2022). *et al.*, Electrochemical activation of peroxymonosulfate (PMS) by carbon cloth anode for sulfamethoxazole degradation. Chemosphere.

[cit271] Kong K. (2026). *et al.*, Understanding the Defluorination Mechanism of Per-and Polyfluoroalkyl Substances in Wastewater: From Microscopic Process to Practical Application. Small.

[cit272] Collivignarelli M. C. (2021). *et al.*, Efficiency and energy demand in polishing treatment of wastewater treatment plants effluents: Photoelectrocatalysis vs. Photocatalysis and photolysis. Water.

[cit273] Collivignarelli M. C. (2021). *et al.*, Photoelectrocatalysis on TiO(2) meshes: different applications in the integrated urban water management. Environ. Sci. Pollut. Res. Int..

[cit274] Bugueño-Carrasco S. (2021). *et al.*, Elimination of pharmaceutical pollutants by solar photoelectro-Fenton process in a pilot plant. Environ. Sci. Pollut. Res. Int..

